# Prediction of prognosis and immunotherapy efficacy based on metabolic landscape in lung adenocarcinoma by bulk, single-cell RNA sequencing and Mendelian randomization analyses

**DOI:** 10.18632/aging.205838

**Published:** 2024-05-20

**Authors:** Yong Liu, Xiangwei Zhang, Zhaofei Pang, Yadong Wang, Haotian Zheng, Guanghui Wang, Kai Wang, Jiajun Du

**Affiliations:** 1 Institute of Oncology, Shandong Provincial Hospital, Shandong University, Jinan 250021, Shandong, China; 2 Department of Thoracic Surgery, Shandong Provincial Hospital Affiliated to Shandong First Medical University, Jinan 250021, Shandong, China; 3 Department of Oncology, Shandong Provincial Hospital Affiliated to Shandong First Medical University, Jinan 250021, Shandong, China; 4 Institute of Oncology, Shandong Provincial Hospital Affiliated to Shandong First Medical University, Jinan 250021, Shandong, China

**Keywords:** metabolic reprogramming, prognosis, tumor microenvironment, immunotherapy, lung adenocarcinoma

## Abstract

Immunotherapy has been a remarkable clinical advancement in cancer treatment, but only a few patients benefit from it. Metabolic reprogramming is tightly associated with immunotherapy efficacy and clinical outcomes. However, comprehensively analyzing their relationship is still lacking in lung adenocarcinoma (LUAD). Herein, we evaluated 84 metabolic pathways in TCGA-LUAD by ssGSEA. A matrix of metabolic pathway pairs was generated and a metabolic pathway-pair score (MPPS) model was established by univariable, LASSO, multivariable Cox regression analyses. The differences of metabolic reprogramming, tumor microenvironment (TME), tumor mutation burden and drug sensitivity in different MPPS groups were further explored. WGCNA and 117 machine learning algorithms were performed to identify MPPS-related genes. Single-cell RNA sequencing and *in vitro* experiments were used to explore the role of C1QTNF6 on TME. The results showed MPPS model accurately predicted prognosis and immunotherapy efficacy of LUAD patients regardless of sequencing platforms. High-MPPS group had worse prognosis, immunotherapy efficacy and lower immune cells infiltration, immune-related genes expression and cancer-immunity cycle scores than low-MPPS group. Seven MPPS-related genes were identified, of which C1QTNF6 was mainly expressed in fibroblasts. High C1QTNF6 expression in fibroblasts was associated with more infiltration of M2 macrophage, Treg cells and less infiltration of NK cells, memory CD8+ T cells. *In vitro* experiments validated silencing C1QTNF6 in fibroblasts could inhibit M2 macrophage polarization and migration. The study depicted the metabolic landscape of LUAD and constructed a MPPS model to accurately predict prognosis and immunotherapy efficacy. C1QTNF6 was a promising target to regulate M2 macrophage polarization and migration.

## INTRODUCTION

Lung cancer remains a leading cause of cancer-related death worldwide [1]. Lung adenocarcinoma (LUAD) is the most common histological subtype, accounting for 40% lung cancer cases [2]. Although great progress has been made in LUAD treatment, the five-year survival rate of patients remains dismal [3]. Immunotherapy has led to striking clinical improvements while not all cancer patients can benefit from immunotherapy due to heterogeneity and adaptive evolution of tumor. Only about one third of patients acquire durable alleviation from it [4]. To give patients more personalized medicine, it is essential to reveal the mechanism underlying distinct immunotherapy responses and develop signatures to predict prognosis and immunotherapy efficacy.

Recent studies revealed that oncogenic transformation induces a well-characterized metabolic phenotype in tumor cells, which in turn affects tumor microenvironment (TME) [5]. As a new hallmark of malignant tumors, metabolic reprogramming improves malignant cells adaptation to meet bioenergetic, biosynthetic, redox balance demands and immune evasion, thus providing a selective advantage during tumorigenesis [5]. Aerobic glycolysis (the Warburg effect) is a special metabolic pattern that tumor cells consume glucose and produces lactate even when oxygen is sufficient. Aerobic glycolysis not only provides enough ATP but also numerous precursor metabolites for lipids, amino acids, and nucleotides biosynthesis to support rapid proliferation [6]. Dysregulated lipid metabolism is another prominent metabolic alteration in cancer [7]. Under energy stressful conditions, tumor cells can harness lipid hydrolyzation to generate ATP and second messengers including diacylglycerol, arachidonic acid, lysophosphatidic acid, and phosphatidic acid to activate oncogenic signaling pathways [7–9]. Other metabolic pathways such as amino acids metabolism, one carbon metabolism, purine and pyrimidine metabolism, are also dysregulated in tumor cells due to mutation of oncogenes, tumor suppressor genes or metabolic enzymes [10, 11]. Increasing evidence has suggested that tumor metabolic heterogeneity is greatly associated with TME status and immunotherapy [12–14]. Several studies have suggested that glycolysis of tumor cells restricts glucose utility of tumor-infiltrating lymphocytes, thereby inducing T cells exhaustion and immune escape [15]. Glutamine deprivation inhibits the transformation of CD4^+^ T cells to inflammatory subtypes, production and secretion of pro-inflammatory cytokines (IL-1, IL-6, and TNF) by macrophages, and promotes the apoptosis of immune cells [16–18]. Large amount of lactic acid produced by tumor cells increases the acidity of TME and impairs the anti-tumor function of T cells and natural killer (NK) cells [19, 20]. Consequently, comprehensively depicting tumor metabolic landscape is promising to predict the prognosis and immunotherapy response of cancer patients and develop new treatment strategies.

Based on some metabolic features, many metabolic signature-based prognostic models have been established and acquired good predicting performance [14, 21, 22]. However, most models are based on a single metabolic pathway, lacking comprehensive exploration for tumor metabolism. Moreover, most models are constructed by focusing on exact gene expression and commonly unapplicable in another cohort sequenced by different platforms. To overcome the above shortcomings, we comprehensively assessed 84 metabolic pathways from 12 kinds of metabolism in LUAD by single sample gene set enrichment analysis (ssGSEA), developed and validated a metabolic pathway-pair score (MPPS) model to accurately predict the prognosis and immunotherapy efficacy of LUAD patients regardless of sequencing platforms. The model performed better than 51 published signatures of LUAD and was applicable to pan-cancers. The distinct metabolic features, TME between high- and low-MPPS groups were depicted. Weighted gene co-expression network analysis (WGCNA) and 117 machine learning algorithm combinations were performed and identified 7 MPPS-related genes, of which C1QTNF6 was mainly expressed in fibroblast. C1QTNF6 expression in fibroblast is positively related to fibroblasts, M2 macrophages and Treg cells infiltration but negatively related to memory CD8^+^ T cells and NK cells infiltration. Silencing C1QTNF6 expression in fibroblast impaired M2 macrophage polarization and migration *in vitro* assays. Meanwhile, mendelian randomization (MR) also indicated that C1QTNF6 was cause of lung cancer onset. The MPPS model overcomes the obstacle of sequencing data from different platforms and is promising to guide LUAD patients’ selection for immunotherapy.

## RESULTS

### Heterogenous metabolic profiles of LUAD

**Figure 1:**
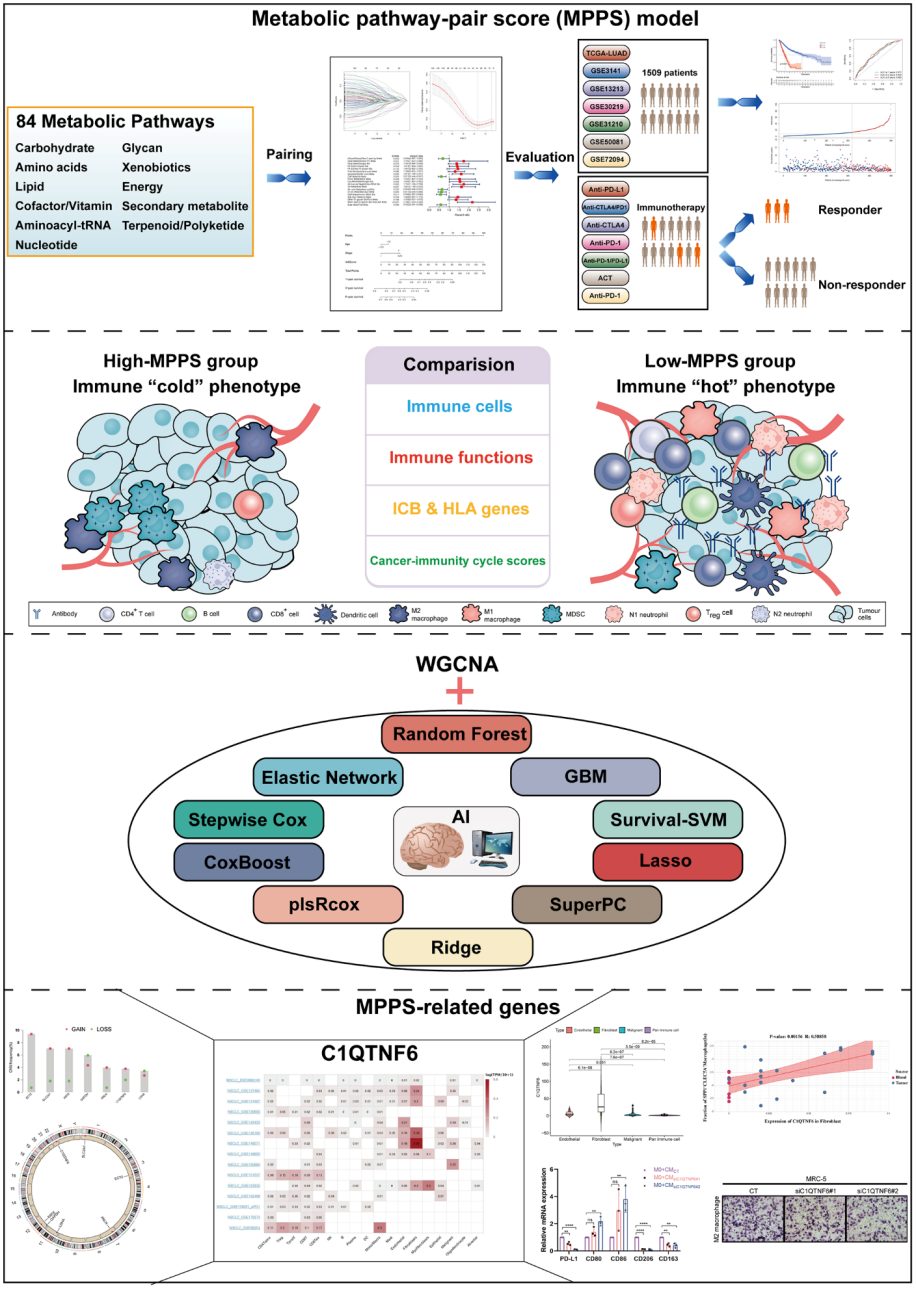
The flow chart of the study.

**Figure 2:**
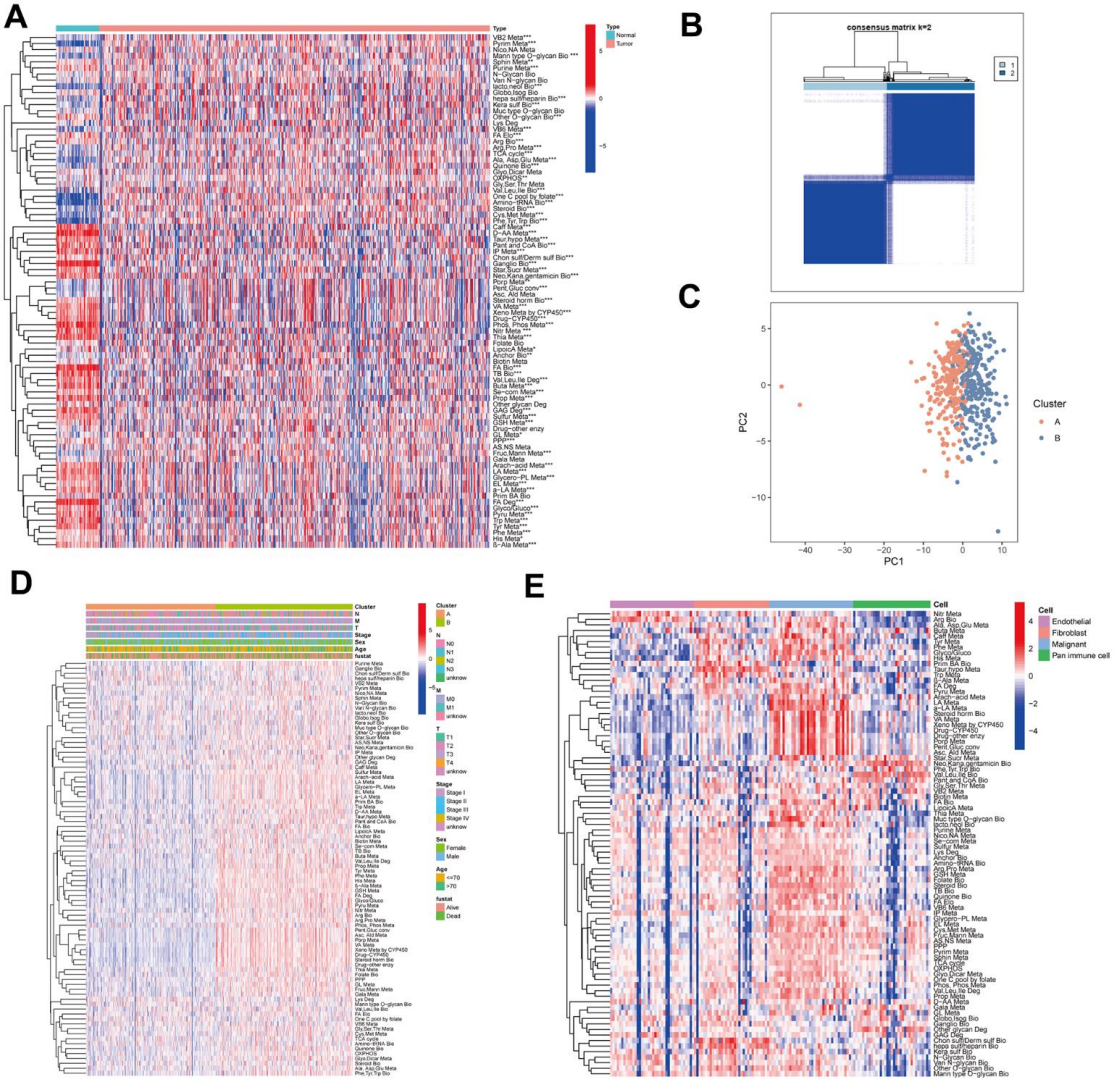
**The metabolic heterogeneity of lung adenocarcinoma (LUAD).** (**A**) The differences of 84 metabolic pathways scores between LUAD and normal tissues in TCGA. (**B**) An unsupervised consensus clustering according to 84 metabolic pathways scores in TCGA-LUAD samples. (**C**) Principal Component Analysis of cluster A and B of TCGA-LUAD. (**D**) The differences of 84 metabolic pathways scores between cluster A and cluster B. Pathological stage, sex, age, and survival status used as patients’ annotation. (**E**) The differences of 84 metabolic pathways scores among different cells by single-cell RNA sequencing (scRNA-seq) data. *P < 0.05, **P < 0.01, ***P < 0.001.

The overall design of our study was shown in the flow chart (Figure 1). To investigate the metabolic reprogramming in LUAD, we extracted 84 metabolic pathways from the Kyoto Encyclopedia of Genes and Genomes (KEGG) database. The 84 metabolic pathways contained 12 kinds of metabolism, including 14 carbohydrate metabolic pathways, 13 lipid metabolic pathways, 12 cofactors and vitamins metabolic pathway, 13 amino acid metabolic pathways, 14 glycan biosynthesis and metabolic pathways, 2 biosynthesis pathways of other secondary metabolites, 3 energy metabolic pathways, 1 genetic information processing pathway, 6 other amino acids metabolic pathways, 1 terpenoids and polyketides metabolic pathway, 2 nucleotide metabolic pathway and 3 xenobiotics biodegradation and metabolic pathway. We first scored each pathway in all samples using ssGSEA and characterized the metabolic heterogeneity between LUAD and normal tissues (Figure 2A). It was demonstrated that a total of 68 (80.95%) metabolic pathways were dramatically dysregulated (23 upregulated pathways and 45 downregulated pathways in LUAD compared to normal lung tissues) (*P* < 0.05). The dysfunctional pathways encompassed the three main kinds of metabolism including carbohydrate, lipid and amino acids metabolism. Next, to investigate the intratumor heterogeneity of metabolism, we classified TCGA-LUAD samples into two clusters based on 84 metabolic pathways scores by unsupervised consensus clustering (Figure 2B). PCA and metabolic heatmap displayed that the two clusters had obviously heterogenous metabolic characteristics (Figure 2C, 2D). The cluster A seemed to be a “cold” metabolic subtype but the cluster B seemed to a “hot” metabolic subtype. To further distinguish the metabolic variation in various TME cells, we compared metabolism scores among endothelial cells, fibroblast, malignant cells and pan-immune cells by scRNA-seq data. The results suggested that malignant cells had a significantly higher metabolic level than the other three cells (Figure 2E). The above results indicated that metabolic heterogeneity was common in LUAD and might play a crucial role in the initiation and progression of LUAD.

### Development of a prognostic model based on MPPS and exploration of its clinical relevance

**Figure 3:**
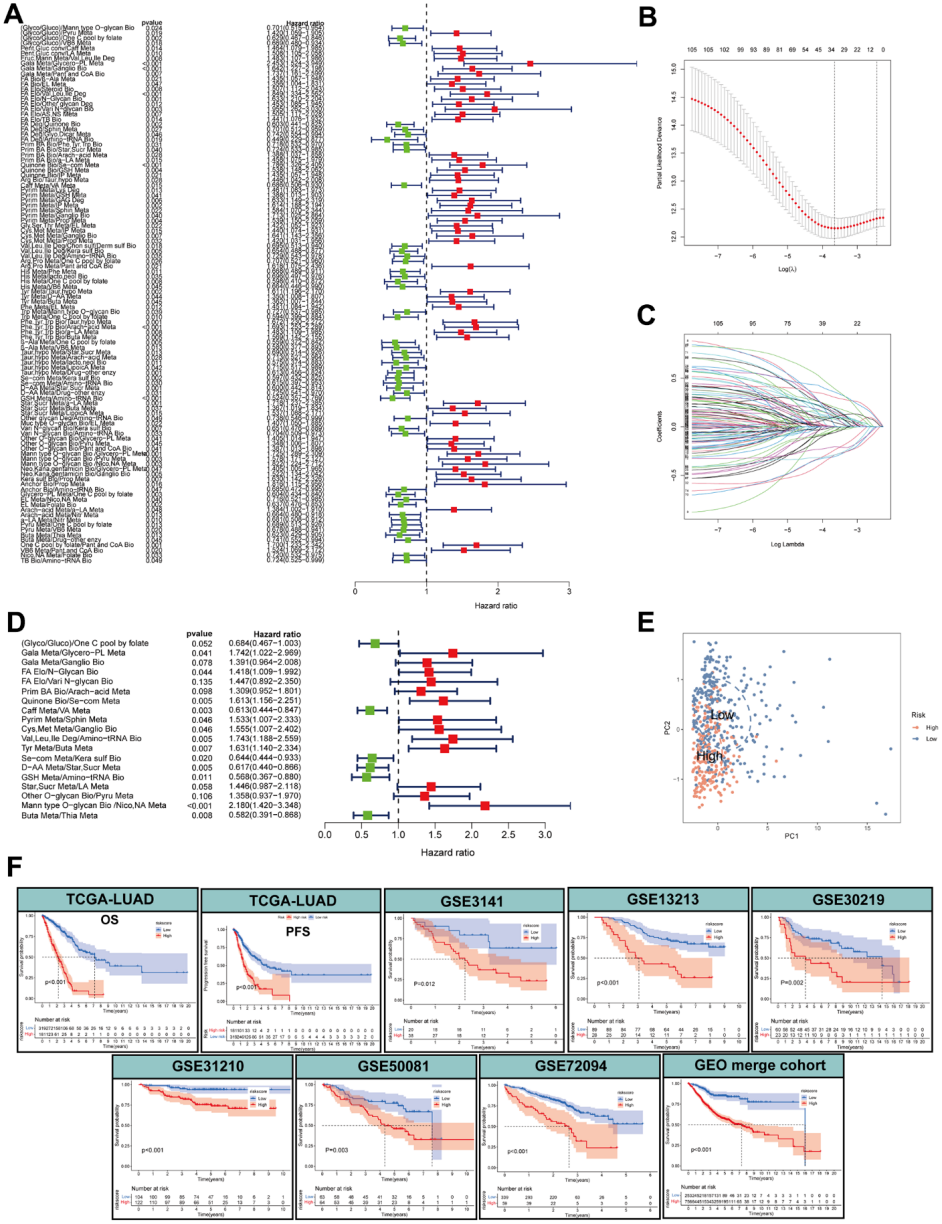
**Construction of a metabolic pathway-pair score (MPPS)-based prognostic model.** (**A**) The univariable Cox regression analysis of metabolic pathway pairs. (**B**, **C**) Determination of the number of metabolic pathway pairs by the LASSO regression analysis. (**D**) The multivariable Cox regression analysis of metabolic pathway pairs. (**E**) Principal Component Analysis of the high- and low-risk groups. (**F**) The Kaplan-Meier analysis of the high- and low-risk groups in TCGA-LUAD cohort and GEO validation cohorts. OS: overall survival; PFS: progression-free survival. The survival analysis was tested by log-rank test.

Different sequencing platforms commonly possess different sequencing depth. Consequently, sequencing data from different sequencing platforms had different numbers of genes and significantly different expression levels. When gene expression data from different platforms were utilized, data standardization and scaling and intersecting gene from different platforms are needed, which will cause loss of some genetic information. The metabolic pathway pair model can reduce the effects of some gene deletion on prediction and eliminate the shortcomings of data standardization and scaling in gene expression data processing and effectively avoid the interference caused by the sequencing platform. To develop a prognostic model based on MPPS, we firstly paired the 84 pathways and 525 pathway pairs were obtained after removing those pathway pairs that proportion of 0 or 1 was more than 80% or less than 20%. Subsequently, we conducted univariable Cox regression analysis on these pathway pairs and selected 106 meaningful metabolic pathway pairs for Least absolute shrinkage and selection operator (LASSO) regression analysis (Figure 3A). LASSO regression analysis yielded 33 metabolic pathway pairs with nonzero LASSO coefficients according to the optimal λ value (Figure 3B, 3C). Multivariable Cox regression analysis was further performed to identify prognostic metabolic pathway pairs based on Akaike information criterion value and 19 metabolic pathway pairs were finally obtained (Figure 3D). MPPS was calculated using value of 19 metabolic pathway pairs weighted by their multivariable Cox regression coefficients and stratified LUAD patients into high- and low-MPPS groups according to the optimal cut-off point determined by the “survminer” package. PCA analysis showed that LUAD patients could be divided into distinctive groups according to MPPS (Figure 3E). The heatmap showed obvious discrepancy of 19 metabolic pathway pairs between the high- and low-MPPS group (Supplementary Figure 1A). Patients with high-MPPS scores had significantly shortened overall survival (OS) and progression-free survival (PFS) in the TCGA-LUAD training cohort and six GEO validation cohorts (all *P* < 0.05). The GEO merge cohort integrating the six GEO cohorts also showed the same trend (*P* < 0.05) (Figure 3F). The risk plot of MPPS indicated that as MPPS increased, OS time decreased while mortality rose (Supplementary Figure 1B).

To determine the correlation of MPPS and clinical traits, we compared the differences in MPPS among different clinical subgroups based on age, sex, survival status and pathological stage. Patients in alive, stage I, stage T1 and stage N0 subgroups had lower MPPS compared to the other subgroups (*P* < 0.05), while there was no significant difference of MPPS in age, sex and M stage subgroups (Supplementary Figure 2A–2G). The Sankey diagram illustrated the distribution and correspondence of LUAD patients in MPPS groups, survival status, age, sex, and pathological stage (Supplementary Figure 2H). In addition, MPPS also showed robust performance on predicting prognosis in different clinical subgroups, including age, sex, TNM stage (*P* < 0.05) (Supplementary Figure 2I–2P).

### Evaluation of the MPPS model

**Figure 4:**
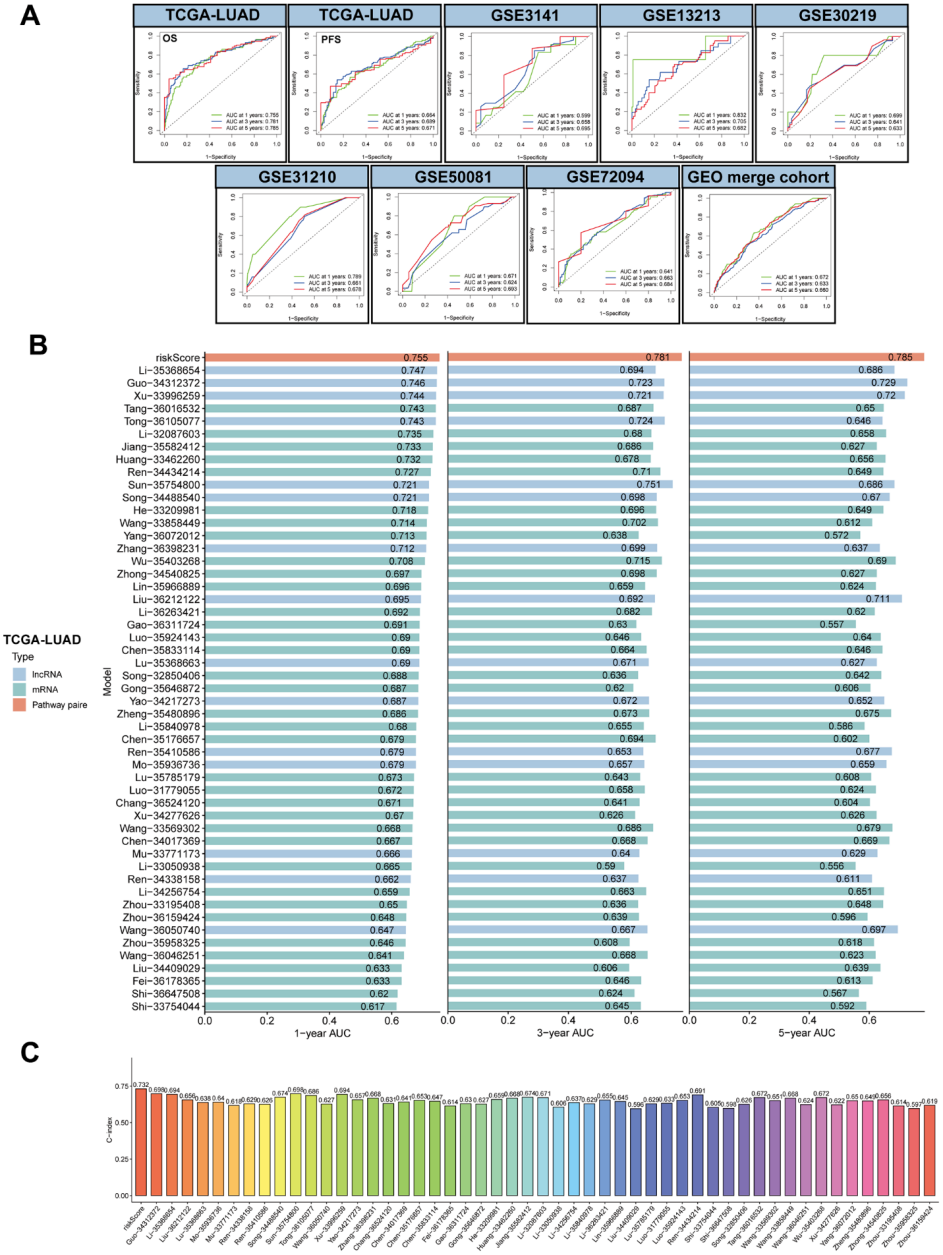
**Evaluation of MPPS model.** (**A**) The 1-, 3-, 5-year receiver operating characteristic (ROC) curves of MPPS model in TCGA-LUAD cohort and GEO validation cohorts. (**B**) The 1-, 3-, 5-year area under curves (AUC) of MPPS model and 51 published prognostic models of LUAD. (**C**) C-index of MPPS model and 51 published prognostic models of LUAD.

To investigate the accuracy of the MPPS model, ROC analysis was conducted and showed good performance in both training cohort and validation cohort (1-, 3-, 5-year AUC: 0.755, 0.781, 0.785 in OS of TCGA-LUAD; 0.664, 0.689, 0.671 in PFS of TCGA-LUAD; 0.599, 0.658, 0.695 in OS of GSE3141; 0.832, 0.705, 0.682 in OS of GSE13213; 0.699, 0.641, 0.633 in OS of GSE30219; 0.789, 0.661, 0.678 in OS of GSE31210; 0.671, 0.624, 0.693 in OS of GSE50081; 0.641, 0.663, 0.684 in OS of GSE72094; 0.672, 0.633, 0.66 in OS of GEO merge cohort) (Figure 4A). With the developments in next-generation sequencing, a considerable number of prognostic models were developed. To compare the performance of MPSS with other signatures, we retrieved 51 published signatures of LUAD including 15 lncRNA signatures and 36 mRNA signatures. These signatures encompassed various biological processes, such as autophagy, immune response, ferroptosis, stemness, epithelial-mesenchymal transition (EMT), hypoxia, ageing, methylation et al. The results suggested that MPSS had highest 1-, 3-, 5-year AUC of TCGA-LUAD cohort (Figure 4B). MPPS also displayed higher C-index in TCGA-LUAD cohort than almost all models (Figure 4C).

**Figure 5:**
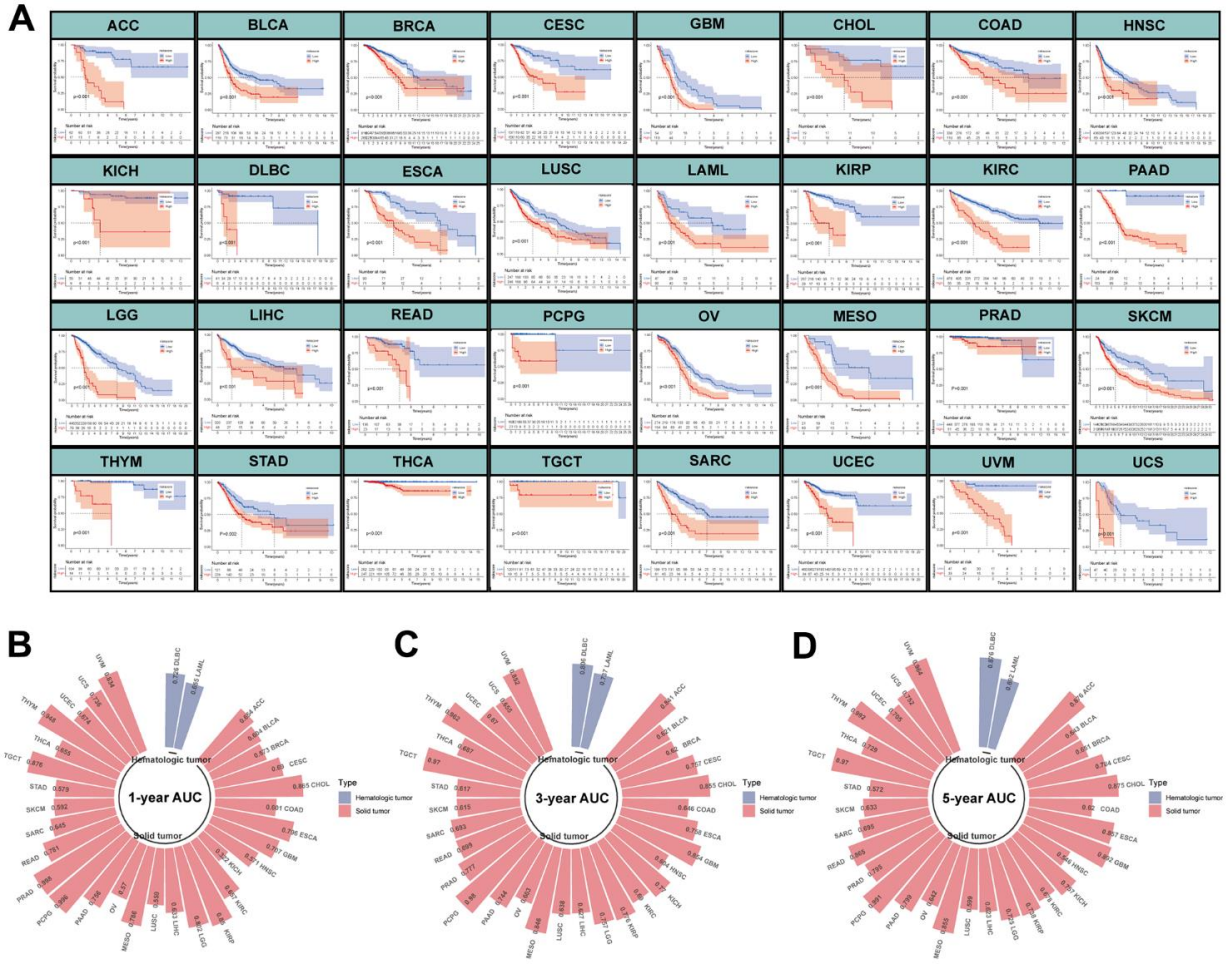
**Evaluation of MPPS model in pan-cancer.** (**A**) The Kaplan-Meier analysis of the high- and low-risk groups across 32 tumors in TCGA database except LUAD. The survival analysis was tested by log-rank test. (**B**–**D**) The 1-, 3-, 5-year AUC of MPPS model across 32 tumors in TCGA database except LUAD.

To further investigate the performance of MPPS on predicting prognosis of other tumors, we performed survival analyses of patients in the high- and low-MPPS groups involving 32 types of tumors in TCGA other than LUAD. Patients in the high-MPPS group had significantly worse OS than low-MPPS group in all 32 tumors (*P* < 0.05) (Figure 5A). MPPS also displayed high 1-, 3-, 5-year AUC in predicting prognosis of 32 tumors (Figure 5B–5D).

### Pathway enrichment and function annotation of the high- and low-MPPS groups

**Figure 6:**
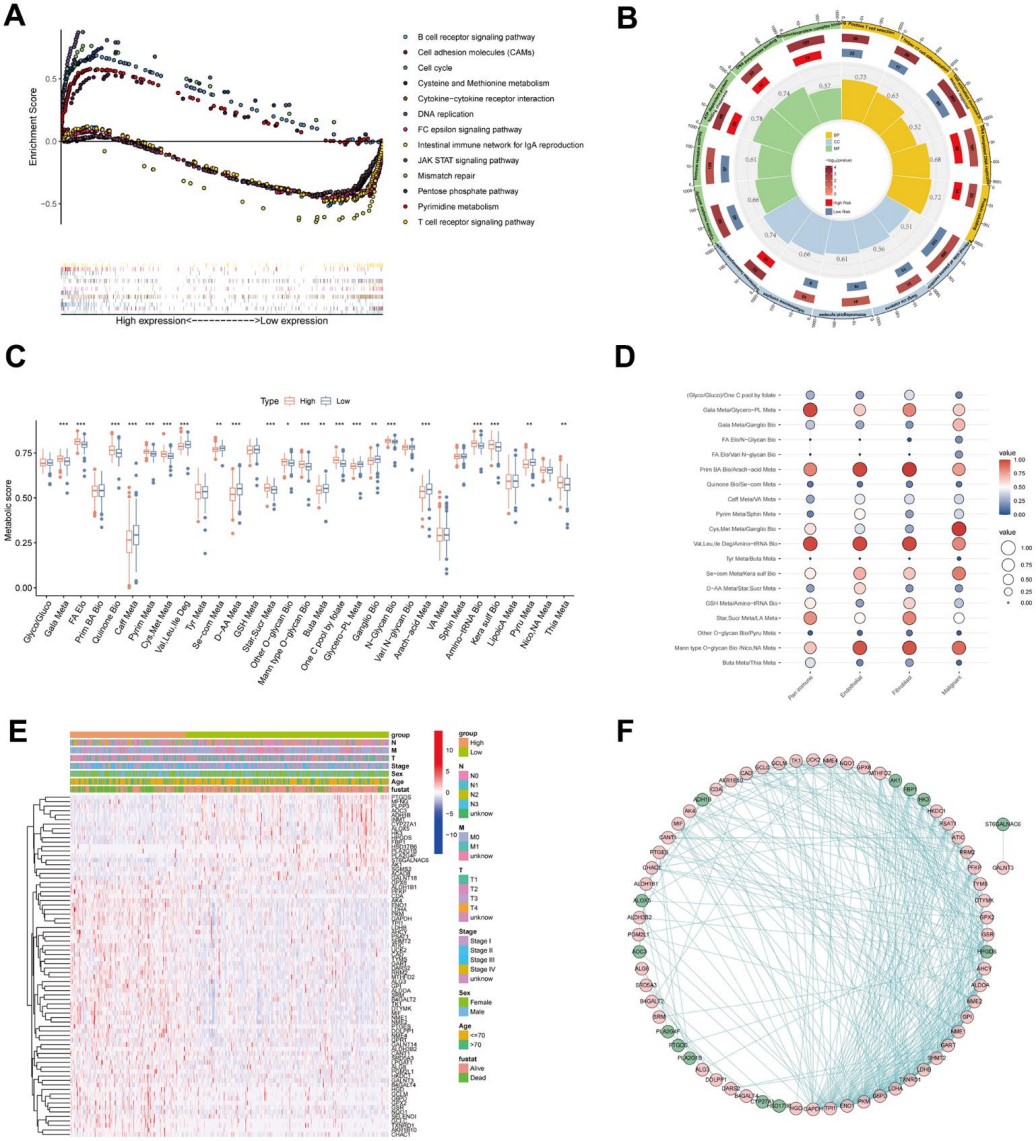
**Pathway enrichment and function annotation of the high- and low-risk groups.** (**A**) Kyoto Encyclopedia of Genes and Genomes (KEGG) pathway analyses between the high- and low-risk groups. (**B**) Gene Ontology (GO) between the high- and low-risk groups. (**C**) The differences of 31 metabolic pathways scores between the high- and low-risk groups. (**D**) The bubble diagram was drawn by the average of 19 metabolic pathway pairs in pan-immune cells, endothelial cells, fibroblasts, and malignant cells. (**E**) The differences of genes in 19 metabolic pathway pairs between the high- and low-risk groups. Pathological stage, sex, age, and survival status used as patients’ annotation. (**F**) The protein-protein interaction network by STRING website and the software Cytoscape v3.9.1. *P < 0.05, **P < 0.01, ***P < 0.001.

To explore the underlying mechanism of survival variation in different MPPS groups, we first performed pathway enrichment and function annotation of the high- and low-MPPS groups by KEGG and Gene Ontology (GO) analyses. The high-MPPS group had higher metabolic levels in pentose phosphate pathway, pyrimidine, cysteine and methionine metabolism. Multiple proliferation-related pathways including DNA replication, cell cycle, mismatch repair were enriched in the high-MPPS group (*P* < 0.05). On the contrary, immune response pathways such as B cell receptor signaling pathway, JAK/STAT signaling pathway, T cell receptor signaling pathway and cytokine-cytokine receptor interaction were more enriched in the low-MPPS group (Figure 6A). GO function annotation also demonstrated that DNA replication and translation were more associated with high MPPS, and immune cell development, maturation, activation and response were more related to low MPPS (*P* < 0.05) (Figure 6B). Additionally, multiple oncogenic pathways including hypoxia, epithelial-mesenchymal transition, DNA damage response, glycolysis, unfolded protein response and mTOC1 signaling et al. were significantly enriched in the high-MPPS group (Supplementary Figure 3A). The above results all indicated that LUAD with high MPPS possessed higher malignancy.

To characterize metabolic reprogramming involved in MPPS, we firstly analyzed the variation of 31 pathways in MPPS model between LUAD and normal lung tissues. A total of 26 pathways were dysregulated in LUAD, in which 7 metabolic pathways were up-regulated and 19 metabolic pathways were down-regulated (Supplementary Figure 3B). Further analyses revealed that 22 pathways were significantly variant between the high- and low-MPPS groups. The high-MPPS group had higher metabolic scores in the galactose metabolism, fatty acid (FA) elongation, pyrimidine metabolism, cysteine and methionine metabolism, one carbon pool by folate and aminoacyl-tRNA biosynthesis et al. indicating that biomass synthesis and proliferation were more active in the high-MPPS group. The low-MPPS group had higher metabolic scores in the caffeine metabolism, valine, leucine and isoleucine degradation, selenocompound metabolism and arachidonic acid metabolism, etc. (Figure 6C). The correlation of MPPS and 31 metabolic pathways was shown in Supplementary Figure 3C. By calculating the averages of 19 metabolic pathways pairs in each kind of TME cell, we found the lung malignant cells had higher level of cysteine and methionine metabolism/ganglio series biosynthesis than the immune cells, endothelial cells and fibroblasts (Figure 6D). The further differential expression analysis displayed that there existed obviously differential expression in 31 metabolic pathway genes between the high- and low-MPPS groups and the protein-protein interaction analysis demonstrated that the 76 DEGs had complex regulatory network (Figure 6E, 6F).

### Evaluation of TME and immunotherapeutic benefits in the high- and low-MPPS groups

**Figure 7:**
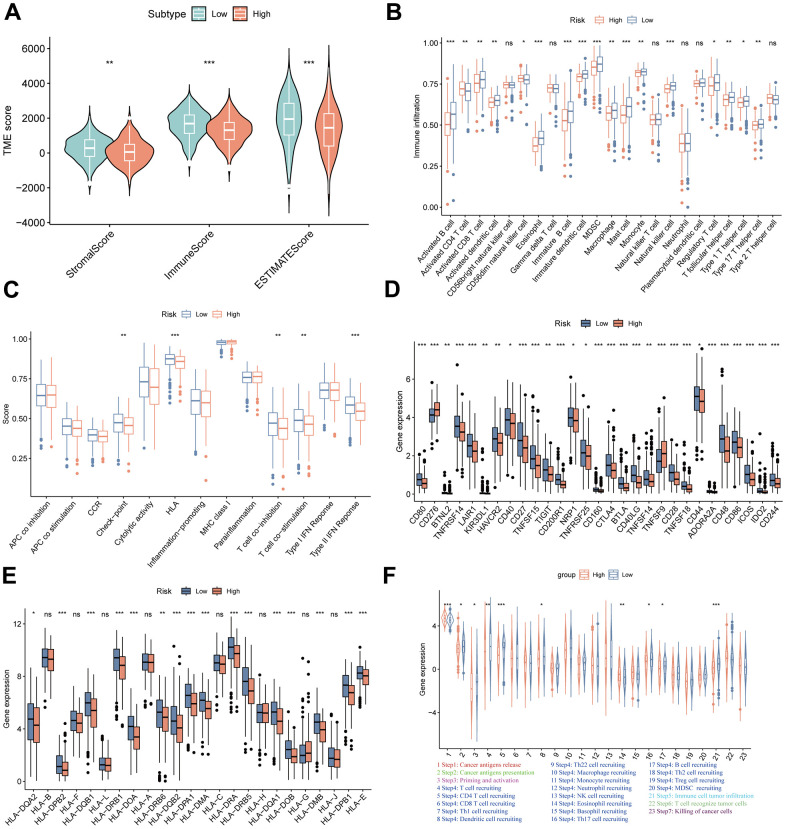
**TME landscapes of the high- and low-risk groups.** (**A**) The differences of stromal score, immune score and ESTIMATE score between the high- and low-risk groups. (**B**) The differences of infiltrating immune cells between the high- and low-risk groups by single sample gene set enrichment analysis (ssGSEA). (**C**) The differences of immune function between the high- and low-risk groups by ssGSEA. (**D**) The differences of immune checkpoint genes expression between the high- and low-risk groups. (**E**) The differences of HLA-related genes expression between the high- and low-risk groups. (**F**) The differences of cancer-immunity cycle scores between the high- and low-risk groups. *P < 0.05, **P < 0.01, ***P < 0.001.

Considering many immune-related pathways were enriched in the low-MPPS group, we evaluated the TME components between the high- and low-MPPS groups by estimate algorithm. The low-MPPS group had higher stromal score, immune score and ESTIMATE score than high-MPPS group (Figure 7A). The GSVA enrichment analysis was performed to evaluate immune filtrating cells and immunologic functions. The results showed that low-MPPS group had higher immune cells infiltration and immunologic functions activation in total including the infiltration of activated B cells, activated CD8+ T cells, activated dendritic cells, eosinophil and macrophage and the immune checkpoint, HLA, T cell co-inhibition or stimulation, type II IFN response (Figure 7B, 7C). A total of 29 immune checkpoint genes were differentially expressed between the high- and low-MPPS groups, in which 27 immune checkpoint genes, accounting for 93.1% were highly expressed in the low-MPPS group (Figure 7D). In addition, a total of 16 (66.7%) HLA genes expression were altered and they were all upregulated in the low-MPPS group (Figure 7E). The cancer–immunity cycle elucidates antitumor immune responses and offers an opportunity to understand the interactions between cancer and its immune system [23]. The low-MPPS group had higher cancer–immunity cycle scores in cancer antigens presentation, priming and activation, CD4^+^ T cell, dendritic cell, B cell, Th17 cell recruiting, and immune cells tumor infiltration but lower scores in cancer antigens release and eosinophil recruiting than the high-MPPS group (Figure 7F). To further investigate the correlation between MPPS and immunotherapy efficacy, we calculated the TIDE score. The results suggested that the low-MPPS group had higher T cell dysfunction score than the high-MPPS group (Supplementary Figure 4A). LUAD with high-MPPS score was more inclined to immune-desert or excluded phenotype and LUAD with low-MPPS score was more inclined to immune-inflamed phenotype (Supplementary Figure 4B). Higher immunophenoscore (IPS) was also exhibited by patients in the low-MPPS group compared with those in the high-MPPS group (Supplementary Figure 4C). The above results indicated that patients in the low-MPPS group may be more sensitive to immunotherapy.

**Figure 8:**
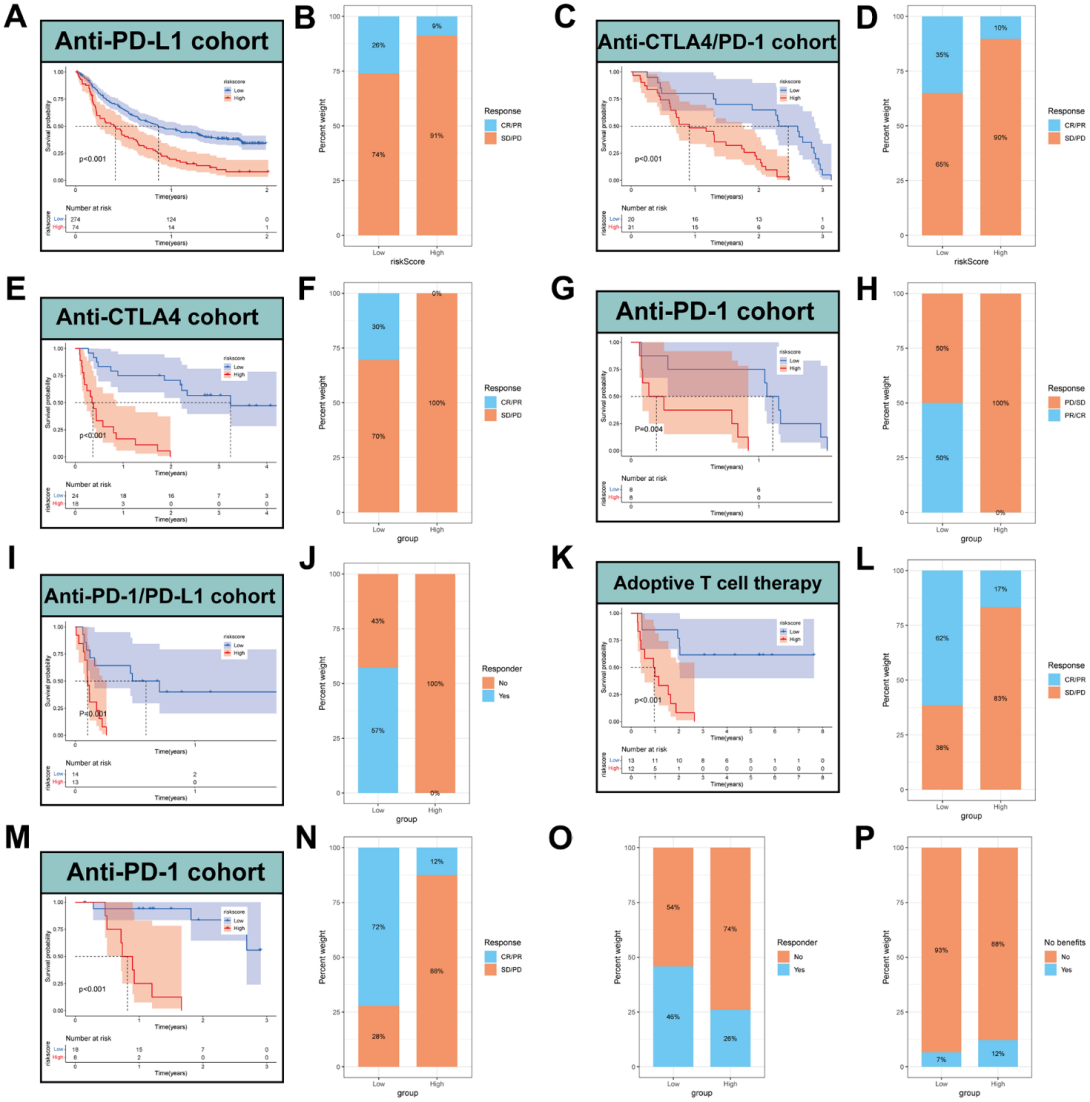
**Prediction of immunotherapy by MPPS model.** Survival analysis (**A**) and response to anti-PD-L1 therapy (**B**) between the high- and low-risk groups in advanced urothelial cancer (IMvigor210 cohort). Survival analysis (**C**) and response to anti-CTLA4 and anti-PD1 therapy (**D**) between the high- and low-risk groups in melanoma (GSE91061). Survival analysis (**E**) and response to anti-CTLA4 therapy (**F**) between the high- and low-risk groups in metastatic melanoma. Survival analysis (**G**) and response to anti-PD1 therapy (**H**) between the high- and low-risk groups in NSCLC (GSE126044). Survival analysis (**I**) and response to anti-PD-1/PD-L1 therapy (**J**) between the high- and low-risk groups in NSCLC (GSE135222). Survival analysis (**K**) and response to adoptive T cell therapy (**L**) between the high- and low-risk groups in melanoma. Survival analysis (**M**) and response to anti-PD-1 therapy (**N**) between the high- and low-risk groups in melanoma (GSE78220). (**O**) Difference of responder between low- and high-risk group of LUAD in TCGA. (**P**) Difference of benefits between low- and high-risk group of LUAD in TCGA.

To further validate the speculation, seven independent immunotherapy cohorts in the published literatures were used to validate immunotherapy efficacy and prognosis including advanced urothelial cancer treated with atezolizumab, an anti-PD-L1 antibody, melanoma treated with anti-CTLA4 and anti-PD-1 therapy, metastatic melanoma treated with anti-CTLA4 therapy, non-small cell lung cancer (NSCLC) treated with nivolumab or pembrolizumab, an anti-PD-1 antibody, NSCLC treated with anti-PD-1/PD-L1 antibody, melanoma treated with ACT, Melanoma treated with anti-PD-1 antibody. The results showed that the low-MPPS group had significant survival advantage and higher immune response rate compared to the high-MPPS group in all validation cohorts (Figure 8A–8N). The response to anti-PD-1 and anti-CTLA4 therapy was calculated using the TIDE website based on TCGA cohort. Patients in the low-MPPS group were more likely to be responders and benefit from immunotherapy (Figure 8O, 8P).

### TMB and drug sensitivity analysis

**Figure 9:**
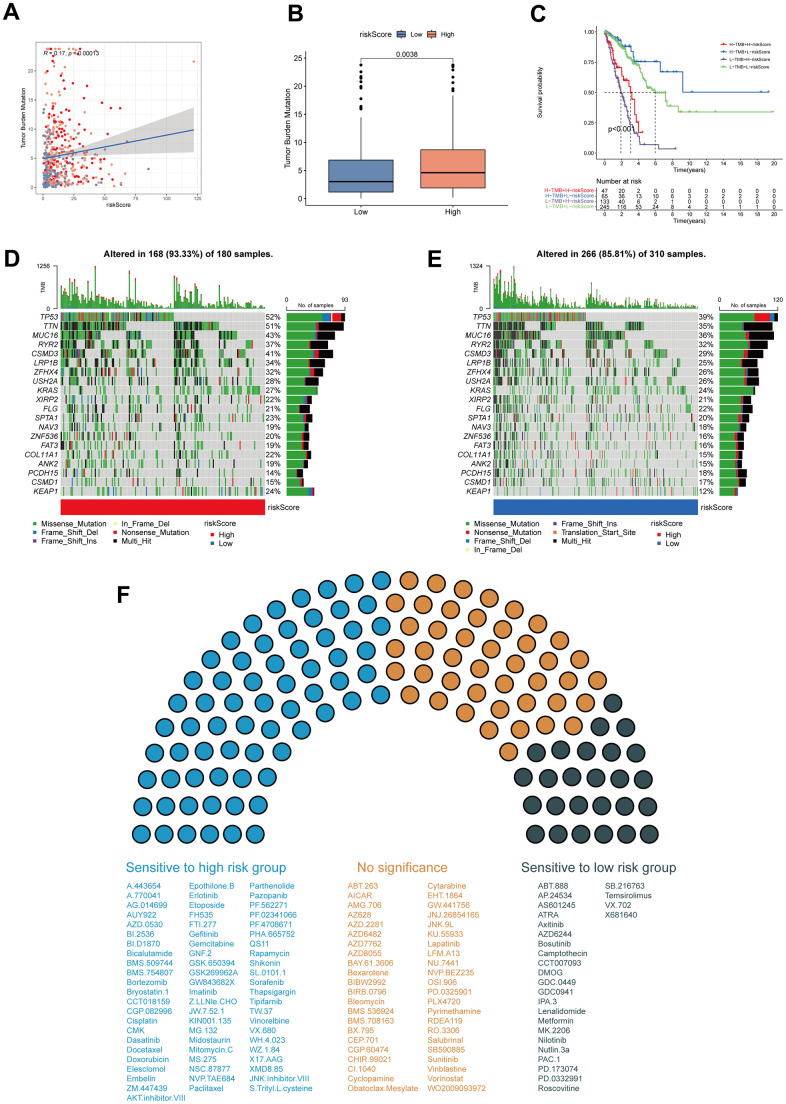
**Tumor mutation burden (TMB) and drug sensitivity analysis.** (**A**) The correlation of MPPS and TMB in TCGA-LUAD samples. (**B**) The differences of TMB between the high- and low-risk groups. (**C**) The Kaplan–Meier curves show OS differences stratified by TMB and MPPS. Visualization of the top 20 gene mutations in high-risk group (**D**) and low-risk group (**E**). (**F**) The sensitivity of 117 drugs between the high- and low-risk groups. *P* < 0.05 was considered as statistical significance.

To explore the correlation of MPPS and tumor mutation, Spearman correlation analysis was performed and significant positive correlation was found between MPPS and TMB (*R*=0.17, *P*=0.00013) (Figure 9A). LUAD patients in the high-MPPS group had higher TMB than those in the low-MPPS group (*P*=0.0038) (Figure 9B). By Kaplan-Meier analysis, we found LUAD patients with low-MPPS score and high TMB had the best survival advantages and LUAD patients with high-MPPS score and low TMB had the worst prognosis (*P*<0.001) (Figure 9C). The distribution of somatic mutations in the high- and low-MPPS groups was investigated in the TCGA-LUAD cohort. Patients in the high-MPPS group displayed significantly higher frequencies of somatic mutations compared with those in low-MPPS group (93.33% vs 85.81%), especially in TP53 (52% vs 39%), TTN (51% vs 35%), MUC16 (43% vs 36%), RYR2 (37% vs 32%), CSMD3 (41% vs 29%) and LRP1B (34% vs 25%). Moreover, missense mutation and multi-hit were the main mutation type in both high- and low-MPPS groups (Figure 9D, 9E). To further explore the clinical utility of MPPS in precision medicine, we assessed the sensitivity of 137 chemotherapeutic or targeted therapy drugs in different MPPS groups (Figure 9F). The results showed that the patients in the high-MPPS groups had lower IC50 values of 67 drugs, indicating sensitivity. Alternatively, the patients in the low-MPPS group were sensitive to 26 drugs. Together, the results may provide a standard of reference for treatment stratification of patients with LUAD.

### Establishment of a MPPS-based nomogram

**Figure 10:**
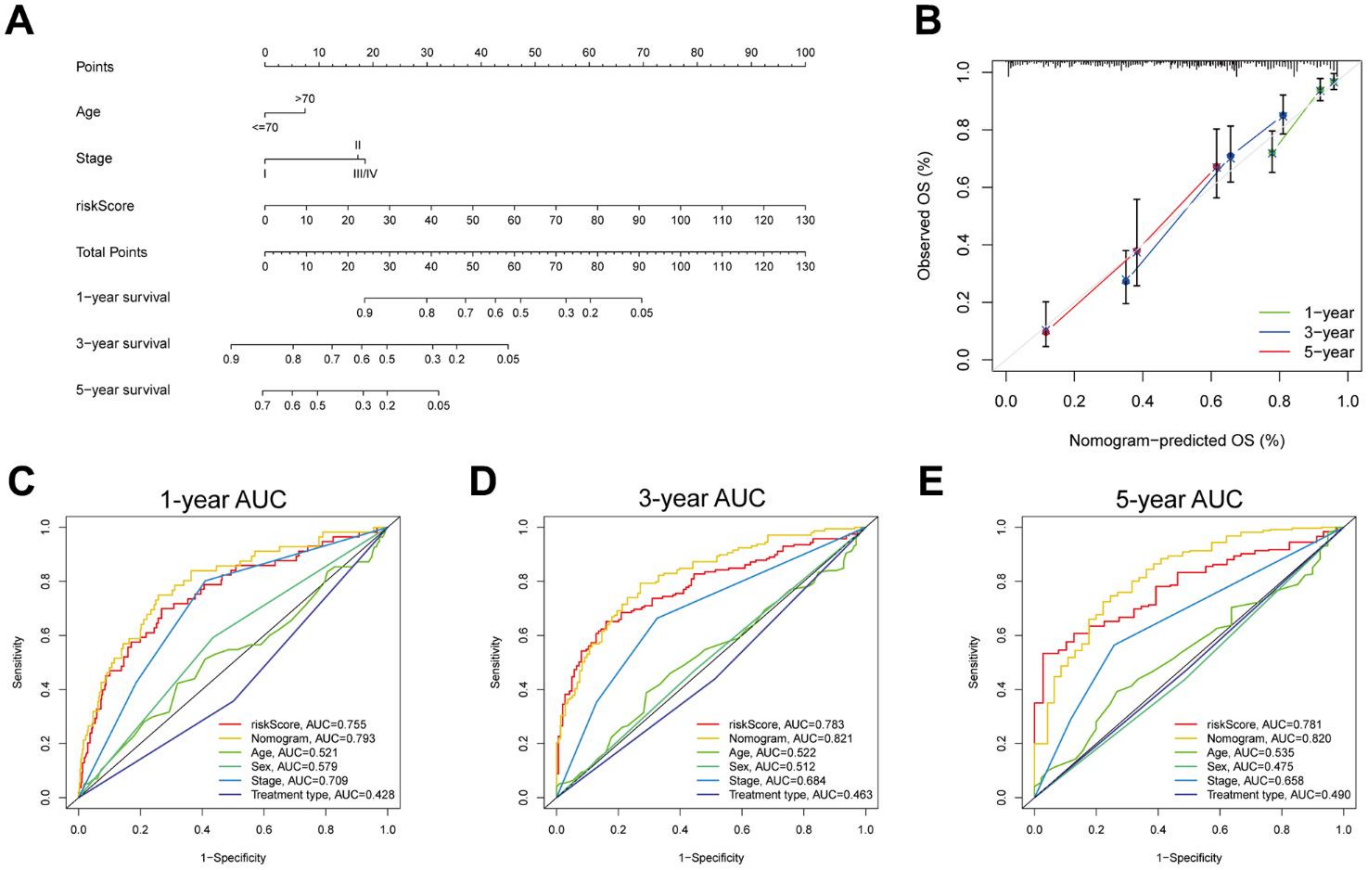
**Development and evaluation of a prognostic nomogram.** (**A**) Nomogram composed of MPPS, age, stage to predict 1-, 3-, 5-year OS probability. (**B**) Calibration curves of 1-, 3-, 5-year OS by nomogram. 1- (**C**), 3- (**D**), 5- (**E**) year ROC curves of MPPS, nomogram, age, sex, stage, and treatment type.

To construct a MPPS-based nomogram for convenient use, we analyzed the prognostic value of MPPS, age, sex, pathological stage and treatment type by univariable and multivariable Cox regression analyses (Table 1). In the univariable Cox regression analysis, age (HR (95%CI): 1.43 (1.056-1.936), *P*=0.021), stage (HR (95%CI): stage II 2.302 (1.590-3.332), *P*<0.001; stage III/IV 3.295 (2.309-4.702), *P*<0.001) and MPPS (HR (95%CI): 1.042 (1.036-1.049), *P*<0.001) were significantly related to LUAD prognosis. After adjusted by multivariable Cox regression analysis, MPPS, age and pathological stage were identified as independent prognostic factors and used to construct a prognostic nomogram (HR (95%CI): age 1.45 (1.065-1.975), *P* = 0.018; stage II 2.371 (1.637-3.434), *P* < 0.001; stage III/IV 2.537 (1.756-3.667), *P* < 0.001; MPPS 1.039 (1.032-1.046), *P* < 0.001). The prognostic nomogram made quantitative predictions of the 1-, 3-, and 5-year OS probabilities in patients with LUAD (Figure 10A). The calibration curves exhibited a high consistency between the predicted and actual 1-, 3-, and 5-year OS (Figure 10B). The ROC curves displayed the nomogram had higher AUC values than the single predictor such as MPPS, age, sex, stage, treatment type (1-, 3-, 5-year AUC: 0.793, 0.821, 0.82) (Figure 10C–10E).

**Table 1:** The results of univariable and multivariable Cox regression analyses.

**Characteristics**	**Univariable analysis**		**Multivariable analysis**	
	**HR (95%CI)**	***P*-value**	**HR (95%CI)**	***P*-value**
**Age**				
<=70	1		1	
>70	1.430 (1.056-1.936)	0.021	1.450 (1.065-1.975)	0.018
**Sex**				
Female	1			
Male	0.979 (0.726-1.320)	0.890		
**Stage**				
I	1		1	
II	2.302 (1.590-3.332)	<0.001	2.371 (1.637-3.434)	<0.001
III/IV	3.295 (2.309-4.702)	<0.001	2.537 (1.756-3.667)	<0.001
**Treatment type**				
Chemotherapy	1			
Radiotherapy	0.888 (0.658-1.199)	0.438		
**Risk score**				
Low	1		1	
High	1.042 (1.036-1.049)	<0.001	1.039 (1.032-1.046)	<0.001

### Identification of a MPPS-related gene signature by WGCNA and machine learning

**Figure 11:**
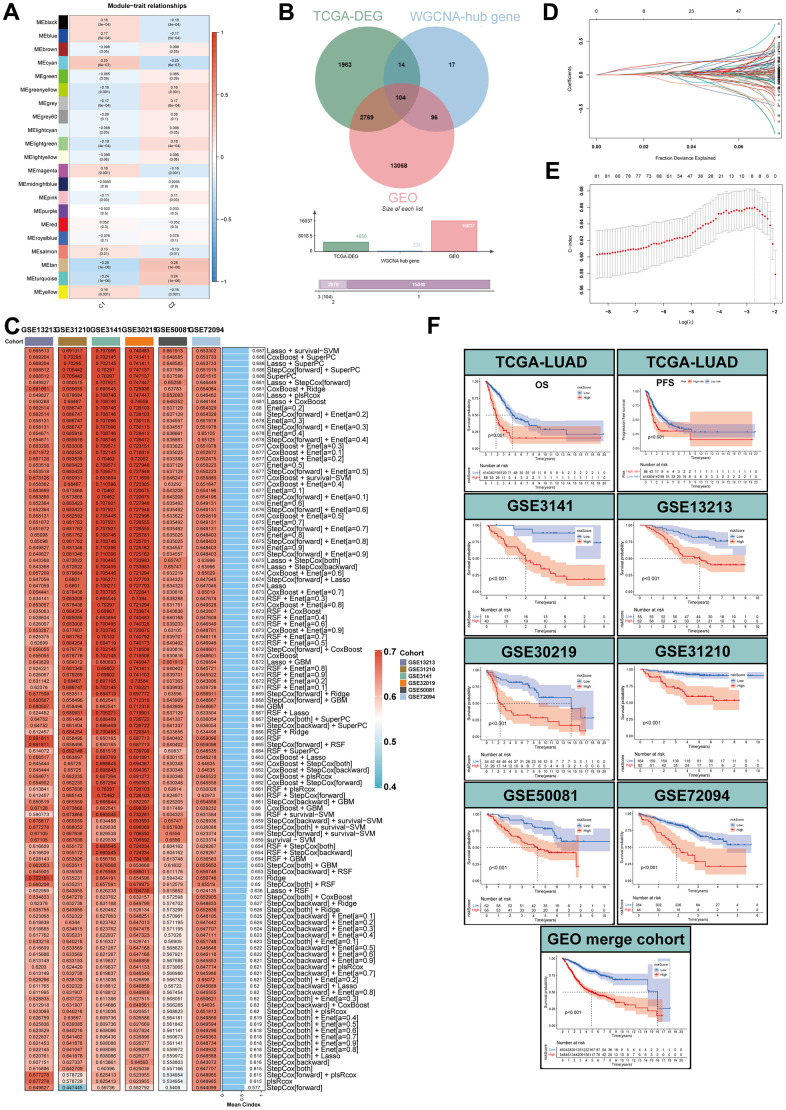
**Identification of MPPS-related genes by WGCNA and machine learning.** (**A**) Correlation analysis between module eigengenes and MPPS by WGCNA. (**B**) The intersection of WGCNA hub genes, TCGA-DEGs, and GEO genes. (**C**) The C-index of 117 machine learning algorithm combinations via LOOCV framework across all validation datasets. (**D**, **E**) Determination of the number of MPPS-related genes by the LASSO regression analysis. (**F**) The Kaplan-Meier analysis of the high- and low-gene risk scores groups stratified by LASSO and survival-SVM in the training and validation cohorts.

To identify MPPS-related modules, WGCNA analysis was performed and 21 modules were identified. 231 genes with gene significance (GS)>0.25, module membership (MM)>0.2 and *P* < 0.05 were considered as hub MPPS-related genes. Therefore, the hub genes in cyan, tan and turquoise modules met the criterion (Figure 11A). Intersecting with GEO genes from 6 GEO cohorts and DEGs between TCGA-LUAD and normal tissues, 104 hub MPPS-related genes were identified for subsequent analysis (Figure 11B). Based on the expression profiles of 104 hub MPPS-related genes, univariable Cox analysis identified 82 prognostic genes. These 82 genes were subjected to our machine learning-based integrative procedure to develop a consensus MPPS-related gene signature. In the TCGA-LUAD dataset, we fitted 117 kinds of prediction models via the LOOCV framework and further calculated the C-index of each model across 6 GEO validation datasets (Figure 11C). Finally, the 7-gene signature composed of ECT2, ANLN, SLC2A1, LDHA, GAPDH, C1QTNF6 and KRT8 identified by a combination of Lasso regression and survival-SVM had the highest mean C-index in the 6 validation cohorts (Figure 11D, 11E). A gene-based risk score for each patient was calculated by the survival-SVM algorithm and divided patients into the high- and low-risk group according to the optimal cutoff value determined by the “survminer” package. To validate the prognostic value of the gene signature, we performed Kaplan-Meier analysis. The patients in the high-risk group had significantly dismal OS and PFS compared to the low-risk group in the TCGA-LUAD training cohort and six GEO validation cohorts (all *P* < 0.05) (Figure 11F). The GEO merge cohort also showed the same trend (*P* < 0.05). In addition, ROC analysis measured the discrimination of the gene signature, with 1-, 3-, 5-year AUCs of 0.697, 0.704, 0.626 in OS of TCGA-LUAD; 0.643, 0.615, 0.556 in PFS of TCGA-LUAD; 0.763, 0.771, 0.686 in OS of GSE3141; 0.861, 0.676, 0.692 in OS of GSE13213; 0.673, 0.752, 0.777 in OS of GSE30219; 0.777, 0.727, 0.757 in OS of GSE31210; 0.758, 0.718, 0.698 in OS of GSE50081; 0.684, 0.643, 0.662 in OS of GSE72094; 0.716, 0.689, 0.699 in OS of GEO merge cohort (Supplementary Figure 5A). To further verify the predicting performance of the gene signature in the clinical practice, we next evaluated the mRNA expression of the 7 genes in a clinical cohort of 42 LUAD patients by qRT-PCR. The Kaplan-Meier analysis showed the low-risk group had better prognosis than the high-risk group (Supplementary Figure 5B). The model had high accuracy in predicting OS with 1-, 3-, 5-year AUCs of 0.763, 0.725, 0.762 in the clinical practice (Supplementary Figure 5C). Together, the MPPS-related gene signature had robust performance in prognostic prediction of LUAD.

### Expression, function, prognosis analyses of MPPS-related gene signature

**Figure 12:**
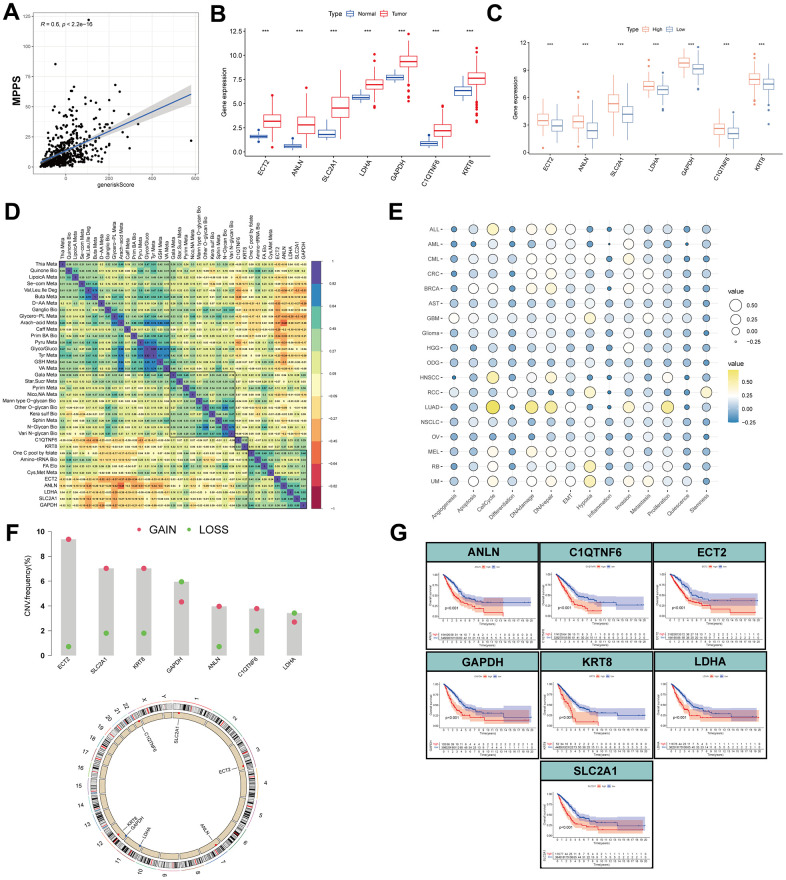
**Expression, function, prognosis analyses of MPPS-related gene signature.** (**A**) Correlation of MPPS and gene risk score by LASSO and survival-SVM. (**B**) The differences of the expression of 7 genes between TCGA-LUAD and normal samples. (**C**) The differences of the expression of 7 genes between the high- and low-MPPS groups. (**D**) The correlation of 7 genes expression and metabolic pathways. (**E**) The correlation of 7 genes expression and 14 biological processes by cancerSEA website. (**F**) The copy number variation frequency and location in chromosomes of the 7 genes. (**G**) The Kaplan-Meier analysis of the high- and low-expression of the 7 genes.

To investigate the correlation of MPPS and the gene risk score, Spearman correlation analysis was performed and significantly positive correlation was observed with *R*=0.6 and *P* < 2.2e-16 (Figure 12A). Next, the 7 genes all exhibited obviously higher expression in LUAD than normal lung tissue (Figure 12B). Compared to the low-MPPS group, the high-MPPS group had significantly upregulated expression (Figure 12C). To explore the correlation of 7 genes and 31 metabolic pathways, the correlation heatmap was drawn (Figure 12D). The result showed that there was a positive correlation among the 7 genes expression and their expression was positively associated with FA elongation, pyrimidine metabolism, cysteine and methionine metabolism and one carbon pool by folate and negatively associated with valine, leucine and isoleucine degradation, selenocompound metabolism, glycerophospholipid metabolism and arachidonic acid metabolism. Subsequently, we analyzed the correlation between the gene signature and 14 functional states across 18 cancers using CancerSEA data. The results manifested that the signature was positively related to LUAD proliferation, invasion, cell cycle, DNA damage and repair (Figure 12E). To investigate the mechanism underlying dysregulated expression, the CNV analysis was applied. The ECT2, SLC2A1, KRT8, ANLN and C1QTNF6 showed widespread CNV amplification. In contrast, GAPDH and LDHA had prevalent CNV depletion. The locations of CNV alterations of the 7 MPPS-related genes on chromosomes are shown in Figure 12F. Finally, the prognostic value of the 7 MPPS-related genes was analyzed by Kaplan-Meier curve in the TCGA-LUAD cohort (Figure 12G). The upregulation of the 7 genes all indicated worse survival (*P*<0.001).

### Effect of C1QTNF6 on infiltrating immune cells of TME

**Figure 13:**
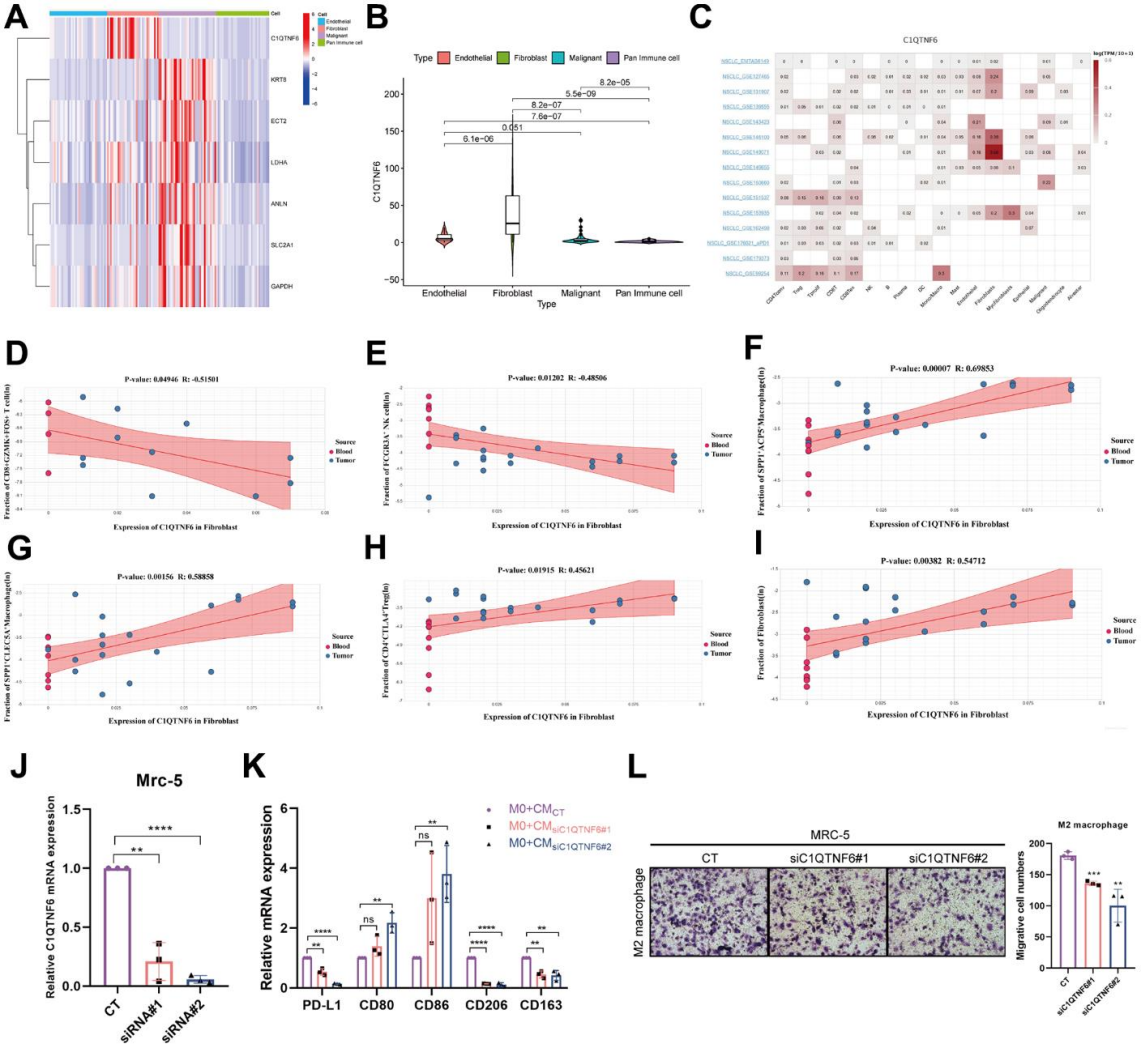
**Effect of C1QTNF6 on infiltrating immune cells of TME.** (**A**) The heatmap of 7 genes expression in different cells by scRNA-seq. (**B**) The differences of C1QTNF6 expression among pan-immune cells, endothelial cells, fibroblasts and malignant cells. (**C**) The heatmap of C1QTNF6 expression in TME cells by multiple scRNA-seq datasets. The correlation of C1QTNF6 expression in fibroblasts and immune cells infiltration (memory CD8^+^ T cell (**D**), NK cell (**E**), M2 macrophages (**F**, **G**), Treg cell (**H**), fibroblast (**I**)). (**J**) qRT-PCR was performed to detect the efficiency of C1QTNF6-siRNA transfection. (**K**) M0 macrophages were stimulated by conditional medium from MRC-5 cells with C1QTNF6 silencing for 48h. qRT-PCR was performed to detect the expression of PD-L1, M1 and M2 markers. (**L**) Representatives and summary of M2 macrophage migration assays induced with MRC-5 cells with or without C1QTNF6 silencing. The data were presented as the mean±SD; n = 3. *P < 0.05, **P < 0.01, ***P < 0.001.

By investigating MPPS-related genes expression in specific cells in TME, we found only C1QTNF6 was highly expressed in fibroblasts and the other six genes were mainly expressed in malignant cells (Figure 13A). Moreover, referring to the published literatures, little is known about the function of C1QTNF6 compared to the other six genes. Consequently, we focused on the function of C1QTNF6. The violin plot showed that the expression of C1QTNF6 was the highest in fibroblasts, followed by endothelial cells, malignant cells and pan-immune cells (Figure 13B). By comparing multiple single-cell datasets, the similar expression trend was obtained (Figure 13C). Next, we analyzed the effect of C1QTNF6 expression in fibroblasts on infiltrating immune cells of TME by scTIME Portal website. GZMK^+^FOS^+^CD8^+^ T cells have been identified as memory T cells and are prevalent in para-carcinoma tissues or normal donors. FCGR3A^+^ NK cell is a classic NK cell cluster and plays important roles in anti-tumor immunity. C1QTNF6 expression in fibroblasts was dramatically negative relation to the infiltration of GZMK^+^FOS^+^CD8^+^ T cells (*R*=-0.515, *P*=0.049) and FCGR3A^+^ NK cell (*R*=-0.485, *P*=0.012) (Figure 13D, 13E). SPP1^+^ACP5^+^ macrophage and SPP1^+^CLEC5A^+^ macrophage are biased toward an M2 signature. Their infiltrating abundance was significantly positive to C1QTNF6 expression in fibroblasts (SPP1^+^ACP5^+^ macrophage: *R*=0.699, *P*<0.001; SPP1^+^CLEC5A^+^ macrophage: *R*=0.589, *P*<0.01) (Figure 13F, 13G). CTLA4^+^CD4^+^ Tregs are enriched in tumors and commonly involved in T cell inhibition across patients and tumor types. C1QTNF6 expression in fibroblasts was positive related to CTLA4^+^CD4^+^ Tregs abundance in TME, indicating high C1QTNF6 expression in fibroblasts might result in more CTLA4^+^CD4^+^ Tregs infiltration in TME (*R*=0.456, *P*=0.019) (Figure 13H). Interestingly, C1QTNF6 expression in fibroblasts was also positively associated with fibroblast infiltration in TME (*R*=0.547, *P*<0.01) (Figure 13I). Subsequently, we analyzed the cell communication between fibroblasts and immune cells. The results suggested that fibroblasts had strong interactions with M2 macrophages including C1QC^+^PLTP^+^ macrophage, SPP1^+^ACP5^+^ macrophage, SPP1^+^CLEC5^+^ macrophage (Supplementary Figure 6A). Furthermore, the ligand-receptor interaction analysis suggested that fibroblasts were very likely to interact with M2 macrophage through CD74-COPA, CD74-APP and CD74-MIF (Supplementary Figure 6B–6D). By analyzing the hallmarks enrichment between high and low C1QTNF6 expression groups, we found multiple pathways related to M2 polarization were enriched in high C1QTNF6 expression groups including NF-κB signaling pathway, glycolysis, IL6/JAK/STAT3 signaling pathway, TGF-β signaling pathway, Wnt/β-catenin signaling pathway, and Hedgehog signaling pathway (Supplementary Figure 6E). Consequently, we hypothesized that C1QTNF6 expression in fibroblasts may affect M2 macrophage polarization or recruitment.

To validate the hypothesis, we constructed MRC-5 cells with C1QTNF6 silencing by siRNAs. As shown in Figure 13J, the specific cells were successfully established with high silencing efficiency. After 48h, the supernatant was collected, centrifuged and prepared as CM. To detect the effects of silencing C1QTNF6 in MRC-5 cells on macrophages polarization, we cultured M0 macrophages with the mixture of CM and FBS-containing medium (1:1) for 48h. Compared to the control, the group with C1QTNF6 silencing had significantly decreased PD-L1 and M2 macrophage-related genes expression (CD163, CD206). Conversely, M1 macrophage-related genes expression (CD80, CD86) were obviously increased when C1QTNF6 was silenced in MRC-5 (Figure 13K). Furthermore, we successfully induced M0 to M2 macrophage by IL-4 and IL-13 stimulation (Supplementary Figure 6F). The macrophage migration assay showed that silencing C1QTNF6 in MRC-5 cells could reduce M2 macrophage migration *in vitro* (Figure 13L). These fundings suggested that C1QTNF6 expression in MRC-5 cells promoted M2 macrophage polarization and recruitment.

### Cause effect of C1QTNF6 on lung cancer onset

**Figure 14:**
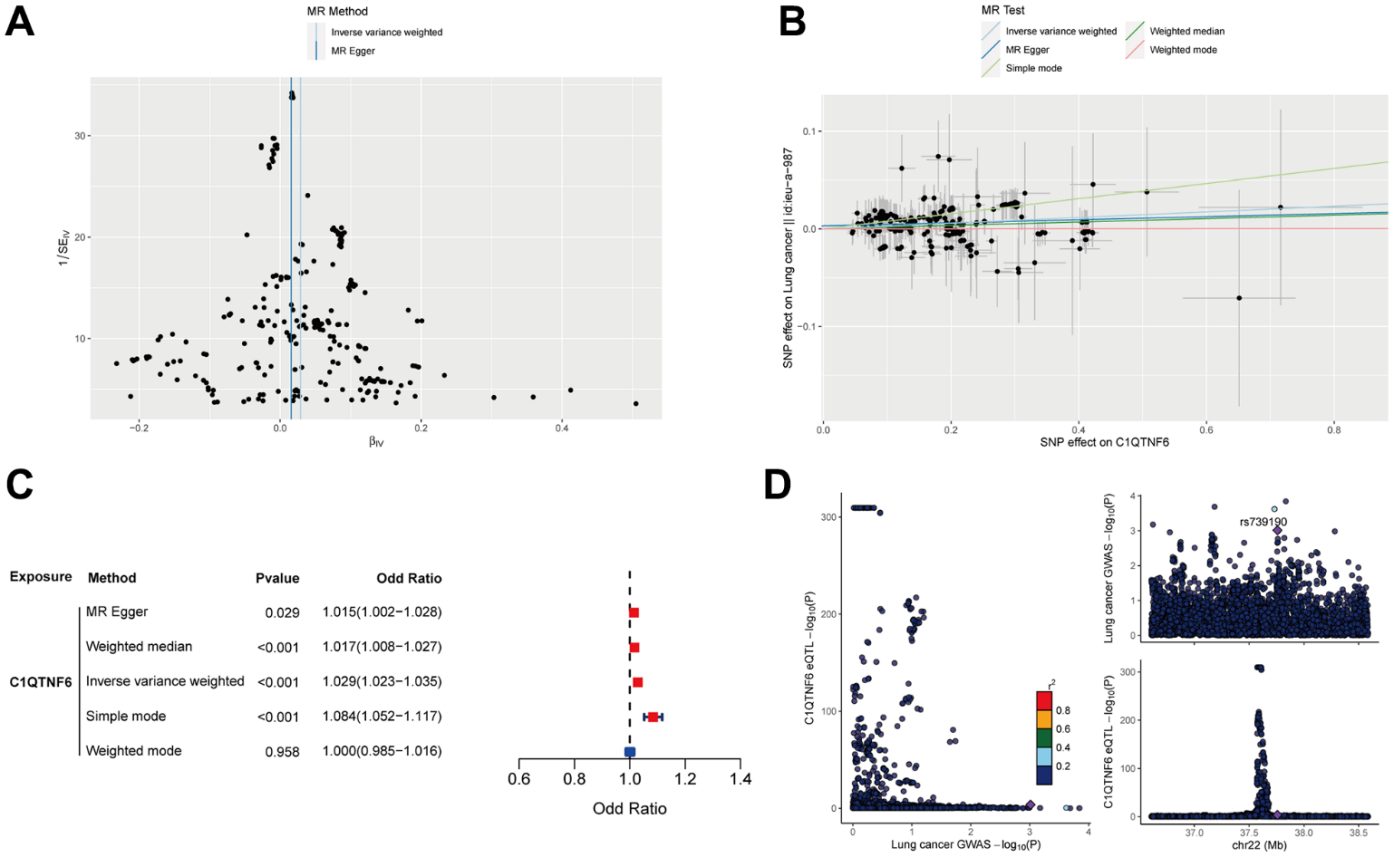
**Mendelian randomization analysis of C1QTNF6 and lung cancer.** (**A**) The funnel plot displayed the distribution of instrumental variables for C1QTNF6. (**B**) Scatter plot showed that C1QTNF6 increased the risk of lung cancer. (**C**) Forest plot showed the cause effect of C1QTNF6 on lung cancer onset. (**D**) The co-localization analysis of C1QTNF6 and lung cancer.

To evaluate the cause effect of C1QTNF6 on lung cancer onset, MR analysis was performed. 340 eligible SNPs were used as instrumental variables for C1QTNF6 and 274 common SNPs were obtained after harmonization. The funnel plot and leave-one-out sensitivity analysis showed that there were no obviously heterogeneous SNPs (Figure 14A and Supplementary Figure 7). MR analysis revealed that C1QTNF6 expression increased the risk of lung cancer (Figure 14B). Except weighted mode, the other four methods all showed the same trend (OR (95%CI): Inverse variance weighting (IVW) 1.029 (1.023-1.035) *P* < 0.001; MR Egger 1.015 (1.002-1.028) *P* =0.029; Weighted median 1.017 (1.008-1.027) *P* < 0.001; Simple mode 1.084 (1.052-1.117) *P* < 0.001) (Figure 14C). there was no heterogeneity and horizontal pleiotropy (the Cochrane’s Q-value > 0.1; MR PRESSO global test *P* = 0.236), indicated that the result of MR analysis was credible. Moreover, Steiger filtering further ensured directionality with all *P*-values of SNP less than 0.05. However, Bayesian co-localization showed that there was no genetic co-localization between C1QTNF6 and lung cancer (PP.H4=4.59e-03) (Figure 14D).

## DISCUSSION

LUAD is a highly aggressive malignancy with an unfavorable prognosis and average 5-year survival rate of only 15% [24]. With the rapid development of immunotherapy, it has shown great potential in the treatment of cancer. However, only about one third of patients can benefit from immunotherapy due to heterogeneity and adaptive evolution of tumor cells [4]. To advance precision medicine, it is necessary to stratify cancer patients into distinct groups according to their prognosis and immunotherapy response before treatment. With the advances in sequencing technology, more and more gene expression-based prognostic models have been constructed to predict the prognosis and immunotherapy response of cancer patients [21, 25, 26]. Unfortunately, the most models have not robust performance in other cohorts due to sequencing data from different platforms. To overcome this obstacle, pairing multiple markers to construct a prognostic model was put forward creatively. Metabolic reprogramming has been identified as a new hallmark of cancer and tightly associated with clinical outcomes and immunotherapy efficacy. Tumor cells reprogram their metabolism to compete for nutrients with other cells in TME, deal with oxidative stress, and reshape an immunosuppressive TME to evade the immune system [27, 28]. Comprehensively depicting the metabolic profile of LUAD is promising to predict the survival and immunotherapy efficacy of LUAD patients.

In this study, we assessed 84 metabolic pathways involved in 12 kinds of metabolism in LUAD by ssGSEA and analyzed the metabolic heterogeneity of LUAD. Then, we paired the 84 pathways and identified 19 metabolic pathway pairs by univariable, LASSO, multivariable Cox regression analysis. Using the 19 metabolic pathway pairs, we established a MPPS system and stratified LUAD patients into the high- and low-MPPS group. The high-MPPS group was characterized by high galactose metabolism, FA elongation, pyrimidine metabolism, cysteine and methionine metabolism, one carbon pool by folate and aminoacyl-tRNA biosynthesis. The low-MPPS group was characterized by dominant caffeine metabolism, valine, leucine and isoleucine degradation, selenocompound metabolism and arachidonic acid metabolism. Galactose is another important carbohydrate and involved in glycosylation, energy storage and pentose phosphate pathway directly or indirectly [29]. Many tumors preferentially use glycolysis for survival and proliferation and have metabolic vulnerability to galactose. It has been reported that tumor cells with Akt activation will be induced cell death in galactose culture [30]. Thus, LUAD with high galactose metabolism may be more adaptative for various energy substances. FA biosynthesis includes de novo synthesis and FA elongation. Elongation of very long-chain fatty acid (ELOVL) family enzymes are responsible for catalyzing FA elongation. Disruption of FA elongation by silencing ELOVL5 can suppress proliferation and invasion of renal cell carcinoma [31]. Moreover, VLCFA deficiency results in a marked decrease in ceramides as well as downstream glucosylceramides and sphingomyelins, which impairs mitochondrial morphology and renders cancer cells sensitive to oxidative stress and cell death [32]. Pyrimidine metabolism, one carbon pool by folate and aminoacyl-tRNA biosynthesis are tightly associated to nucleotides biosynthesis and translation, which are indispensable for rapid proliferation of malignant cells. UBE2T-mediated Akt K63 ubiquitination and Akt/β-catenin activation accelerate hepatocellular carcinoma development by increasing pyrimidine metabolism [33]. Combination of pyrimidine synthesis inhibitors and other anti-tumor drugs is promising to kill tumor cells [34]. Nucleotide synthesis and DNA methylation are highly dependent on one carbon pool by folate, which supports vital events for growth and survival [35]. Methionine and cysteine, two of the most representative sulfur amino acids, play a crucial role in protein structure, metabolism, immunity, and especially, oxidation. They are extremely sensitive to almost all forms of reactive oxygen species and protect cells from oxidative stress damage [36]. Dietary restriction of methionine and cysteine will alter the energetic metabolism and enhance the sensitivity of gliomas to ferroptosis [37]. These metabolic pathways are highly elevated in high-MPPS group and may shape a refractory phenotype. Conversely, many anti-tumor metabolic pathways were elevated in the low-MPPS group. Caffeine can enhance anti-tumor activity of anti-PD-1 monoclonal antibody by increasing the infiltration of CD4^+^ and CD8^+^ T lymphocytes and decreasing the infiltration of Treg cells [38]. The branched-chain amino acids (BCAAs) (valine, leucine, and isoleucine) are essential amino acids that play important roles in metabolic regulation. The accumulation of BCAAs can activate mTOR signaling pathway to promote tumor proliferation [39]. Thus, degradation of BCAAs may be harmful to tumor progression. Se compounds have been demonstrated as anticancer agents *in vivo* and *in vitro* experiments. They can prevent oncogene activation and cancer cell differentiation through scavenging of ROS, tumor-promoting eicosanoids and inducing tumor suppressor genes expression [40]. Arachidonic acid metabolism is a double-edged sword in tumor initiation and progression. On the one hand, arachidonic acid can inhibit M2 macrophage polarization and enhance ferroptosis sensitivity to suppress tumor progression [41, 42]. On the other hand, it promotes stromal cell-mediated immunosuppression in NSCLC [43]. Consequently, the MPPS system divided LUAD patients into distinct metabolic reprogramming subgroups well.

The MPPS system displayed robust performance on recognizing LAUD patients’ prognosis whether in training cohort or validation cohorts. The high-MPPS group had a worse prognosis than the low-MPPS group. ROC curves showed the model had high accuracy on prediction of prognosis. Comparing to the other published models and several clinical features (age, sex, stage and treatment type), the MPPS model had significantly improved accuracy. Moreover, the model was also applicable in the other 32 tumors. These results suggest that the MPPS model is promising to be applied in the clinical practice.

Increasing evidence demonstrates that metabolic reprogramming in TME affects anti-tumor immunity. For example, targeting glutamine metabolism increased antitumor immunity in mouse models by upregulating mitochondrial metabolism of cytotoxic T lymphocytes in NSCLC [44, 45]. Treg cells rely on oxidative phosphorylation and FA oxidation to support their survival and differentiation [46]. Lipid metabolic reprogramming can prevent effector T cells senescence and enhance immunotherapy efficacy [47]. These also reveal that deeply understanding and depicting metabolic heterogeneity can favor immunotherapy. However, up to now, there is still a lack of comprehensive depiction of heterogeneous metabolic landscape in TME. The evaluation of 84 metabolic pathways in LUAD revealed the metabolic heterogeneity of LUAD in this study. Considering the tight association of metabolism and immunotherapy, we wondered whether the LUAD patients with different MPPS had different responses to immunotherapy. Using seven independent immunotherapy cohorts, we found that the patients with low-MPPS scores commonly had higher immunotherapy response rates than those with high-MPPS scores. To further explore the alteration of immune cells, molecules and function, it was revealed that more immune cells infiltration, immune-related genes expression, and immune function activation were in the low-MPPS group, such as activated B cells, activated CD8^+^ T cells, activated dendritic cells, eosinophil and macrophage and the immune checkpoint, HLA, T cell co-inhibition or stimulation, type II IFN response. The low-MPPS group also had higher cancer–immunity cycle scores in cancer antigen presentation, priming and activation, CD4^+^ T cell, dendritic cell, B cell, Th17 cell recruiting, and immune cells tumor infiltration. These results implied that LUAD with the low-MPPS score was inclined to be a “hot” TME and LUAD with the high-MPPS score was a “cold” TME. TIDE score also validated the conclusion and T cell dysfunction was higher in the low-MPPS group than the high-MPPS group. With the increase of MPPS, the inflamed TME was transformed to the excluded TME. TMB is emerging as another indicator of immunotherapy except for PD-L1 expression. The high-MPPS group had higher TMB compared to the low-MPPS group. The LUAD patients with high TMB and low MPPS had the best prognosis and those with low TMB and high MPPS had the worst prognosis. Consequently, the bad prognosis of the high-MPPS group is not likely due to TMB. By identifying the sensitivity of 137 chemotherapy drugs, multiple drugs sensitive to the high- or low-MPPS group were determined, which may be helpful to guide precision medicine of LUAD patients.

Although targeting cancer metabolism to improve immunotherapy efficacy is highly promising, the crosstalk of metabolic pathways between tumor cells and immune cells in TME lead to disruption of normal metabolic pathways in immune cells by strategies to inhibit/alter cancer metabolism [48]. Thus, it is critical to target the specific metabolic pathways to kill tumors without interfering with or even promoting anti-tumor immunity. To identify such pathways, we analyzed the metabolic pathway pair in different kinds of cells by scRNA-seq data. Interestingly, the average value of cysteine and methionine metabolism/ganglio series biosynthesis is significantly elevated in malignant cells than the other cells including immune cells, fibroblasts, endothelial cells. Many previous studies have reported that tumor cells are highly dependent on cysteine and methionine metabolism than normal cells and they are promising targetable weaknesses of cancer cells [49]. Ganglio series biosynthesis are also tightly related to some malignant phenotypes such as metastasis [50]. As a result, this metabolic pathway pair may be promising to be a metabolic target in LUAD therapy.

The previous studies mostly choose the modeling algorithms to identify the hub genes based on their knowledge limitations and preferences. To overcome this shortcoming, we firstly identified the MPPS-related hub gene module by WGCNA and then, integrated 117 machine learning algorithms to further recognize the prognostic signature. Finally, seven genes were identified, in which C1QTNF6 caught our attention due to its specific expression in fibroblast. Some studies have suggested that silencing C1QTNF6 in LUAD cells can suppress the proliferation, migration and invasion of LUAD cells [51]. C1QTNF6 is a prognostic indicator for poor survival across many cancers including LUAD and one of the most relative genes of TAM [52, 53]. However, there is still little knowledge about the function of C1QTNF6 in tumors.

By analyzing multiple scRNA-seq datasets, we found C1QTNF6 expression was mainly focused on fibroblast and its expression in fibroblast was positively related to the infiltration of M2 macrophages, Treg cells, and negatively related to the infiltration of memory CD8^+^ T cells, NK cells. Moreover, there existed strong interaction between M2 macrophages and fibroblast by intercellular communication analysis. *In vitro* experiments also validated that the CM from fibroblast*^C1QTNF6-/-^* would promote the transformation of M0 into M1 but not M2 macrophage, decrease PD-L1 expression, and reduce M2 macrophage migration. Hallmarks enrichment analysis showed that NF-κB signaling pathway, glycolysis, IL6/JAK/STAT3 signaling pathway, TGF-β signaling pathway, Wnt/β-catenin signaling pathway, and Hedgehog signaling pathway were enriched in high C1QTNF6 expression group, which were reported to participate in M2 macrophage polarization. Inhibition of autophagic degradation of RELA will rescue activity of NF-κB signaling pathway and shape the phenotype of hepatoma-polarized M2 macrophages [54]. Activation of IL6/JAK/STAT3 signaling pathway in macrophages can promote M2 polarization and PD-L1 expression [55, 56]. A large amount of lactate produced by glycolysis induces M2 macrophage polarization and promotes the invasion of pituitary adenoma [57]. Mesenchymal stem cells can induce M2 polarization phenotype via secreting TGF-β to activate Akt/FoxO1 pathway in LPS-stimulated macrophages [58]. It is also reported that crosstalk between hepatic tumor cells and macrophages by Wnt/β-catenin signaling pathway can promote M2 polarization [59]. FOXM1 can induce M2 polarization through **S**EMA3C/NRP2/Hedgehog signaling [60]. The results indicated that C1QTNF6 may be tightly associated with M2 polarization. Lin et al. reported that after silencing C1QTNF6, the enrichment of cytokine-cytokine receptor interaction pathways was reduced in LUAD cell by RNA sequencing, which indicated that C1QTNF6 may participate in cytokine-cytokine receptor interaction pathways directly or indirectly [61]. Consequently, C1QTNF6 expression in fibroblast may promote M2 macrophage polarization and migration by regulating cytokine-cytokine receptor interaction.

Mendelian randomization (MR) is as a valuable tool for inferring causal relationships between exposure and outcome by leveraging Genome wide association study (GWAS) data. The result of MR suggested that C1QTNF6 expression had the increased risk of lung cancer although there was no evidence of co-localization. The MR result was consistent with the expression and prognosis of C1QTNF6 in LUAD.

There are still some limitations in our study. Although we identified two distinct metabolic subtypes with significantly different prognosis and immunotherapy efficacy, some immunotherapy cohorts were from the studies about urothelial cancer or melanoma and more LUAD-related immunotherapy cohorts are needed to validate our conclusion. The drug sensitivity needs further validation by IC50 assays. Although we identified the potential metabolic pathways associated with prognosis, the targetable molecules for the pathways remain to be explored. The underlying mechanism that C1QTNF6 regulated M2 macrophage polarization and migration remains to be elucidated. Moreover, the relationships of C1QTNF6 and the other immune cells need further exploration. The conclusion of MR needs further experimental validation. The above insufficient will be the focus of our future study.

## CONCLUSIONS

Based on 84 metabolic pathways, we constructed a MPPS model to accurately predict the prognosis and immunotherapy efficacy of LUAD patients. Targeting C1QTNF6, a MPPS-related gene, is promising to suppress M2 macrophage polarization and migration.

## MATERIALS AND METHODS

### Data collection and processing

Gene expression data of LUAD and corresponding clinical characteristics were respectively retrieved from The Cancer Genome Atlas (TCGA) (HTseq-fragments per kilobase million, HTseq-FPKM) (https://cancergenome.nih.gov/) and Gene-Expression Omnibus (GEO) (https://www.ncbi.nlm.nih.gov/geo/) databases. Patients without prognostic information or survival time = 0 were excluded. Then 500 LUAD cases from TCGA database and 1009 LUAD cases from GEO database were retrieved as the training cohort and validation cohort (GSE3141: 58 cases; GSE13213: 117 cases; GSE30219: 83 cases; GSE31210: 226 cases; GSE50081: 127 cases; GSE72094: 398 cases). The demographic was shown in Supplementary Table 1. The ComBat method from the ‘SVA’ R package was used to remove the batch effects among different GEO datasets. Pan-cancer mRNA expression profiles and prognostic information were obtained from UCSC Xena website (https://xenabrowser.net/datapages/). The somatic mutation and copy number variation (CNV) of TCGA-LUAD were also curated from TCGA database. Eighty-four metabolic pathway gene sets were extracted from the KEGG database. The abbreviations of 84 metabolic pathways were listed in Supplementary Table 2. In addition, 42 LUAD tissues were collected from the Department of Thoracic Surgery, Shandong Provincial Hospital. The prognostic information was also followed up.

Seven immunotherapeutic cohorts were acquired to validate the prediction of immunotherapy efficacy using the MPPS model: advanced urothelial cancer treated with atezolizumab, an anti-PD-L1 antibody (IMvigor210 cohort) [62]; melanoma treated with anti-CTLA4 and anti-PD-1 therapy (GSE91061) [63]; metastatic melanoma treated with anti-CTLA4 therapy [64]; NSCLC treated with nivolumab or pembrolizumab, an anti-PD-1 antibody (GSE126044) [65]; NSCLC treated with anti-PD-1/PD-L1 antibody (GSE135222) [66]; melanoma treated with adoptive T cell therapy (ACT) (GSE100797) [67]; Melanoma treated with anti-PD-1 antibody (GSE78220). The response and benefit of TCGA cohort were calculated based on the Tumor Immune Dysfunction and Exclusion (TIDE) website (http://tide.dfci.harvard.edu/) by integrating TIDE score, interferon gamma (INFG), microsatellite instability (MSI) score, CD274, Merck18, CD8, myeloid-derived suppressor cells (MDSC), cancer associated fibroblast (CAF) and tumor-associated macrophages (TAM) M2.

To generate eQTL instruments for C1QTNF6, genetic variants located within 1000 kb on either side of the coding sequence (in cis) that are robustly associated with gene expression were extracted using eQTLs summary statistics obtained from the eQTLGen Consortium (https://www.eqtlgen.org/cis-eqtls.html). The data were established based on 26,609 blood samples of Europeans [68]. Lung cancer GWAS data (ieu-a-987) were obtained from the Transdisciplinary Research in Cancer of the Lung (TRICL). The GWAS data included 29,863 cases and 55,586 controls from European.

### Estimation of metabolic pathways heterogeneity in LUAD

The levels of 84 metabolic pathways were estimated in each sample by ssGSEA. Then the metabolic differences of LUAD and normal samples were analyzed by “limma” package. An unsupervised consensus clustering according to 84 metabolic pathways scores was performed to identify distinct LUAD metabolic subtypes, which were showed by the principal component analysis (PCA). The metabolic profiles of TME cells including endothelial cells, malignant cells, cancer-associated fibroblasts (CAFs) and pan-immune cells were compared using single-cell RNA sequencing data (GSE111907).

### Development and evaluation of a MPPS system

Pairwise comparisons of the 84 metabolic pathways’ scores in the training cohort were performed. The algorithm presented a scoring system in which the score of the metabolic pathway-pair was recorded as 1 if the expression level of the first metabolic pathway’s score was higher than that of the second; otherwise, it was recorded as 0, resulting in the construction of a 0 or 1 matrix. A metabolic pathway pair was deleted if the proportion of 0 or 1 was more than 80% or less than 20% of the samples in the training cohort. The abbreviations of the qualified metabolic pathway pairs were listed in Supplementary Table 2. Next, the qualified metabolic pathway pairs were enrolled for univariable, LASSO and multivariable Cox regression analysis to construct a MPPS system. The MPPS was calculated as follows:


$$\begin{array}{l} MPPS=\sum_{i=1}^{n}Coef\;(metabolic\;pathway\;pair\;i)\\ \;\;\;\;\;\;\;\;\;\;*\;Value\;(metabolic\;pathway\;pair\;i) \end{array}$$

According to the optimal cut-off point of MPPS determined by the ‘survminer’ package, LUAD patients were stratified into the high- and low-MPPS groups. The optimal cut-off points were determined separately in the training and validation cohorts. The survival rates of the high- and low-MPPS groups were compared by Kaplan-Meier method in the training cohort and validation cohorts. Receiver operating characteristic (ROC) curves, generated by the “timeROC” package and “Aalen” weighting method, and C-index were used to detect the accuracy of MPPS. Univariable and multivariable Cox regression analyses were used to detect the prognostic roles of the clinical characteristics and MPPS. The independent prognostic factors were combined to develop a predicting nomogram by R package “rms”. The calibration curve was used to detect the consistency of the nomogram. The optimal cut-off value was calculated separately for each cancer, when we evaluated the performance of MPPS in pan-cancer.

### Enrichment analysis and functional annotation

GO and KEGG pathway analyses were performed to investigate the variation in biological processes between high- and low-MPPS groups. The results of GO annotation were displayed by an online tool bioinformatics (https://bioinformatics.com.cn/). The Hallmark gene set was used to explore the distinction in various biological signatures between the high- and low-MPPS groups. The CancerSea website (http://biocc.hrbmu.edu.cn/CancerSEA/) was used to investigate 14 biological processes of multiple genes across various cancers.

### Protein-protein interaction (PPI) network

The differentially expressed genes (DEGs) involved in MPPS between high- and low MPPS groups were input in STRING website (https://string-db.org/). PPI network was constructed with a minimum confidence score >0.4 and visualized by the software Cytoscape v3.9.1.

### TME landscape analyses

Immune score, stromal score and ESTIMATE score were calculated using the ESTIMATE algorithm [69]. Immune cells infiltration and functions were evaluated by ssGSEA [70]. Expression of various immune checkpoint genes and HLA-related genes was compared between different MPPS scores groups. The cancer-immunity cycle scores of TCGA-LUAD samples were downloaded from the TIP database (http://biocc.hrbmu.edu.cn/TIP/) [71]. The discrepancy of the cancer-immunity cycle scores between the high- and low-MPPS groups was compared. The differences of MPPS scores among immune-inflamed, excluded and desert phenotypes had been analyzed [62].

### Tumor mutation burden (TMB) and drug sensitivity analyses

The “maftools” R package was employed to explore the mutation frequency in different MPPS subgroups [72]. Then, the correlation between MPSS and TMB was analyzed. Subsequently, we evaluated the synergistic effect of TMB and MPPS score on prognostic stratification. A total of 137 drugs sensitivity in different MPPS groups was analyzed by R package “pRRophetic” and visualized in the form of parliament plot by Hiplot Pro (https://hiplot.com.cn) [73].

### WGCNA

Co-expression gene networks of TCGA-LUAD were constructed using the WGCNA package. The unsigned network was selected. An appropriate soft threshold β was calculated to meet the criteria for the scale-free network. The optimal β was 4. Then, the weighted adjacency matrix was converted into a topological overlap matrix (TOM), and the corresponding dissimilarity was generated (1- TOM). Finally, the dynamic tree cut algorithm was used to identify the modules, and 80 was selected as the minimum number of genes for each module. The modules with the correlation coefficient *R* > 0.2, *P*-value < 0.05 were regarded as the key modules, and the genes with GS > 0.25, MM > 0.2 were regarded as key genes. These genes intersected by TCGA DEGs and GEO genes were used as candidate genes for subsequent analysis.

### Hub genes identified from machine learning-based integrative approaches

Ten machine learning algorithms and 117 algorithm combinations were utilized to identify hub genes related to MPPS with high accuracy and stability performance on prognostic prediction. The integrative algorithms included random survival forest (RSF), stepwise Cox, elastic network (Enet), Lasso, Ridge, CoxBoost, partial least squares regression for Cox (plsRcox), generalised boosted regression modelling (GBM), supervised principal components (SuperPC), and survival support vector machine (survival-SVM). The procedure was as follows: (a) Univariable Cox regression analysis was used to screened out prognostic genes from the candidate genes; (b) Next, 117 algorithm combinations were performed on the prognostic genes to identified hub genes based on the leave-one-out cross-validation (LOOCV) framework in the TCGA-LUAD cohort; (c) All hub genes derived from 117 algorithm combinations were validated in six independent validation cohorts (GSE13213, GSE31210, GSE3141, GSE30219, GSE50081, GSE72094); (d) The hub genes with the highest average C-index across all validation cohorts were considered optimal.

### scRNA-seq analysis

GSE111907 was retrieved to evaluate the metabolic pathway levels and hub genes expression in malignant, pan-immune cells, endothelial and fibroblast cells. The hub genes expression in various cell subtypes of TME was explored by TISCH2 website (http://tisch.comp-genomics.org/). GSE127465 was used to analyze intercellular communication and correlation of hub gene expression and immune cells infiltration using scTIME Portal website (http://sctime.sklehabc.com/unicellular/home).

### RNA extracting and real-time PCR

Total RNA was extracted from LUAD frozen tumor tissues and cells using the AG RNAex Pro Reagent (Accurate Biotechnology (Hunan) Co., Ltd., China). The mRNA (500 ng) was converted into cDNA using Evo MMLVRT Master Mix kit (Accurate Biotechnology (Hunan) Co., Ltd., China). Then, cDNA was amplified with SYBR Premix Ex Tap kit (Accurate Biotechnology (Hunan) Co., Ltd., China). The mRNA levels were assayed by qRT-PCR using the Roche LightCycler® 480 system. 2^-ΔΔCt^ method was used to obtain relative quantitation (RQ) values, with 18S rRNA as endogenous control. The sequences of the primers were listed in Supplementary Table 3.

### Cell culture and transfections

THP-1 cell was purchased from the Procell, Wuhan, China. MRC-5 cell was a gift from Shufang Chen (Shandong Provincial Hospital). MRC-5 cell was cultured in Dulbecco’s Modified Eagle Medium (DMEM) (HyClone, USA), and THP-1 cell was cultured in RPMI H1640 (HyClone, USA), supplemented with 10% fetal bovine serum (FBS) (BI, Israel) in a humidified atmosphere of 5% CO2 and 37° C according to protocol. C1QTNF6 siRNAs (Huzhou Hippo Biotechnology Co., Ltd., Zhejiang Province, China) were transfected into cells using jetPRIME (Polyplus-transfection, Illkirch, France) according to the manual. The sequences of C1QTNF6 siRNAs were as follows.

siC1QTNF6#1:

sense (5’-3’): GGAAUUACAAGGAGACGUA(dT)(dT)

antisense (5’-3’): UACGUCUCCUUGUAAUUCC(dT)(dT)

siC1QTNF6#2:

sense (5’-3’): GGGUCUUUGUGAACCUUGA(dT)(dT)

antisense (5’-3’): UCAAGGUUCACAAAGACCC(dT)(dT)

### Preparation of conditioned medium (CM)

MRC-5 cells were transfected with C1QTNF6 siRNAs for 48h. The medium was replaced using serum-free medium and cells were cultured for additional 24h. Next, the supernatant was centrifuged at 300×g for 5 min and collected to induce TAM polarization and migration.

### Polarization of THP-1 cells

To explore the effects of C1QTNF6 expression of MRC-5 on macrophage polarization, THP-1 cells were induced to M0 macrophage by phorbol 12-myristate 13-acetate (PMA) stimulation for 24h in six-well plates. Then, 2 ml mixture of CM and FBS-containing medium (1:1) was added for 48h. Finally, the total RNA was extracted and the M1- or M2-related markers were detected by qRT-PCR.

### Macrophage migration assay

THP-1 cells were induced to M0 state under PMA stimulation. Then, the M0 macrophages were polarized into M2 macrophages via IL4 and IL13 stimulation. 20,000 M2 macrophages were plated in the upper chamber in the serum-free medium. The lower chambers were filled with 600 μl mixture of CM and FBS-containing medium (1:1). After 48h, the non-migrated cells in the upper chambers were removed and the migrated cells were stained with crystal violet for 30 min. The images were observed by microscope and the numbers of migrated cells were calculated by Image J.

### Instrumental variable selection and Mendelian randomization

Only SNPs of C1QTNF6 cis-eQTL satisfying the following criteria were included as strong instrumental variables: (i) showed genome-wide significant association (*P* < 5 × 10 ^−8^); (ii) showed independent association [linkage disequilibrium (LD) clumping r^2^ < 0.1; kb=500]; (iii) F-statistic > 10; (iv) not a palindromic SNP. Finally, 320 SNPs were identified as strong instrumental variables for C1QTNF6. For the MR analysis, the IVW method is the primary method. In addition, MR Egger, weighted median, simple mode and weighted mode methods were also used to detect the cause effect by R package “TwoSampleMR”. Leave-one-out sensitivity analysis was performed to evaluate the influence of each SNP on the outcome. Heterogeneity and potential horizontal pleiotropy were assessed by the Cochrane’s Q-value and MR-PRESSO global test. Steiger filtering was used to detect the directionality of the association between C1QTNF6 and lung cancer. Bayesian co-localization analyses were used to assess the probability that two traits share the same causal variant using the ‘coloc’ package (https://github.com/chr1swallace/coloc) with default arguments [68]. All SNPs within 1 Mb up and down stream of the leading SNPs were retrieved for colocalization analysis to analyze the posterior probability of H4 (PP.H4) PP.H4 > 80% was defined as having evidence of co-localization.

### Statistical analyses

The statistical analysis of this study was performed using R v4.1.3, GSEA v4.2.3, GraphPad Prism 8 and SPSS v26. For quantitative data, the statistical significance of normally distributed variables was estimated by the Student’s t-test, and non-normally distributed variables were analyzed using the Wilcoxon rank sum test. When comparing between more than two groups, the Kruskal-Wallis test and one-way analysis of variance as non-parametric and parametric methods were made, respectively. Statistical significance was set at *P* < 0.05 unless otherwise stated. False discovery rate (FDR) was used to adjust *P*-value.

### Data availability statement

The datasets presented in this study can be found in online repositories. The names of the repositories and accession numbers have been listed in the article.

## Supplementary Material

Supplementary Figures

Supplementary Table 1

Supplementary Table 2

Supplementary Table 3

## References

[1] Siegel RL, Miller KD, Wagle NS, Jemal A. Cancer statistics, 2023. CA Cancer J Clin. 2023; 73:17–48. 10.3322/caac.21763 36633525

[2] Hutchinson BD, Shroff GS, Truong MT, Ko JP. Spectrum of Lung Adenocarcinoma. Semin Ultrasound CT MR. 2019; 40:255–64. 10.1053/j.sult.2018.11.009 31200873

[3] Hirsch FR, Scagliotti GV, Mulshine JL, Kwon R, Curran WJ Jr, Wu YL, Paz-Ares L. Lung cancer: current therapies and new targeted treatments. Lancet. 2017; 389:299–311. 10.1016/S0140-6736(16)30958-8 27574741

[4] Jiang P, Gu S, Pan D, Fu J, Sahu A, Hu X, Li Z, Traugh N, Bu X, Li B, Liu J, Freeman GJ, Brown MA, et al. Signatures of T cell dysfunction and exclusion predict cancer immunotherapy response. Nat Med. 2018; 24:1550–8. 10.1038/s41591-018-0136-1 30127393 PMC6487502

[5] Vander Heiden MG, DeBerardinis RJ. Understanding the Intersections between Metabolism and Cancer Biology. Cell. 2017; 168:657–69. 10.1016/j.cell.2016.12.039 28187287 PMC5329766

[6] Paul S, Ghosh S, Kumar S. Tumor glycolysis, an essential sweet tooth of tumor cells. Semin Cancer Biol. 2022; 86:1216–30. 10.1016/j.semcancer.2022.09.007 36330953

[7] Bian X, Liu R, Meng Y, Xing D, Xu D, Lu Z. Lipid metabolism and cancer. J Exp Med. 2021; 218:e20201606. 10.1084/jem.20201606 33601415 PMC7754673

[8] Cheng C, Geng F, Cheng X, Guo D. Lipid metabolism reprogramming and its potential targets in cancer. Cancer Commun (Lond). 2018; 38:27. 10.1186/s40880-018-0301-4 29784041 PMC5993136

[9] Yi M, Li J, Chen S, Cai J, Ban Y, Peng Q, Zhou Y, Zeng Z, Peng S, Li X, Xiong W, Li G, Xiang B. Emerging role of lipid metabolism alterations in Cancer stem cells. J Exp Clin Cancer Res. 2018; 37:118. 10.1186/s13046-018-0784-5 29907133 PMC6003041

[10] Sainero-Alcolado L, Liaño-Pons J, Ruiz-Pérez MV, Arsenian-Henriksson M. Targeting mitochondrial metabolism for precision medicine in cancer. Cell Death Differ. 2022; 29:1304–17. 10.1038/s41418-022-01022-y 35831624 PMC9287557

[11] Mullen NJ, Singh PK. Nucleotide metabolism: a pan-cancer metabolic dependency. Nat Rev Cancer. 2023; 23:275–94. 10.1038/s41568-023-00557-7 36973407 PMC10041518

[12] Cristea S, Coles GL, Hornburg D, Gershkovitz M, Arand J, Cao S, Sen T, Williamson SC, Kim JW, Drainas AP, He A, Cam LL, Byers LA, et al. The MEK5-ERK5 Kinase Axis Controls Lipid Metabolism in Small-Cell Lung Cancer. Cancer Res. 2020; 80:1293–303. 10.1158/0008-5472.CAN-19-1027 31969375 PMC7073279

[13] Karasinska JM, Topham JT, Kalloger SE, Jang GH, Denroche RE, Culibrk L, Williamson LM, Wong HL, Lee MKC, O’Kane GM, Moore RA, Mungall AJ, Moore MJ, et al. Altered Gene Expression along the Glycolysis-Cholesterol Synthesis Axis Is Associated with Outcome in Pancreatic Cancer. Clin Cancer Res. 2020; 26:135–46. 10.1158/1078-0432.CCR-19-1543 31481506

[14] Tang Y, Tian W, Xie J, Zou Y, Wang Z, Li N, Zeng Y, Wu L, Zhang Y, Wu S, Xie X, Yang L. Prognosis and Dissection of Immunosuppressive Microenvironment in Breast Cancer Based on Fatty Acid Metabolism-Related Signature. Front Immunol. 2022; 13:843515. 10.3389/fimmu.2022.843515 35432381 PMC9009264

[15] Sukumar M, Roychoudhuri R, Restifo NP. Nutrient Competition: A New Axis of Tumor Immunosuppression. Cell. 2015; 162:1206–8. 10.1016/j.cell.2015.08.064 26359979 PMC6327313

[16] Johnson MO, Wolf MM, Madden MZ, Andrejeva G, Sugiura A, Contreras DC, Maseda D, Liberti MV, Paz K, Kishton RJ, Johnson ME, de Cubas AA, Wu P, et al. Distinct Regulation of Th17 and Th1 Cell Differentiation by Glutaminase-Dependent Metabolism. Cell. 2018; 175:1780–95.e19. 10.1016/j.cell.2018.10.001 30392958 PMC6361668

[17] Shah AM, Wang Z, Ma J. Glutamine Metabolism and Its Role in Immunity, a Comprehensive Review. Animals (Basel). 2020; 10:326. 10.3390/ani10020326 32092847 PMC7070879

[18] Xia L, Oyang L, Lin J, Tan S, Han Y, Wu N, Yi P, Tang L, Pan Q, Rao S, Liang J, Tang Y, Su M, et al. The cancer metabolic reprogramming and immune response. Mol Cancer. 2021; 20:28. 10.1186/s12943-021-01316-8 33546704 PMC7863491

[19] Rostamian H, Khakpoor-Koosheh M, Jafarzadeh L, Masoumi E, Fallah-Mehrjardi K, Tavassolifar MJ, M Pawelek J, Mirzaei HR, Hadjati J. Restricting tumor lactic acid metabolism using dichloroacetate improves T cell functions. BMC Cancer. 2022; 22:39. 10.1186/s12885-021-09151-2 34991504 PMC8734242

[20] Dodard G, Tata A, Erick TK, Jaime D, Miah SMS, Quatrini L, Escalière B, Ugolini S, Vivier E, Brossay L. Inflammation-Induced Lactate Leads to Rapid Loss of Hepatic Tissue-Resident NK Cells. Cell Rep. 2020; 32:107855. 10.1016/j.celrep.2020.107855 32640221 PMC7383148

[21] Lin D, Fan W, Zhang R, Zhao E, Li P, Zhou W, Peng J, Li L. Molecular subtype identification and prognosis stratification by a metabolism-related gene expression signature in colorectal cancer. J Transl Med. 2021; 19:279. 10.1186/s12967-021-02952-w 34193202 PMC8244251

[22] He J, Chen Z, Xue Q, Sun P, Wang Y, Zhu C, Shi W. Identification of molecular subtypes and a novel prognostic model of diffuse large B-cell lymphoma based on a metabolism-associated gene signature. J Transl Med. 2022; 20:186. 10.1186/s12967-022-03393-9 35468826 PMC9036805

[23] Chen DS, Mellman I. Oncology meets immunology: the cancer-immunity cycle. Immunity. 2013; 39:1–10. 10.1016/j.immuni.2013.07.012 23890059

[24] Ferlay J, Colombet M, Soerjomataram I, Mathers C, Parkin DM, Piñeros M, Znaor A, Bray F. Estimating the global cancer incidence and mortality in 2018: GLOBOCAN sources and methods. Int J Cancer. 2019; 144:1941–53. 10.1002/ijc.31937 30350310

[25] Yue T, Chen S, Zhu J, Guo S, Huang Z, Wang P, Zuo S, Liu Y. The aging-related risk signature in colorectal cancer. Aging (Albany NY). 2021; 13:7330–49. 10.18632/aging.202589 33658390 PMC7993742

[26] Jiang C, Liu Y, Wen S, Xu C, Gu L. *In silico* development and clinical validation of novel 8 gene signature based on lipid metabolism related genes in colon adenocarcinoma. Pharmacol Res. 2021; 169:105644. 10.1016/j.phrs.2021.105644 33940186

[27] Wang Z, Jiang Q, Dong C. Metabolic reprogramming in triple-negative breast cancer. Cancer Biol Med. 2020; 17:44–59. 10.20892/j.issn.2095-3941.2019.0210 32296576 PMC7142847

[28] Schiliro C, Firestein BL. Mechanisms of Metabolic Reprogramming in Cancer Cells Supporting Enhanced Growth and Proliferation. Cells. 2021; 10:1056. 10.3390/cells10051056 33946927 PMC8146072

[29] Conte F, van Buuringen N, Voermans NC, Lefeber DJ. Galactose in human metabolism, glycosylation and congenital metabolic diseases: Time for a closer look. Biochim Biophys Acta Gen Subj. 2021; 1865:129898. 10.1016/j.bbagen.2021.129898 33878388

[30] Zheng D, Sussman JH, Jeon MP, Parrish ST, MacMullan MA, Delfarah A, Graham NA. AKT but not MYC promotes reactive oxygen species-mediated cell death in oxidative culture. J Cell Sci. 2020; 133:jcs239277. 10.1242/jcs.239277 32094265

[31] Nitta S, Kandori S, Tanaka K, Sakka S, Siga M, Nagumo Y, Negoro H, Kojima T, Mathis BJ, Shimazui T, Miyamoto T, Matsuzaka T, Shimano H, Nishiyama H. ELOVL5-mediated fatty acid elongation promotes cellular proliferation and invasion in renal cell carcinoma. Cancer Sci. 2022; 113:2738–52. 10.1111/cas.15454 35670054 PMC9357625

[32] Chu Q, Liu P, Song Y, Yang R, An J, Zhai X, Niu J, Yang C, Li B. Stearate-derived very long-chain fatty acids are indispensable to tumor growth. EMBO J. 2023; 42:e111268. 10.15252/embj.2022111268 36408830 PMC9841326

[33] Zhu Z, Cao C, Zhang D, Zhang Z, Liu L, Wu D, Sun J. UBE2T-mediated Akt ubiquitination and Akt/β-catenin activation promotes hepatocellular carcinoma development by increasing pyrimidine metabolism. Cell Death Dis. 2022; 13:154. 10.1038/s41419-022-04596-0 35169125 PMC8847552

[34] Walter M, Herr P. Re-Discovery of Pyrimidine Salvage as Target in Cancer Therapy. Cells. 2022; 11:739. 10.3390/cells11040739 35203388 PMC8870348

[35] Yang C, Zhang J, Liao M, Yang Y, Wang Y, Yuan Y, Ouyang L. Folate-mediated one-carbon metabolism: a targeting strategy in cancer therapy. Drug Discov Today. 2021; 26:817–25. 10.1016/j.drudis.2020.12.006 33316375

[36] Bin P, Huang R, Zhou X. Oxidation Resistance of the Sulfur Amino Acids: Methionine and Cysteine. Biomed Res Int. 2017; 2017:9584932. 10.1155/2017/9584932 29445748 PMC5763110

[37] Upadhyayula PS, Higgins DM, Mela A, Banu M, Dovas A, Zandkarimi F, Patel P, Mahajan A, Humala N, Nguyen TTT, Chaudhary KR, Liao L, Argenziano M, et al. Dietary restriction of cysteine and methionine sensitizes gliomas to ferroptosis and induces alterations in energetic metabolism. Nat Commun. 2023; 14:1187. 10.1038/s41467-023-36630-w 36864031 PMC9981683

[38] Tej GNV, Neogi K, Nayak PK. Caffeine-enhanced anti-tumor activity of anti-PD1 monoclonal antibody. Int Immunopharmacol. 2019; 77:106002. 10.1016/j.intimp.2019.106002 31711939

[39] Lin S, Miao Y, Zheng X, Dong Y, Yang Q, Yang Q, Du S, Xu J, Zhou S, Yuan T. ANGPTL4 negatively regulates the progression of osteosarcoma by remodeling branched-chain amino acid metabolism. Cell Death Discov. 2022; 8:225. 10.1038/s41420-022-01029-x 35461343 PMC9035178

[40] Bartolini D, Sancineto L, Fabro de Bem A, Tew KD, Santi C, Radi R, Toquato P, Galli F. Selenocompounds in Cancer Therapy: An Overview. Adv Cancer Res. 2017; 136:259–302. 10.1016/bs.acr.2017.07.007 29054421

[41] Xu M, Wang X, Li Y, Geng X, Jia X, Zhang L, Yang H. Arachidonic Acid Metabolism Controls Macrophage Alternative Activation Through Regulating Oxidative Phosphorylation in PPARγ Dependent Manner. Front Immunol. 2021; 12:618501. 10.3389/fimmu.2021.618501 34149684 PMC8211451

[42] Lee JY, Nam M, Son HY, Hyun K, Jang SY, Kim JW, Kim MW, Jung Y, Jang E, Yoon SJ, Kim J, Kim J, Seo J, et al. Polyunsaturated fatty acid biosynthesis pathway determines ferroptosis sensitivity in gastric cancer. Proc Natl Acad Sci USA. 2020; 117:32433–42. 10.1073/pnas.2006828117 33288688 PMC7768719

[43] Chen X, Liu Y, Wang Y, Wang C, Chen X, Xiong Y, Liu L, Yuan X, Tang H, Shu C, Zhang J, Guo AM, Chen H, Yang J. CYP4F2-Catalyzed Metabolism of Arachidonic Acid Promotes Stromal Cell-Mediated Immunosuppression in Non-Small Cell Lung Cancer. Cancer Res. 2022; 82:4016–30. 10.1158/0008-5472.CAN-21-4029 36006988

[44] Vardhana SA, Hwee MA, Berisa M, Wells DK, Yost KE, King B, Smith M, Herrera PS, Chang HY, Satpathy AT, van den Brink MRM, Cross JR, Thompson CB. Impaired mitochondrial oxidative phosphorylation limits the self-renewal of T cells exposed to persistent antigen. Nat Immunol. 2020; 21:1022–33. 10.1038/s41590-020-0725-2 32661364 PMC7442749

[45] Scharping NE, Rivadeneira DB, Menk AV, Vignali PDA, Ford BR, Rittenhouse NL, Peralta R, Wang Y, Wang Y, DePeaux K, Poholek AC, Delgoffe GM. Mitochondrial stress induced by continuous stimulation under hypoxia rapidly drives T cell exhaustion. Nat Immunol. 2021; 22:205–15. 10.1038/s41590-020-00834-9 33398183 PMC7971090

[46] Bader JE, Voss K, Rathmell JC. Targeting Metabolism to Improve the Tumor Microenvironment for Cancer Immunotherapy. Mol Cell. 2020; 78:1019–33. 10.1016/j.molcel.2020.05.034 32559423 PMC7339967

[47] Liu X, Hartman CL, Li L, Albert CJ, Si F, Gao A, Huang L, Zhao Y, Lin W, Hsueh EC, Shen L, Shao Q, Hoft DF, et al. Reprogramming lipid metabolism prevents effector T cell senescence and enhances tumor immunotherapy. Sci Transl Med. 2021; 13:eaaz6314. 10.1126/scitranslmed.aaz6314 33790024 PMC12040281

[48] Martínez-Reyes I, Chandel NS. Cancer metabolism: looking forward. Nat Rev Cancer. 2021; 21:669–80. 10.1038/s41568-021-00378-6 34272515

[49] Safrhansova L, Hlozkova K, Starkova J. Targeting amino acid metabolism in cancer. Int Rev Cell Mol Biol. 2022; 373:37–79. 10.1016/bs.ircmb.2022.08.001 36283767

[50] Cumin C, Huang YL, Everest-Dass A, Jacob F. Deciphering the Importance of Glycosphingolipids on Cellular and Molecular Mechanisms Associated with Epithelial-to-Mesenchymal Transition in Cancer. Biomolecules. 2021; 11:62. 10.3390/biom11010062 33418847 PMC7824851

[51] Rao X, Lu Y. C1QTNF6 Targeted by MiR-184 Regulates the Proliferation, Migration, and Invasion of Lung Adenocarcinoma Cells. Mol Biotechnol. 2022; 64:1279–87. 10.1007/s12033-022-00495-z 35578071

[52] Liu W, Zhang J, Xie T, Huang X, Wang B, Tian Y, Yuan Y. C1QTNF6 is a Prognostic Biomarker and Related to Immune Infiltration and Drug Sensitivity: A Pan-Cancer Analysis. Front Pharmacol. 2022; 13:855485. 10.3389/fphar.2022.855485 35401204 PMC8985594

[53] Zhu H, Zheng C, Liu H, Kong F, Kong S, Chen F, Tian Y. Significance of macrophage infiltration in the prognosis of lung adenocarcinoma patients evaluated by scRNA and bulkRNA analysis. Front Immunol. 2022; 13:1028440. 10.3389/fimmu.2022.1028440 36311801 PMC9597471

[54] Chang CP, Su YC, Lee PH, Lei HY. Targeting NFKB by autophagy to polarize hepatoma-associated macrophage differentiation. Autophagy. 2013; 9:619–21. 10.4161/auto.23546 23360732 PMC3627680

[55] Jiang B, Zhu SJ, Xiao SS, Xue M. MiR-217 Inhibits M2-Like Macrophage Polarization by Suppressing Secretion of Interleukin-6 in Ovarian Cancer. Inflammation. 2019; 42:1517–29. 10.1007/s10753-019-01004-2 31049770

[56] Shen M, Xu Z, Xu W, Jiang K, Zhang F, Ding Q, Xu Z, Chen Y. Inhibition of ATM reverses EMT and decreases metastatic potential of cisplatin-resistant lung cancer cells through JAK/STAT3/PD-L1 pathway. J Exp Clin Cancer Res. 2019; 38:149. 10.1186/s13046-019-1161-8 30961670 PMC6454747

[57] Zhang A, Xu Y, Xu H, Ren J, Meng T, Ni Y, Zhu Q, Zhang WB, Pan YB, Jin J, Bi Y, Wu ZB, Lin S, Lou M. Lactate-induced M2 polarization of tumor-associated macrophages promotes the invasion of pituitary adenoma by secreting CCL17. Theranostics. 2021; 11:3839–52. 10.7150/thno.53749 33664865 PMC7914368

[58] Liu F, Qiu H, Xue M, Zhang S, Zhang X, Xu J, Chen J, Yang Y, Xie J. MSC-secreted TGF-β regulates lipopolysaccharide-stimulated macrophage M2-like polarization via the Akt/FoxO1 pathway. Stem Cell Res Ther. 2019; 10:345. 10.1186/s13287-019-1447-y 31771622 PMC6878630

[59] Yang Y, Ye YC, Chen Y, Zhao JL, Gao CC, Han H, Liu WC, Qin HY. Crosstalk between hepatic tumor cells and macrophages via Wnt/β-catenin signaling promotes M2-like macrophage polarization and reinforces tumor malignant behaviors. Cell Death Dis. 2018; 9:793. 10.1038/s41419-018-0818-0 30022048 PMC6052107

[60] Yang Y, Zhang B, Yang Y, Peng B, Ye R. FOXM1 accelerates wound healing in diabetic foot ulcer by inducing M2 macrophage polarization through a mechanism involving SEMA3C/NRP2/Hedgehog signaling. Diabetes Res Clin Pract. 2022; 184:109121. 10.1016/j.diabres.2021.109121 34742786

[61] Lin G, Lin L, Lin H, Xu Y, Chen W, Liu Y, Wu J, Chen S, Lin Q, Zeng Y, Xu Y. C1QTNF6 regulated by miR-29a-3p promotes proliferation and migration in stage I lung adenocarcinoma. BMC Pulm Med. 2022; 22:285. 10.1186/s12890-022-02055-2 35879698 PMC9310408

[62] Mariathasan S, Turley SJ, Nickles D, Castiglioni A, Yuen K, Wang Y, Kadel EE II, Koeppen H, Astarita JL, Cubas R, Jhunjhunwala S, Banchereau R, Yang Y, et al. TGFβ attenuates tumour response to PD-L1 blockade by contributing to exclusion of T cells. Nature. 2018; 554:544–8. 10.1038/nature25501 29443960 PMC6028240

[63] Riaz N, Havel JJ, Makarov V, Desrichard A, Urba WJ, Sims JS, Hodi FS, Martín-Algarra S, Mandal R, Sharfman WH, Bhatia S, Hwu WJ, Gajewski TF, et al. Tumor and Microenvironment Evolution during Immunotherapy with Nivolumab. Cell. 2017; 171:934–49.e16. 10.1016/j.cell.2017.09.028 29033130 PMC5685550

[64] Van Allen EM, Miao D, Schilling B, Shukla SA, Blank C, Zimmer L, Sucker A, Hillen U, Foppen MHG, Goldinger SM, Utikal J, Hassel JC, Weide B, et al. Genomic correlates of response to CTLA-4 blockade in metastatic melanoma. Science. 2015; 350:207–11. 10.1126/science.aad0095 26359337 PMC5054517

[65] Cho JW, Hong MH, Ha SJ, Kim YJ, Cho BC, Lee I, Kim HR. Genome-wide identification of differentially methylated promoters and enhancers associated with response to anti-PD-1 therapy in non-small cell lung cancer. Exp Mol Med. 2020; 52:1550–63. 10.1038/s12276-020-00493-8 32879421 PMC8080767

[66] Jung H, Kim HS, Kim JY, Sun JM, Ahn JS, Ahn MJ, Park K, Esteller M, Lee SH, Choi JK. DNA methylation loss promotes immune evasion of tumours with high mutation and copy number load. Nat Commun. 2019; 10:4278. 10.1038/s41467-019-12159-9 31537801 PMC6753140

[67] Lauss M, Donia M, Harbst K, Andersen R, Mitra S, Rosengren F, Salim M, Vallon-Christersson J, Törngren T, Kvist A, Ringnér M, Svane IM, Jönsson G. Mutational and putative neoantigen load predict clinical benefit of adoptive T cell therapy in melanoma. Nat Commun. 2017; 8:1738. 10.1038/s41467-017-01460-0 29170503 PMC5701046

[68] Võsa U, Claringbould A, Westra HJ, Bonder MJ, Deelen P, Zeng B, Kirsten H, Saha A, Kreuzhuber R, Yazar S, Brugge H, Oelen R, de Vries DH, et al, and BIOS Consortium, and i2QTL Consortium. Large-scale cis- and trans-eQTL analyses identify thousands of genetic loci and polygenic scores that regulate blood gene expression. Nat Genet. 2021; 53:1300–10. 10.1038/s41588-021-00913-z 34475573 PMC8432599

[69] Yoshihara K, Shahmoradgoli M, Martínez E, Vegesna R, Kim H, Torres-Garcia W, Treviño V, Shen H, Laird PW, Levine DA, Carter SL, Getz G, Stemke-Hale K, et al. Inferring tumour purity and stromal and immune cell admixture from expression data. Nat Commun. 2013; 4:2612. 10.1038/ncomms3612 24113773 PMC3826632

[70] Hänzelmann S, Castelo R, Guinney J. GSVA: gene set variation analysis for microarray and RNA-seq data. BMC Bioinformatics. 2013; 14:7. 10.1186/1471-2105-14-7 23323831 PMC3618321

[71] Xu L, Deng C, Pang B, Zhang X, Liu W, Liao G, Yuan H, Cheng P, Li F, Long Z, Yan M, Zhao T, Xiao Y, Li X. TIP: A Web Server for Resolving Tumor Immunophenotype Profiling. Cancer Res. 2018; 78:6575–80. 10.1158/0008-5472.CAN-18-0689 30154154

[72] Mayakonda A, Lin DC, Assenov Y, Plass C, Koeffler HP. Maftools: efficient and comprehensive analysis of somatic variants in cancer. Genome Res. 2018; 28:1747–56. 10.1101/gr.239244.118 30341162 PMC6211645

[73] Geeleher P, Cox N, Huang RS. pRRophetic: an R package for prediction of clinical chemotherapeutic response from tumor gene expression levels. PLoS One. 2014; 9:e107468. 10.1371/journal.pone.0107468 25229481 PMC4167990

