# Supplementary material for: Prediction of prognosis and immunotherapy efficacy based on metabolic landscape in lung adenocarcinoma by bulk, single-cell RNA sequencing and Mendelian randomization analyses
Source: Aging (Albany NY). 2024 May 20;16(10):8772–809. doi: 10.18632/aging.205838 (PMC11164486; doi:10.18632/aging.205838)
Supplement: Supplementary Figures [file aging-16-205838-s001.pdf]

SUPPLEMENTARY FIGURES

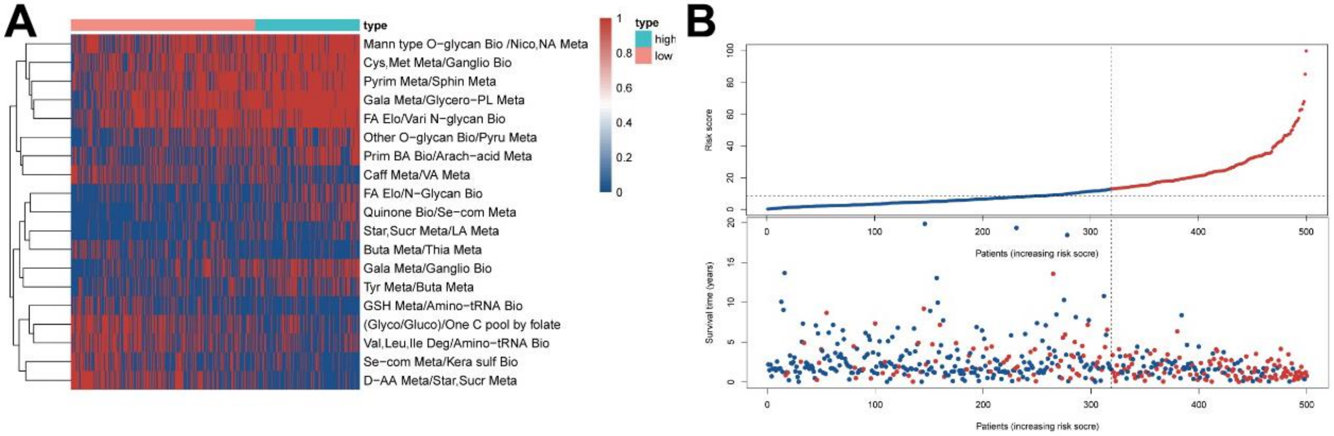

Supplementary Figure 1. Differences of metabolic pathway pairs, survival time and status between the high- and low-MPPS groups. (**A**) Differences of 19 metabolic pathway pairs between the high- and low-MPPS groups. (**B**) The risk plot of MMPS indicated that as MMPS increased, OS time decreased while mortality rose.

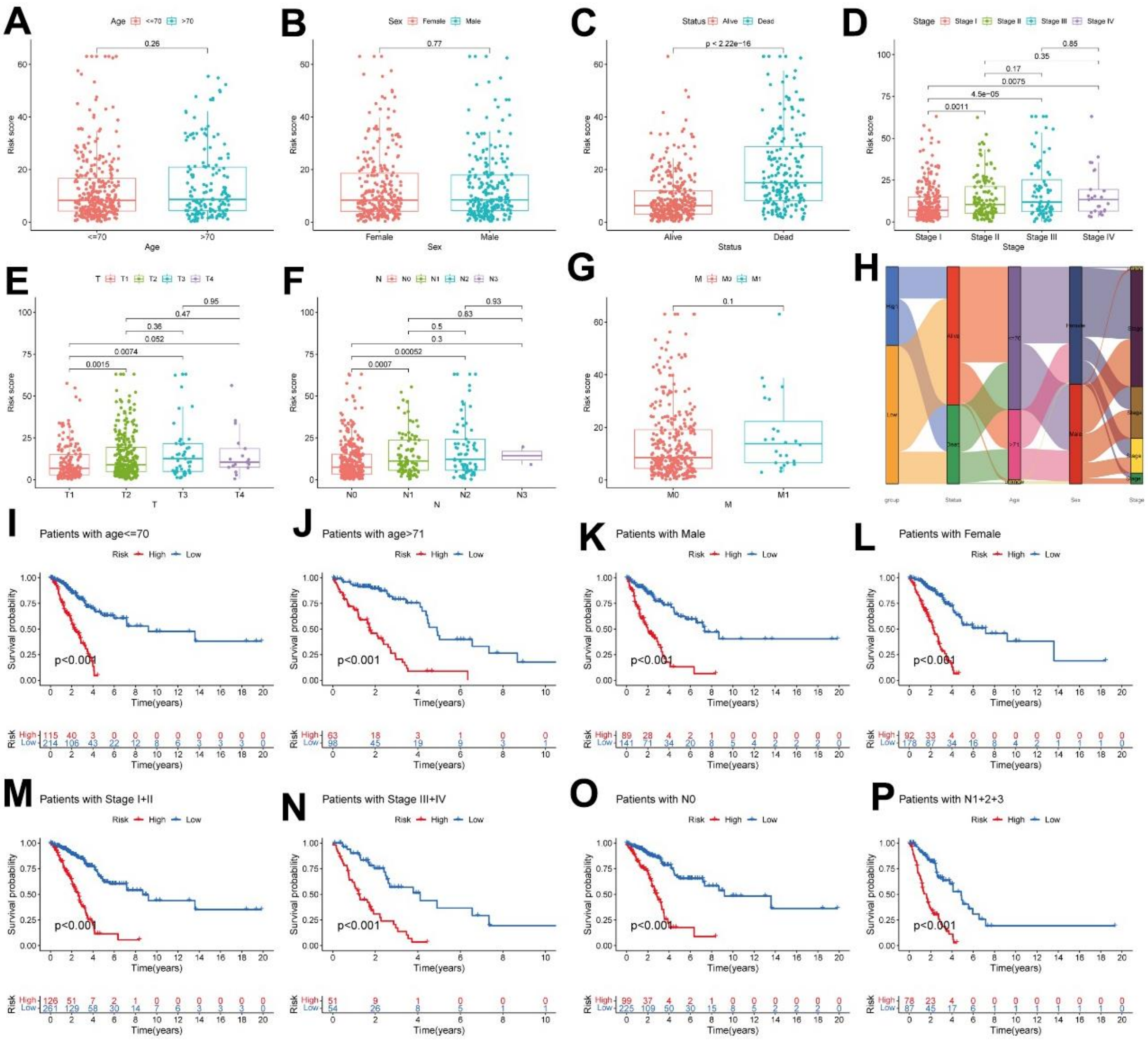

Supplementary Figure 2. The correlation of MPPS and clinical traits, and its performance in clinical subgroups. Differences of MPPS between different ages (**A**), sexes (**B**), survival status (**C**), stages (**D**), T stages (**E**), N stages (**F**), and M stages (**G**). (**H**) Sankey diagram showed the relationship of MPPS groups, survival status, age, sex, and stage. The Kaplan-Meier survival curves of the high- and low-MPPS groups in different clinical subgroups. Age <= 70 (**I**), Age > 70 (**J**), Male (**K**), Female (**L**), Stage I+II (**M**), Stage III+IV (**N**), N0 stage (**O**), N1+N2+N3 stage (**P**).

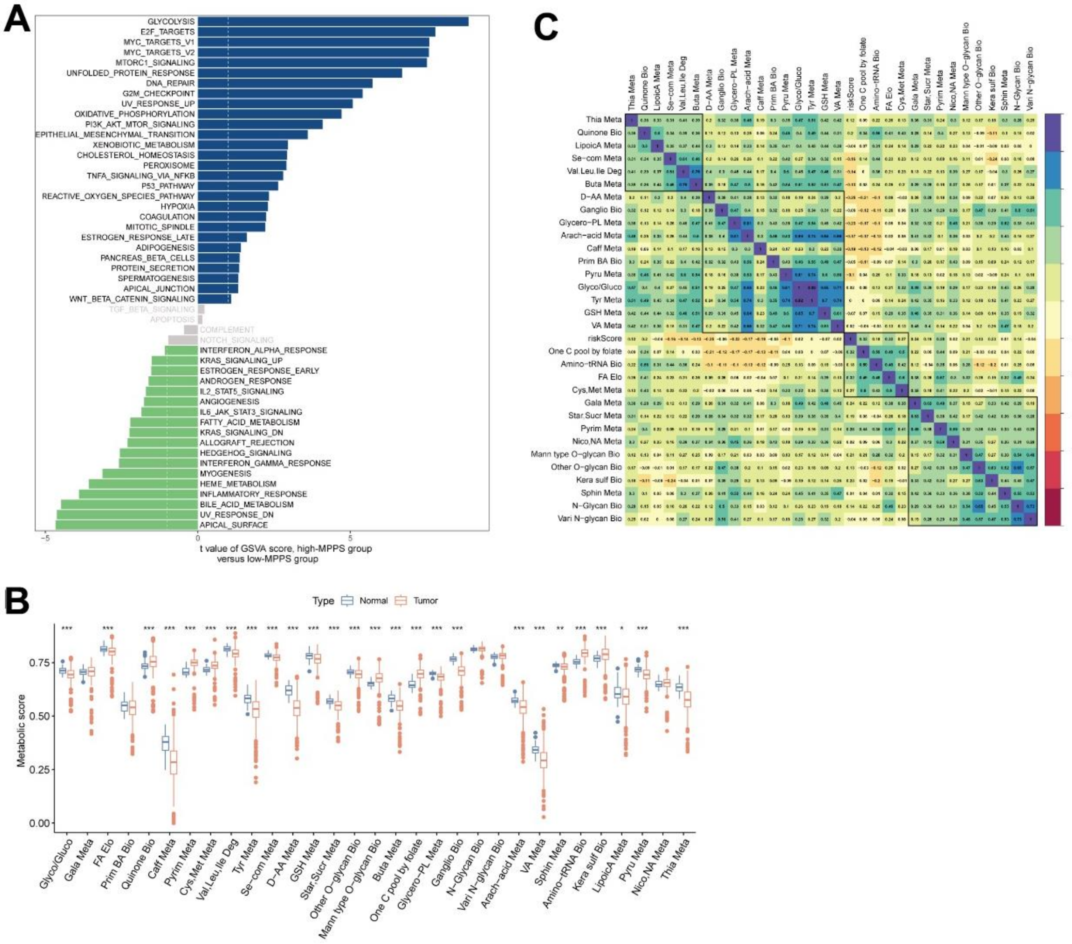

Supplementary Figure 3. The hallmarks enrichment and metabolic pathways variation between the high- and low-MPPS groups. (**A**) The hallmarks enrichment between the high- and low-MPPS groups. (**B**) The differences of metabolic pathways between TCGA-LUAD and normal tissues. (**C**) The correlation heatmap of MPPS and metabolic pathways. $*P < 0.05$, $**P < 0.01$, $***P < 0.001$.

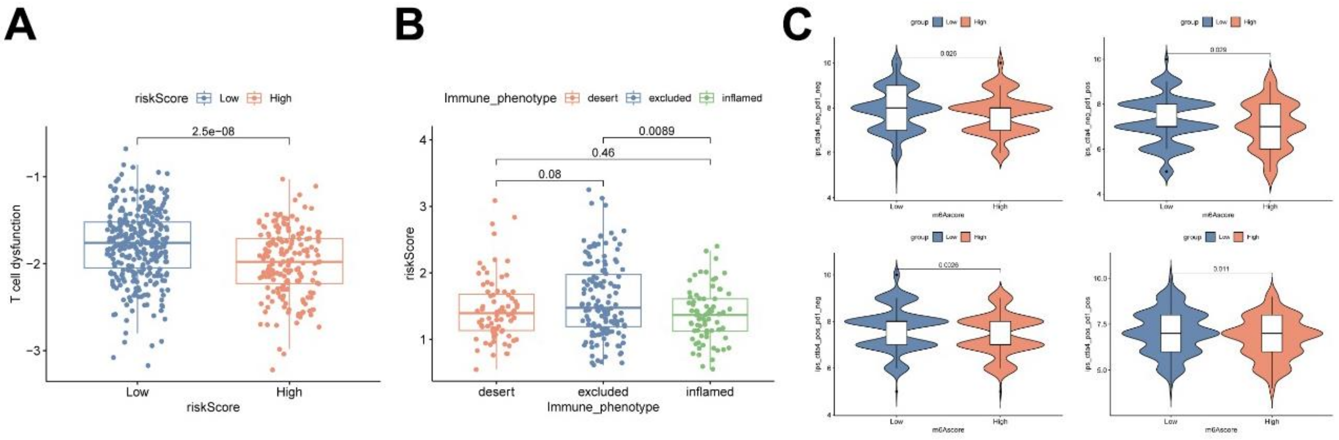

Supplementary Figure 4. TIDE scores and IPS of the high- and low-MPPS groups. (**A**) T cell dysfunction scores of the high- and low-MPPS groups. (**B**) MPPS of desert, excluded, inflamed immune phenotypes. (**C**) IPS of the high- and low-MPPS groups.

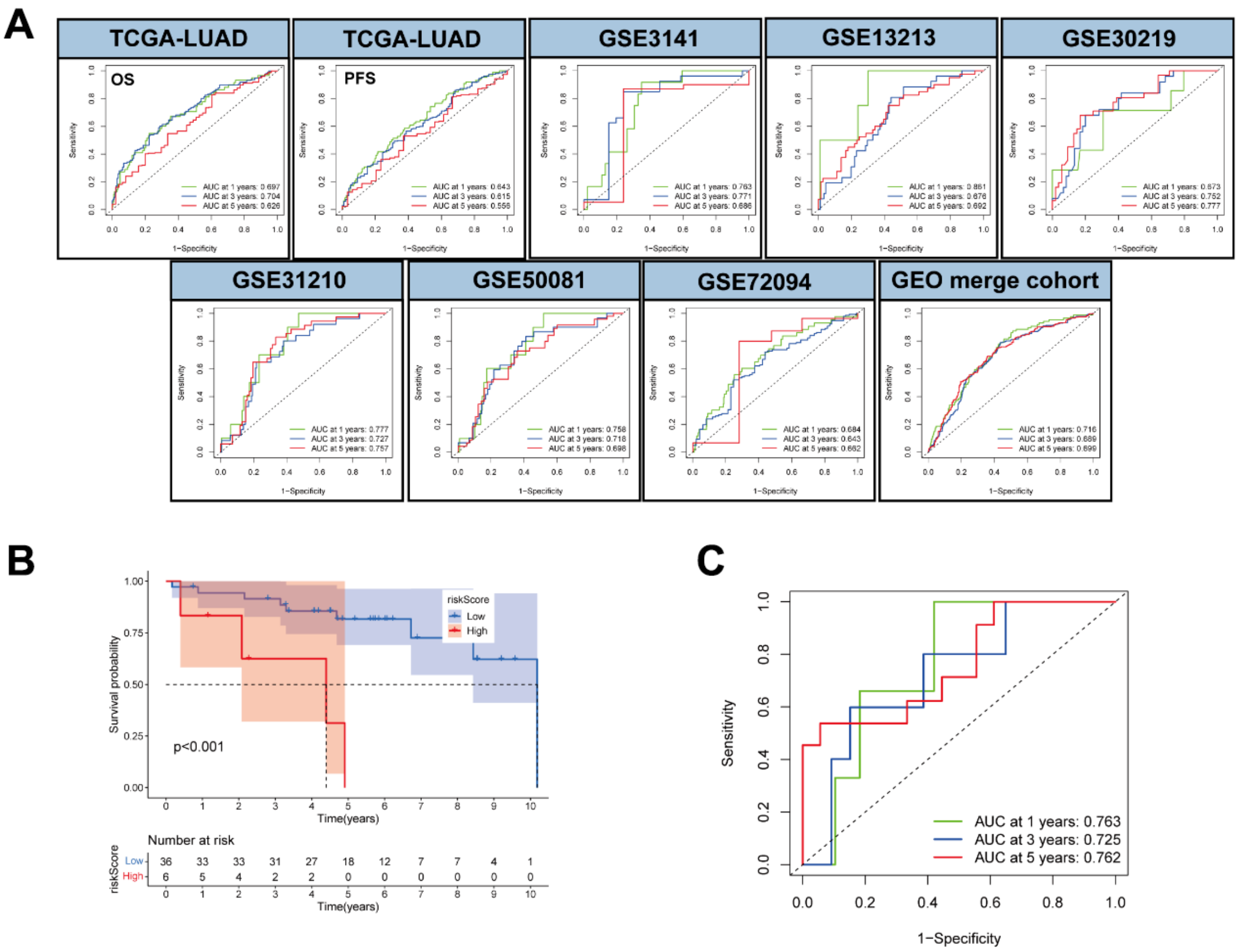

Supplementary Figure 5. Evaluation of MPPS-related gene signature. (**A**) The 1-, 3-, 5-year ROC curves of MPPS-related gene signature in the training and validation cohorts. (**B**) The Kaplan-Meier survival curves of the high- and low-gene risk score groups in 42 LUAD patients by qRT-PCR. (**C**) The 1-, 3-, 5-year ROC curves of OS of 42 LUAD patients predicted by MPPS-related gene signature.

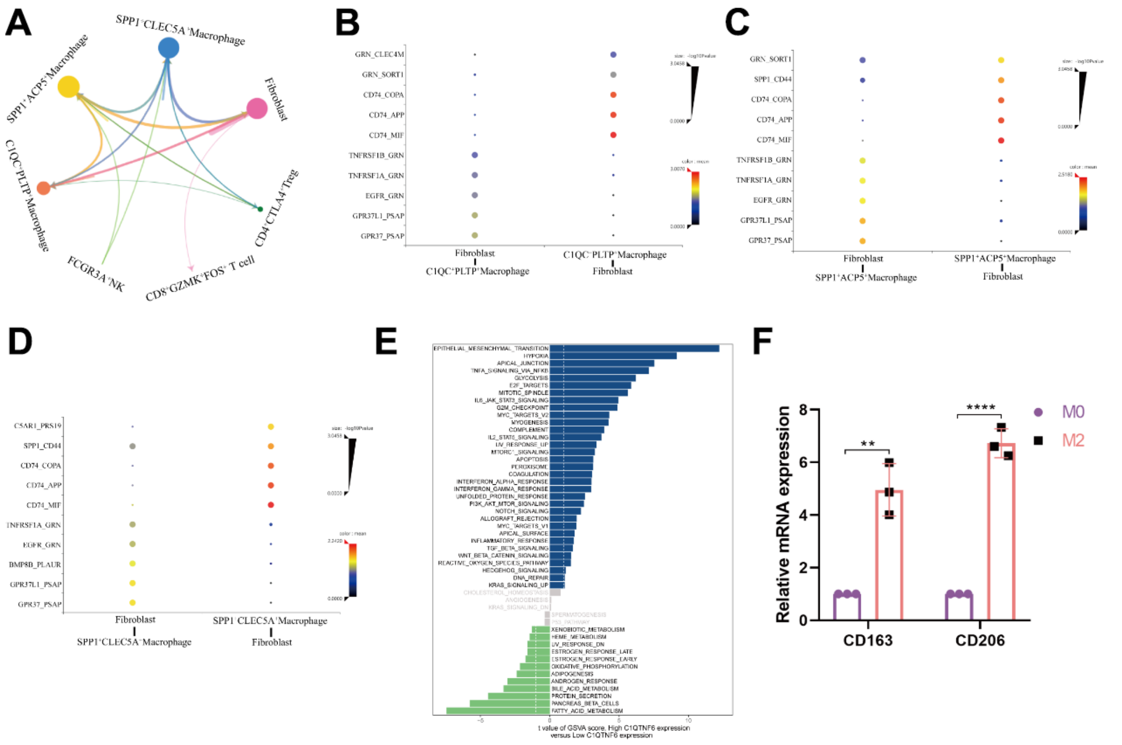

Supplementary Figure 6. Intercellular communications between fibroblasts and immune cells in LUAD TME. (**A**) Intercellular communications network of fibroblasts and immune cells in LUAD TME. (**B**–**D**) The ligand-receptor interaction between fibroblasts and M2 macrophages. (**E**) The hallmarks enrichment between the high- and low-C1QTNF6 expression groups in TCGA-LUAD. (**F**) M2 macrophage markers expression of induced M0 and M2 macrophages by qRT-PCR.

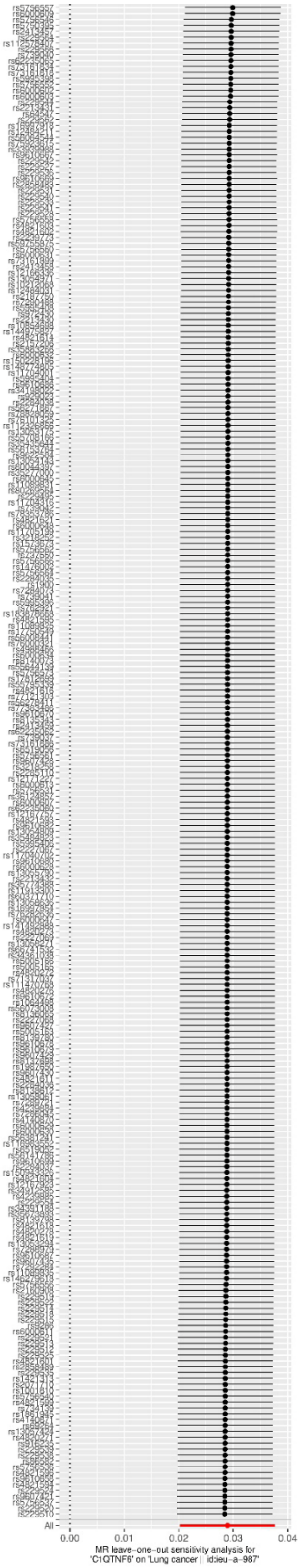

Supplementary Figure 7. Leave-one-out sensitivity analysis of instrumental variables for C1QTNF6.
