# Supplementary material for: Prediction of prognosis and immunotherapy efficacy based on metabolic landscape in lung adenocarcinoma by bulk, single-cell RNA sequencing and Mendelian randomization analyses
Source: Aging (Albany NY). 2024 May 20;16(10):8772–809. doi: 10.18632/aging.205838 (PMC11164486; doi:10.18632/aging.205838)
Supplement: Supplementary Table 1 [file aging-16-205838-s002.pdf]

SUPPLEMENTARY TABLES

Supplementary Table 1. The clinical information data of LAUD patients from TCGA and GEO databases.

Variables	Discovery cohort TCGA (500)	Validation cohort GEO (1009)
**Gender**		
Male	230 (54.00%)	471 (46.70%)
Female	270 (46.00%)	480 (47.60%)
NA	-	58 (5.70%)
**Age at Diagnosis**		
Mean (SD)	65.26 (10.05)	64.62 (10.11)
**Median Survival Time**		
OS, Days (IQR)	654.50 (707)	1114 (1172)
**Survival Event**		
Alive	318 (63.60%)	686 (68.00%)
Dead	182 (36.40%)	323 (32.00%)
**Stage**		
I	266 (53.20%)	593 (58.80%)
II	119 (23.80%)	173 (17.10%)
III	81 (16.20%)	82 (8.10%)
IV	26 (5.20%)	15 (1.50%)
NA	8 (1.60%)	146 (14.50%)

SD, standard deviation; IQR, inter-quartile range.
