# Supplementary material for: Prediction of prognosis and immunotherapy efficacy based on metabolic landscape in lung adenocarcinoma by bulk, single-cell RNA sequencing and Mendelian randomization analyses
Source: Aging (Albany NY). 2024 May 20;16(10):8772–809. doi: 10.18632/aging.205838 (PMC11164486; doi:10.18632/aging.205838)
Supplement: Supplementary Table 2 [file aging-16-205838-s003.docx]

**Supplementary table 2. The abbreviations and matched full names of metabolic pathways and metabolic pathway pairs**

| **Metabolic pathways (pairs)** | **Abbreviations** |
| --- | --- |
| Glycolysis / Gluconeogenesis | Glyco/Gluco |
| Citrate cycle (TCA cycle) | TCA cycle |
| Pentose phosphate pathway | PPP |
| Pentose and glucuronate interconversions | Pent,Gluc conv |
| Fructose and mannose metabolism | Fruc,Mann Meta |
| Galactose metabolism | Gala Meta |
| Ascorbate and aldarate metabolism | Asc, Ald Meta |
| Fatty acid biosynthesis | FA Bio |
| Fatty acid elongation | FA Elo |
| Fatty acid degradation | FA Deg |
| Steroid biosynthesis | Steroid Bio |
| Primary bile acid biosynthesis | Prim BA Bio |
| Ubiquinone and other terpenoid-quinone biosynthesis | Quinone Bio |
| Steroid hormone biosynthesis | Steroid horm Bio |
| Oxidative phosphorylation | OXPHOS |
| Arginine biosynthesis | Arg Bio |
| Purine metabolism | Purine Meta |
| Caffeine metabolism | Caff Meta |
| Pyrimidine metabolism | Pyrim Meta |
| Alanine, aspartate and glutamate metabolism | Ala, Asp,Glu Meta |
| Glycine, serine and threonine metabolism | Gly,Ser,Thr Meta |
| Cysteine and methionine metabolism | Cys,Met Meta |
| Valine, leucine and isoleucine degradation | Val,Leu,Ile Deg |
| Valine, leucine and isoleucine biosynthesis | Val,Leu,Ile Bio |
| Lysine degradation | Lys Deg |
| Arginine and proline metabolism | Arg,Pro Meta |
| Histidine metabolism | His Meta |
| Tyrosine metabolism | Tyr Meta |
| Phenylalanine metabolism | Phe Meta |
| Tryptophan metabolism | Trp Meta |
| Phenylalanine, tyrosine and tryptophan biosynthesis | Phe,Tyr,Trp Bio |
| beta-Alanine metabolism | β-Ala Meta |
| Taurine and hypotaurine metabolism | Taur,hypo Meta |
| Phosphonate and phosphinate metabolism | Phos, Phos Meta |
| Selenocompound metabolism | Se-com Meta |
| D-Amino acid metabolism | D-AA Meta |
| Glutathione metabolism | GSH Meta |
| Starch and sucrose metabolism | Star,Sucr Meta |
| N-Glycan biosynthesis | N-Glycan Bio |
| Other glycan degradation | Other glycan Deg |
| Mucin type O-glycan biosynthesis | Muc type O-glycan Bio |
| Various types of N-glycan biosynthesis | Vari N-glycan Bio |
| Other types of O-glycan biosynthesis | Other O-glycan Bio |
| Mannose type O-glycan biosynthesis | Mann type O-glycan Bio |
| Amino sugar and nucleotide sugar metabolism | AS,NS Meta |
| Neomycin, kanamycin and gentamicin biosynthesis | Neo,Kana,gentamicin Bio |
| Glycosaminoglycan degradation | GAG Deg |
| Glycosaminoglycan biosynthesis - chondroitin sulfate / dermatan sulfate | Chon sulf/Derm sulf Bio |
| Glycosaminoglycan biosynthesis - keratan sulfate | Kera sulf Bio |
| Glycosaminoglycan biosynthesis - heparan sulfate / heparin | hepa sulf/heparin Bio |
| Glycerolipid metabolism | GL Meta |
| Inositol phosphate metabolism | IP Meta |
| Glycosylphosphatidylinositol (GPI)-anchor biosynthesis | Anchor Bio |
| Glycerophospholipid metabolism | Glycero-PL Meta |
| Ether lipid metabolism | EL Meta |
| Arachidonic acid metabolism | Arach-acid Meta |
| Linoleic acid metabolism | LA Meta |
| alpha-Linolenic acid metabolism | α-LA Meta |
| Sphingolipid metabolism | Sphin Meta |
| Glycosphingolipid biosynthesis - lacto and neolacto series | lacto,neol Bio |
| Glycosphingolipid biosynthesis - globo and isoglobo series | Globo,Isog Bio |
| Glycosphingolipid biosynthesis - ganglio series | Ganglio Bio |
| Pyruvate metabolism | Pyru Meta |
| Glyoxylate and dicarboxylate metabolism | Glyo,Dicar Meta |
| Propanoate metabolism | Prop Meta |
| Butanoate metabolism | Buta Meta |
| One carbon pool by folate | One C pool by folate |
| Thiamine metabolism | Thia Meta |
| Riboflavin metabolism | VB2 Meta |
| Vitamin B6 metabolism | VB6 Meta |
| Nicotinate and nicotinamide metabolism | Nico,NA Meta |
| Pantothenate and CoA biosynthesis | Pant and CoA Bio |
| Biotin metabolism | Biotin Meta |
| Lipoic acid metabolism | LipoicA Meta |
| Folate biosynthesis | Folate Bio |
| Retinol metabolism | VA Meta |
| Porphyrin metabolism | Porp Meta |
| Terpenoid backbone biosynthesis | TB Bio |
| Nitrogen metabolism | Nitr Meta |
| Sulfur metabolism | Sulfur Meta |
| Aminoacyl-tRNA biosynthesis | Amino-tRNA Bio |
| Metabolism of xenobiotics by cytochrome P450 | Xeno Meta by CYP450 |
| Drug metabolism - cytochrome P450 | Drug-CYP450 |
| Drug metabolism - other enzymes | Drug-other enzy |
| (Glycolysis/Gluconeogenesis)/Galactose metabolism | (Glyco/Gluco)/Gala Meta |
| (Glycolysis/Gluconeogenesis)/Arginine and proline metabolism | (Glyco/Gluco)/Arg,Pro Meta |
| (Glycolysis/Gluconeogenesis)/Phosphonate and phosphinate metabolism | (Glyco/Gluco)/Phos, Phos Meta |
| (Glycolysis/Gluconeogenesis)/Other types of O-glycan biosynthesis | (Glyco/Gluco)/Other O-glycan Bio |
| (Glycolysis/Gluconeogenesis)/Mannose type O-glycan biosynthesis | (Glyco/Gluco)/Mann type O-glycan Bio |
| (Glycolysis/Gluconeogenesis)/Neomycin, kanamycin and gentamicin biosynthesis | (Glyco/Gluco)/Neo,Kana,gentamicin Bio |
| (Glycolysis/Gluconeogenesis)/Glycerophospholipid metabolism | (Glyco/Gluco)/Glycero-PL Meta |
| (Glycolysis/Gluconeogenesis)/Glycosphingolipid biosynthesis - ganglio series | (Glyco/Gluco)/Ganglio Bio |
| (Glycolysis/Gluconeogenesis)/Pyruvate metabolism | (Glyco/Gluco)/Pyru Meta |
| (Glycolysis/Gluconeogenesis)/One carbon pool by folate | (Glyco/Gluco)/One C pool by folate |
| (Glycolysis/Gluconeogenesis)/Vitamin B6 metabolism | (Glyco/Gluco)/VB6 Meta |
| (Glycolysis/Gluconeogenesis)/Pantothenate and CoA biosynthesis | (Glyco/Gluco)/Pant and CoA Bio |
| Citrate cycle (TCA cycle)/Riboflavin metabolism | TCA cycle/VB2 Meta |
| Pentose phosphate pathway/Fructose and mannose metabolism | PPP/Fruc,Mann Meta |
| Pentose phosphate pathway/Fatty acid degradation | PPP/FA Deg |
| Pentose phosphate pathway/Ubiquinone and other terpenoid-quinone biosynthesis | PPP/Quinone Bio |
| Pentose phosphate pathway/Valine, leucine and isoleucine biosynthesis | PPP/Val,Leu,Ile Bio |
| Pentose phosphate pathway/Selenocompound metabolism | PPP/Se-com Meta |
| Pentose phosphate pathway/Glutathione metabolism | PPP/GSH Meta |
| Pentose phosphate pathway/Various types of N-glycan biosynthesis | PPP/Vari N-glycan Bio |
| Pentose phosphate pathway/Glycosaminoglycan biosynthesis - chondroitin sulfate/dermatan sulfate | PPP/Chon sulf/Derm sulf Bio |
| Pentose phosphate pathway/Glycosaminoglycan biosynthesis - keratan sulfate | PPP/Kera sulf Bio |
| Pentose phosphate pathway/Inositol phosphate metabolism | PPP/IP Meta |
| Pentose phosphate pathway/Glycosylphosphatidylinositol (GPI)-anchor biosynthesis | PPP/Anchor Bio |
| Pentose phosphate pathway/Propanoate metabolism | PPP/Prop Meta |
| Pentose phosphate pathway/Biotin metabolism | PPP/Biotin Meta |
| Pentose phosphate pathway/Aminoacyl-tRNA biosynthesis | PPP/Amino-tRNA Bio |
| Pentose and glucuronate interconversions/Caffeine metabolism | Pent,Gluc conv/Caff Meta |
| Pentose and glucuronate interconversions/Linoleic acid metabolism | Pent,Gluc conv/LA Meta |
| Pentose and glucuronate interconversions/Retinol metabolism | Pent,Gluc conv/VA Meta |
| Fructose and mannose metabolism/Fatty acid elongation | Fruc,Mann Meta/FA Elo |
| Fructose and mannose metabolism/Fatty acid degradation | Fruc,Mann Meta/FA Deg |
| Fructose and mannose metabolism/Ubiquinone and other terpenoid-quinone biosynthesis | Fruc,Mann Meta/Quinone Bio |
| Fructose and mannose metabolism/Valine, leucine and isoleucine degradation | Fruc,Mann Meta/Val,Leu,Ile Deg |
| Fructose and mannose metabolism/Valine, leucine and isoleucine biosynthesis | Fruc,Mann Meta/Val,Leu,Ile Bio |
| Fructose and mannose metabolism/Selenocompound metabolism | Fruc,Mann Meta/Se-com Meta |
| Fructose and mannose metabolism/Glutathione metabolism | Fruc,Mann Meta/GSH Meta |
| Fructose and mannose metabolism/Various types of N-glycan biosynthesis | Fruc,Mann Meta/Vari N-glycan Bio |
| Fructose and mannose metabolism/Glycosaminoglycan biosynthesis - chondroitin sulfate/dermatan sulfate | Fruc,Mann Meta/Chon sulf/Derm sulf Bio |
| Fructose and mannose metabolism/Glycosaminoglycan biosynthesis - keratan sulfate | Fruc,Mann Meta/Kera sulf Bio |
| Fructose and mannose metabolism/Glycosylphosphatidylinositol (GPI)-anchor biosynthesis | Fruc,Mann Meta/Anchor Bio |
| Fructose and mannose metabolism/Propanoate metabolism | Fruc,Mann Meta/Prop Meta |
| Fructose and mannose metabolism/Biotin metabolism | Fruc,Mann Meta/Biotin Meta |
| Fructose and mannose metabolism/Aminoacyl-tRNA biosynthesis | Fruc,Mann Meta/Amino-tRNA Bio |
| Galactose metabolism/Arginine and proline metabolism | Gala Meta/Arg,Pro Meta |
| Galactose metabolism/Phosphonate and phosphinate metabolism | Gala Meta/Phos, Phos Meta |
| Galactose metabolism/Other types of O-glycan biosynthesis | Gala Meta/Other O-glycan Bio |
| Galactose metabolism/Mannose type O-glycan biosynthesis | Gala Meta/Mann type O-glycan Bio |
| Galactose metabolism/Neomycin, kanamycin and gentamicin biosynthesis | Gala Meta/Neo,Kana,gentamicin Bio |
| Galactose metabolism/Glycosaminoglycan degradation | Gala Meta/GAG Deg |
| Galactose metabolism/Glycerophospholipid metabolism | Gala Meta/Glycero-PL Meta |
| Galactose metabolism/Glycosphingolipid biosynthesis - ganglio series | Gala Meta/Ganglio Bio |
| Galactose metabolism/Pyruvate metabolism | Gala Meta/Pyru Meta |
| Galactose metabolism/One carbon pool by folate | Gala Meta/One C pool by folate |
| Galactose metabolism/Vitamin B6 metabolism | Gala Meta/VB6 Meta |
| Galactose metabolism/Pantothenate and CoA biosynthesis | Gala Meta/Pant and CoA Bio |
| Ascorbate and aldarate metabolism/Steroid hormone biosynthesis | Asc, Ald Meta/Steroid horm Bio |
| Ascorbate and aldarate metabolism/Caffeine metabolism | Asc, Ald Meta/Caff Meta |
| Ascorbate and aldarate metabolism/Retinol metabolism | Asc, Ald Meta/VA Meta |
| Fatty acid biosynthesis/Purine metabolism | FA Bio/Purine Meta |
| Fatty acid biosynthesis/Alanine, aspartate and glutamate metabolism | FA Bio/Ala, Asp,Glu Meta |
| Fatty acid biosynthesis/Histidine metabolism | FA Bio/His Meta |
| Fatty acid biosynthesis/Phenylalanine metabolism | FA Bio/Phe Meta |
| Fatty acid biosynthesis/Tryptophan metabolism | FA Bio/Trp Meta |
| Fatty acid biosynthesis/beta-Alanine metabolism | FA Bio/β-Ala Meta |
| Fatty acid biosynthesis/Mucin type O-glycan biosynthesis | FA Bio/Muc type O-glycan Bio |
| Fatty acid biosynthesis/Mannose type O-glycan biosynthesis | FA Bio/Mann type O-glycan Bio |
| Fatty acid biosynthesis/Ether lipid metabolism | FA Bio/EL Meta |
| Fatty acid biosynthesis/Glycosphingolipid biosynthesis - globo and isoglobo series | FA Bio/Globo,Isog Bio |
| Fatty acid biosynthesis/Nicotinate and nicotinamide metabolism | FA Bio/Nico,NA Meta |
| Fatty acid biosynthesis/Folate biosynthesis | FA Bio/Folate Bio |
| Fatty acid elongation/Steroid biosynthesis | FA Elo/Steroid Bio |
| Fatty acid elongation/Valine, leucine and isoleucine degradation | FA Elo/Val,Leu,Ile Deg |
| Fatty acid elongation/Valine, leucine and isoleucine biosynthesis | FA Elo/Val,Leu,Ile Bio |
| Fatty acid elongation/N-Glycan biosynthesis | FA Elo/N-Glycan Bio |
| Fatty acid elongation/Other glycan degradation | FA Elo/Other glycan Deg |
| Fatty acid elongation/Various types of N-glycan biosynthesis | FA Elo/Vari N-glycan Bio |
| Fatty acid elongation/Amino sugar and nucleotide sugar metabolism | FA Elo/AS,NS Meta |
| Fatty acid elongation/Glycosaminoglycan biosynthesis - chondroitin sulfate/dermatan sulfate | FA Elo/Chon sulf/Derm sulf Bio |
| Fatty acid elongation/Glycosaminoglycan biosynthesis - keratan sulfate | FA Elo/Kera sulf Bio |
| Fatty acid elongation/Glycosylphosphatidylinositol (GPI)-anchor biosynthesis | FA Elo/Anchor Bio |
| Fatty acid elongation/Terpenoid backbone biosynthesis | FA Elo/TB Bio |
| Fatty acid elongation/Aminoacyl-tRNA biosynthesis | FA Elo/Amino-tRNA Bio |
| Fatty acid degradation/Ubiquinone and other terpenoid-quinone biosynthesis | FA Deg/Quinone Bio |
| Fatty acid degradation/Pyrimidine metabolism | FA Deg/Pyrim Meta |
| Fatty acid degradation/Cysteine and methionine metabolism | FA Deg/Cys,Met Meta |
| Fatty acid degradation/Valine, leucine and isoleucine biosynthesis | FA Deg/Val,Leu,Ile Bio |
| Fatty acid degradation/Lysine degradation | FA Deg/Lys Deg |
| Fatty acid degradation/Selenocompound metabolism | FA Deg/Se-com Meta |
| Fatty acid degradation/Glutathione metabolism | FA Deg/GSH Meta |
| Fatty acid degradation/Various types of N-glycan biosynthesis | FA Deg/Vari N-glycan Bio |
| Fatty acid degradation/Neomycin, kanamycin and gentamicin biosynthesis | FA Deg/Neo,Kana,gentamicin Bio |
| Fatty acid degradation/Glycosaminoglycan degradation | FA Deg/GAG Deg |
| Fatty acid degradation/Glycosaminoglycan biosynthesis - chondroitin sulfate/dermatan sulfate | FA Deg/Chon sulf/Derm sulf Bio |
| Fatty acid degradation/Glycosaminoglycan biosynthesis - keratan sulfate | FA Deg/Kera sulf Bio |
| Fatty acid degradation/Inositol phosphate metabolism | FA Deg/IP Meta |
| Fatty acid degradation/Glycosylphosphatidylinositol (GPI)-anchor biosynthesis | FA Deg/Anchor Bio |
| Fatty acid degradation/Sphingolipid metabolism | FA Deg/Sphin Meta |
| Fatty acid degradation/Glyoxylate and dicarboxylate metabolism | FA Deg/Glyo,Dicar Meta |
| Fatty acid degradation/Propanoate metabolism | FA Deg/Prop Meta |
| Fatty acid degradation/Vitamin B6 metabolism | FA Deg/VB6 Meta |
| Fatty acid degradation/Biotin metabolism | FA Deg/Biotin Meta |
| Fatty acid degradation/Aminoacyl-tRNA biosynthesis | FA Deg/Amino-tRNA Bio |
| Steroid biosynthesis/Valine, leucine and isoleucine degradation | Steroid Bio/Val,Leu,Ile Deg |
| Steroid biosynthesis/Valine, leucine and isoleucine biosynthesis | Steroid Bio/Val,Leu,Ile Bio |
| Steroid biosynthesis/N-Glycan biosynthesis | Steroid Bio/N-Glycan Bio |
| Steroid biosynthesis/Other glycan degradation | Steroid Bio/Other glycan Deg |
| Steroid biosynthesis/Amino sugar and nucleotide sugar metabolism | Steroid Bio/AS,NS Meta |
| Steroid biosynthesis/Glycosaminoglycan biosynthesis - keratan sulfate | Steroid Bio/Kera sulf Bio |
| Steroid biosynthesis/Terpenoid backbone biosynthesis | Steroid Bio/TB Bio |
| Primary bile acid biosynthesis/Tyrosine metabolism | Prim BA Bio/Tyr Meta |
| Primary bile acid biosynthesis/Phenylalanine, tyrosine and tryptophan biosynthesis | Prim BA Bio/Phe,Tyr,Trp Bio |
| Primary bile acid biosynthesis/Taurine and hypotaurine metabolism | Prim BA Bio/Taur,hypo Meta |
| Primary bile acid biosynthesis/D-Amino acid metabolism | Prim BA Bio/D-AA Meta |
| Primary bile acid biosynthesis/Starch and sucrose metabolism | Prim BA Bio/Star,Sucr Meta |
| Primary bile acid biosynthesis/Arachidonic acid metabolism | Prim BA Bio/Arach-acid Meta |
| Primary bile acid biosynthesis/alpha-Linolenic acid metabolism | Prim BA Bio/α-LA Meta |
| Primary bile acid biosynthesis/Butanoate metabolism | Prim BA Bio/Buta Meta |
| Primary bile acid biosynthesis/Nitrogen metabolism | Prim BA Bio/Nitr Meta |
| Primary bile acid biosynthesis/Drug metabolism - other enzymes | Prim BA Bio/Drug-other enzy |
| Ubiquinone and other terpenoid-quinone biosynthesis/Pyrimidine metabolism | Quinone Bio/Pyrim Meta |
| Ubiquinone and other terpenoid-quinone biosynthesis/Cysteine and methionine metabolism | Quinone Bio/Cys,Met Meta |
| Ubiquinone and other terpenoid-quinone biosynthesis/Valine, leucine and isoleucine biosynthesis | Quinone Bio/Val,Leu,Ile Bio |
| Ubiquinone and other terpenoid-quinone biosynthesis/Lysine degradation | Quinone Bio/Lys Deg |
| Ubiquinone and other terpenoid-quinone biosynthesis/Phosphonate and phosphinate metabolism | Quinone Bio/Phos, Phos Meta |
| Ubiquinone and other terpenoid-quinone biosynthesis/Selenocompound metabolism | Quinone Bio/Se-com Meta |
| Ubiquinone and other terpenoid-quinone biosynthesis/Glutathione metabolism | Quinone Bio/GSH Meta |
| Ubiquinone and other terpenoid-quinone biosynthesis/Various types of N-glycan biosynthesis | Quinone Bio/Vari N-glycan Bio |
| Ubiquinone and other terpenoid-quinone biosynthesis/Neomycin, kanamycin and gentamicin biosynthesis | Quinone Bio/Neo,Kana,gentamicin Bio |
| Ubiquinone and other terpenoid-quinone biosynthesis/Glycosaminoglycan degradation | Quinone Bio/GAG Deg |
| Ubiquinone and other terpenoid-quinone biosynthesis/Glycosaminoglycan biosynthesis - chondroitin sulfate/dermatan sulfate | Quinone Bio/Chon sulf/Derm sulf Bio |
| Ubiquinone and other terpenoid-quinone biosynthesis/Glycosaminoglycan biosynthesis - keratan sulfate | Quinone Bio/Kera sulf Bio |
| Ubiquinone and other terpenoid-quinone biosynthesis/Inositol phosphate metabolism | Quinone Bio/IP Meta |
| Ubiquinone and other terpenoid-quinone biosynthesis/Sphingolipid metabolism | Quinone Bio/Sphin Meta |
| Ubiquinone and other terpenoid-quinone biosynthesis/Glycosphingolipid biosynthesis - ganglio series | Quinone Bio/Ganglio Bio |
| Ubiquinone and other terpenoid-quinone biosynthesis/Glyoxylate and dicarboxylate metabolism | Quinone Bio/Glyo,Dicar Meta |
| Ubiquinone and other terpenoid-quinone biosynthesis/Propanoate metabolism | Quinone Bio/Prop Meta |
| Ubiquinone and other terpenoid-quinone biosynthesis/Biotin metabolism | Quinone Bio/Biotin Meta |
| Steroid hormone biosynthesis/Caffeine metabolism | Steroid horm Bio/Caff Meta |
| Arginine biosynthesis/Alanine, aspartate and glutamate metabolism | Arg Bio/Ala, Asp,Glu Meta |
| Arginine biosynthesis/Glycine, serine and threonine metabolism | Arg Bio/Gly,Ser,Thr Meta |
| Arginine biosynthesis/Phenylalanine metabolism | Arg Bio/Phe Meta |
| Arginine biosynthesis/Taurine and hypotaurine metabolism | Arg Bio/Taur,hypo Meta |
| Arginine biosynthesis/Mucin type O-glycan biosynthesis | Arg Bio/Muc type O-glycan Bio |
| Arginine biosynthesis/Glycosaminoglycan biosynthesis - heparan sulfate/heparin | Arg Bio/hepa sulf/heparin Bio |
| Arginine biosynthesis/Glycerolipid metabolism | Arg Bio/GL Meta |
| Arginine biosynthesis/Glycosphingolipid biosynthesis - lacto and neolacto series | Arg Bio/lacto,neol Bio |
| Arginine biosynthesis/Thiamine metabolism | Arg Bio/Thia Meta |
| Arginine biosynthesis/Lipoic acid metabolism | Arg Bio/LipoicA Meta |
| Arginine biosynthesis/Folate biosynthesis | Arg Bio/Folate Bio |
| Purine metabolism/Histidine metabolism | Purine Meta/His Meta |
| Purine metabolism/Phenylalanine metabolism | Purine Meta/Phe Meta |
| Purine metabolism/Tryptophan metabolism | Purine Meta/Trp Meta |
| Purine metabolism/beta-Alanine metabolism | Purine Meta/β-Ala Meta |
| Purine metabolism/Mucin type O-glycan biosynthesis | Purine Meta/Muc type O-glycan Bio |
| Purine metabolism/Mannose type O-glycan biosynthesis | Purine Meta/Mann type O-glycan Bio |
| Purine metabolism/Ether lipid metabolism | Purine Meta/EL Meta |
| Purine metabolism/Glycosphingolipid biosynthesis - globo and isoglobo series | Purine Meta/Globo,Isog Bio |
| Purine metabolism/One carbon pool by folate | Purine Meta/One C pool by folate |
| Purine metabolism/Nicotinate and nicotinamide metabolism | Purine Meta/Nico,NA Meta |
| Purine metabolism/Folate biosynthesis | Purine Meta/Folate Bio |
| Caffeine metabolism/Retinol metabolism | Caff Meta/VA Meta |
| Pyrimidine metabolism/Cysteine and methionine metabolism | Pyrim Meta/Cys,Met Meta |
| Pyrimidine metabolism/Valine, leucine and isoleucine biosynthesis | Pyrim Meta/Val,Leu,Ile Bio |
| Pyrimidine metabolism/Lysine degradation | Pyrim Meta/Lys Deg |
| Pyrimidine metabolism/Phosphonate and phosphinate metabolism | Pyrim Meta/Phos, Phos Meta |
| Pyrimidine metabolism/Glutathione metabolism | Pyrim Meta/GSH Meta |
| Pyrimidine metabolism/Neomycin, kanamycin and gentamicin biosynthesis | Pyrim Meta/Neo,Kana,gentamicin Bio |
| Pyrimidine metabolism/Glycosaminoglycan degradation | Pyrim Meta/GAG Deg |
| Pyrimidine metabolism/Glycosaminoglycan biosynthesis - chondroitin sulfate/dermatan sulfate | Pyrim Meta/Chon sulf/Derm sulf Bio |
| Pyrimidine metabolism/Inositol phosphate metabolism | Pyrim Meta/IP Meta |
| Pyrimidine metabolism/Sphingolipid metabolism | Pyrim Meta/Sphin Meta |
| Pyrimidine metabolism/Glycosphingolipid biosynthesis - ganglio series | Pyrim Meta/Ganglio Bio |
| Pyrimidine metabolism/Glyoxylate and dicarboxylate metabolism | Pyrim Meta/Glyo,Dicar Meta |
| Pyrimidine metabolism/Propanoate metabolism | Pyrim Meta/Prop Meta |
| Pyrimidine metabolism/Biotin metabolism | Pyrim Meta/Biotin Meta |
| Alanine, aspartate and glutamate metabolism/Glycine, serine and threonine metabolism | Ala, Asp,Glu Meta/Gly,Ser,Thr Meta |
| Alanine, aspartate and glutamate metabolism/Histidine metabolism | Ala, Asp,Glu Meta/His Meta |
| Alanine, aspartate and glutamate metabolism/Phenylalanine metabolism | Ala, Asp,Glu Meta/Phe Meta |
| Alanine, aspartate and glutamate metabolism/Taurine and hypotaurine metabolism | Ala, Asp,Glu Meta/Taur,hypo Meta |
| Alanine, aspartate and glutamate metabolism/Mucin type O-glycan biosynthesis | Ala, Asp,Glu Meta/Muc type O-glycan Bio |
| Alanine, aspartate and glutamate metabolism/Glycosaminoglycan biosynthesis - heparan sulfate/heparin | Ala, Asp,Glu Meta/hepa sulf/heparin Bio |
| Alanine, aspartate and glutamate metabolism/Glycerolipid metabolism | Ala, Asp,Glu Meta/GL Meta |
| Alanine, aspartate and glutamate metabolism/Ether lipid metabolism | Ala, Asp,Glu Meta/EL Meta |
| Alanine, aspartate and glutamate metabolism/Glycosphingolipid biosynthesis - lacto and neolacto series | Ala, Asp,Glu Meta/lacto,neol Bio |
| Alanine, aspartate and glutamate metabolism/Glycosphingolipid biosynthesis - globo and isoglobo series | Ala, Asp,Glu Meta/Globo,Isog Bio |
| Alanine, aspartate and glutamate metabolism/Thiamine metabolism | Ala, Asp,Glu Meta/Thia Meta |
| Alanine, aspartate and glutamate metabolism/Nicotinate and nicotinamide metabolism | Ala, Asp,Glu Meta/Nico,NA Meta |
| Alanine, aspartate and glutamate metabolism/Lipoic acid metabolism | Ala, Asp,Glu Meta/LipoicA Meta |
| Alanine, aspartate and glutamate metabolism/Folate biosynthesis | Ala, Asp,Glu Meta/Folate Bio |
| Glycine, serine and threonine metabolism/Phenylalanine metabolism | Gly,Ser,Thr Meta/Phe Meta |
| Glycine, serine and threonine metabolism/Taurine and hypotaurine metabolism | Gly,Ser,Thr Meta/Taur,hypo Meta |
| Glycine, serine and threonine metabolism/Mucin type O-glycan biosynthesis | Gly,Ser,Thr Meta/Muc type O-glycan Bio |
| Glycine, serine and threonine metabolism/Glycosaminoglycan biosynthesis - heparan sulfate/heparin | Gly,Ser,Thr Meta/hepa sulf/heparin Bio |
| Glycine, serine and threonine metabolism/Glycerolipid metabolism | Gly,Ser,Thr Meta/GL Meta |
| Glycine, serine and threonine metabolism/Ether lipid metabolism | Gly,Ser,Thr Meta/EL Meta |
| Glycine, serine and threonine metabolism/Glycosphingolipid biosynthesis - lacto and neolacto series | Gly,Ser,Thr Meta/lacto,neol Bio |
| Glycine, serine and threonine metabolism/Glycosphingolipid biosynthesis - globo and isoglobo series | Gly,Ser,Thr Meta/Globo,Isog Bio |
| Glycine, serine and threonine metabolism/Thiamine metabolism | Gly,Ser,Thr Meta/Thia Meta |
| Glycine, serine and threonine metabolism/Lipoic acid metabolism | Gly,Ser,Thr Meta/LipoicA Meta |
| Glycine, serine and threonine metabolism/Folate biosynthesis | Gly,Ser,Thr Meta/Folate Bio |
| Cysteine and methionine metabolism/Lysine degradation | Cys,Met Meta/Lys Deg |
| Cysteine and methionine metabolism/Phosphonate and phosphinate metabolism | Cys,Met Meta/Phos, Phos Meta |
| Cysteine and methionine metabolism/Neomycin, kanamycin and gentamicin biosynthesis | Cys,Met Meta/Neo,Kana,gentamicin Bio |
| Cysteine and methionine metabolism/Glycosaminoglycan degradation | Cys,Met Meta/GAG Deg |
| Cysteine and methionine metabolism/Inositol phosphate metabolism | Cys,Met Meta/IP Meta |
| Cysteine and methionine metabolism/Sphingolipid metabolism | Cys,Met Meta/Sphin Meta |
| Cysteine and methionine metabolism/Glycosphingolipid biosynthesis - ganglio series | Cys,Met Meta/Ganglio Bio |
| Cysteine and methionine metabolism/Glyoxylate and dicarboxylate metabolism | Cys,Met Meta/Glyo,Dicar Meta |
| Cysteine and methionine metabolism/Propanoate metabolism | Cys,Met Meta/Prop Meta |
| Cysteine and methionine metabolism/Vitamin B6 metabolism | Cys,Met Meta/VB6 Meta |
| Cysteine and methionine metabolism/Biotin metabolism | Cys,Met Meta/Biotin Meta |
| Valine, leucine and isoleucine degradation/Valine, leucine and isoleucine biosynthesis | Val,Leu,Ile Deg/Val,Leu,Ile Bio |
| Valine, leucine and isoleucine degradation/N-Glycan biosynthesis | Val,Leu,Ile Deg/N-Glycan Bio |
| Valine, leucine and isoleucine degradation/Other glycan degradation | Val,Leu,Ile Deg/Other glycan Deg |
| Valine, leucine and isoleucine degradation/Various types of N-glycan biosynthesis | Val,Leu,Ile Deg/Vari N-glycan Bio |
| Valine, leucine and isoleucine degradation/Amino sugar and nucleotide sugar metabolism | Val,Leu,Ile Deg/AS,NS Meta |
| Valine, leucine and isoleucine degradation/Glycosaminoglycan biosynthesis - chondroitin sulfate/dermatan sulfate | Val,Leu,Ile Deg/Chon sulf/Derm sulf Bio |
| Valine, leucine and isoleucine degradation/Glycosaminoglycan biosynthesis - keratan sulfate | Val,Leu,Ile Deg/Kera sulf Bio |
| Valine, leucine and isoleucine degradation/Glycosylphosphatidylinositol (GPI)-anchor biosynthesis | Val,Leu,Ile Deg/Anchor Bio |
| Valine, leucine and isoleucine degradation/Aminoacyl-tRNA biosynthesis | Val,Leu,Ile Deg/Amino-tRNA Bio |
| Valine, leucine and isoleucine biosynthesis/Lysine degradation | Val, Leu,Ile Bio/Lys Deg |
| Valine, leucine and isoleucine biosynthesis/Selenocompound metabolism | Val, Leu,Ile Bio/Se-com Meta |
| Valine, leucine and isoleucine biosynthesis/Glutathione metabolism | Val, Leu,Ile Bio/GSH Meta |
| Valine, leucine and isoleucine biosynthesis/N-Glycan biosynthesis | Val, Leu,Ile Bio/N-Glycan Bio |
| Valine, leucine and isoleucine biosynthesis/Other glycan degradation | Val, Leu,Ile Bio/Other glycan Deg |
| Valine, leucine and isoleucine biosynthesis/Various types of N-glycan biosynthesis | Val, Leu,Ile Bio/Vari N-glycan Bio |
| Valine, leucine and isoleucine biosynthesis/Amino sugar and nucleotide sugar metabolism | Val, Leu,Ile Bio/AS,NS Meta |
| Valine, leucine and isoleucine biosynthesis/Glycosaminoglycan biosynthesis - chondroitin sulfate/dermatan sulfate | Val, Leu,Ile Bio/Chon sulf/Derm sulf Bio |
| Valine, leucine and isoleucine biosynthesis/Glycosaminoglycan biosynthesis - keratan sulfate | Val, Leu,Ile Bio/Kera sulf Bio |
| Valine, leucine and isoleucine biosynthesis/Inositol phosphate metabolism | Val, Leu,Ile Bio/IP Meta |
| Valine, leucine and isoleucine biosynthesis/Glycosylphosphatidylinositol (GPI)-anchor biosynthesis | Val, Leu,Ile Bio/Anchor Bio |
| Valine, leucine and isoleucine biosynthesis/Glyoxylate and dicarboxylate metabolism | Val, Leu,Ile Bio/Glyo,Dicar Meta |
| Valine, leucine and isoleucine biosynthesis/Propanoate metabolism | Val, Leu,Ile Bio/Prop Meta |
| Valine, leucine and isoleucine biosynthesis/Biotin metabolism | Val, Leu,Ile Bio/Biotin Meta |
| Valine, leucine and isoleucine biosynthesis/Terpenoid backbone biosynthesis | Val, Leu,Ile Bio/TB Bio |
| Valine, leucine and isoleucine biosynthesis/Aminoacyl-tRNA biosynthesis | Val, Leu,Ile Bio/Amino-tRNA Bio |
| Lysine degradation/Phosphonate and phosphinate metabolism | Lys Deg/Phos, Phos Meta |
| Lysine degradation/Glutathione metabolism | Lys Deg/GSH Meta |
| Lysine degradation/Neomycin, kanamycin and gentamicin biosynthesis | Lys Deg/Neo,Kana,gentamicin Bio |
| Lysine degradation/Glycosaminoglycan degradation | Lys Deg/GAG Deg |
| Lysine degradation/Glycosaminoglycan biosynthesis - chondroitin sulfate/dermatan sulfate | Lys Deg/Chon sulf/Derm sulf Bio |
| Lysine degradation/Inositol phosphate metabolism | Lys Deg/IP Meta |
| Lysine degradation/Sphingolipid metabolism | Lys Deg/Sphin Meta |
| Lysine degradation/Glycosphingolipid biosynthesis - ganglio series | Lys Deg/Ganglio Bio |
| Lysine degradation/Glyoxylate and dicarboxylate metabolism | Lys Deg/Glyo,Dicar Meta |
| Lysine degradation/Propanoate metabolism | Lys Deg/Prop Meta |
| Lysine degradation/Vitamin B6 metabolism | Lys Deg/VB6 Meta |
| Lysine degradation/Biotin metabolism | Lys Deg/Biotin Meta |
| Arginine and proline metabolism/Phosphonate and phosphinate metabolism | Arg,Pro Meta/Phos, Phos Meta |
| Arginine and proline metabolism/Other types of O-glycan biosynthesis | Arg,Pro Meta/Other O-glycan Bio |
| Arginine and proline metabolism/Neomycin, kanamycin and gentamicin biosynthesis | Arg,Pro Meta/Neo,Kana,gentamicin Bio |
| Arginine and proline metabolism/Glycosaminoglycan degradation | Arg,Pro Meta/GAG Deg |
| Arginine and proline metabolism/Glycosphingolipid biosynthesis - ganglio series | Arg,Pro Meta/Ganglio Bio |
| Arginine and proline metabolism/Pyruvate metabolism | Arg,Pro Meta/Pyru Meta |
| Arginine and proline metabolism/One carbon pool by folate | Arg,Pro Meta/One C pool by folate |
| Arginine and proline metabolism/Vitamin B6 metabolism | Arg,Pro Meta/VB6 Meta |
| Arginine and proline metabolism/Pantothenate and CoA biosynthesis | Arg,Pro Meta/Pant and CoA Bio |
| Histidine metabolism/Phenylalanine metabolism | His Meta/Phe Meta |
| Histidine metabolism/Tryptophan metabolism | His Meta/Trp Meta |
| Histidine metabolism/beta-Alanine metabolism | His Meta/β-Ala Meta |
| Histidine metabolism/Mucin type O-glycan biosynthesis | His Meta/Muc type O-glycan Bio |
| Histidine metabolism/Other types of O-glycan biosynthesis | His Meta/Other O-glycan Bio |
| Histidine metabolism/Mannose type O-glycan biosynthesis | His Meta/Mann type O-glycan Bio |
| Histidine metabolism/Neomycin, kanamycin and gentamicin biosynthesis | His Meta/Neo,Kana,gentamicin Bio |
| Histidine metabolism/Glycosaminoglycan biosynthesis - heparan sulfate/heparin | His Meta/hepa sulf/heparin Bio |
| Histidine metabolism/Glycerophospholipid metabolism | His Meta/Glycero-PL Meta |
| Histidine metabolism/Ether lipid metabolism | His Meta/EL Meta |
| Histidine metabolism/Glycosphingolipid biosynthesis - lacto and neolacto series | His Meta/lacto,neol Bio |
| Histidine metabolism/Glycosphingolipid biosynthesis - globo and isoglobo series | His Meta/Globo,Isog Bio |
| Histidine metabolism/One carbon pool by folate | His Meta/One C pool by folate |
| Histidine metabolism/Vitamin B6 metabolism | His Meta/VB6 Meta |
| Histidine metabolism/Nicotinate and nicotinamide metabolism | His Meta/Nico,NA Meta |
| Histidine metabolism/Pantothenate and CoA biosynthesis | His Meta/Pant and CoA Bio |
| Histidine metabolism/Folate biosynthesis | His Meta/Folate Bio |
| Tyrosine metabolism/Phenylalanine, tyrosine and tryptophan biosynthesis | Tyr Meta/Phe,Tyr,Trp Bio |
| Tyrosine metabolism/Taurine and hypotaurine metabolism | Tyr Meta/Taur,hypo Meta |
| Tyrosine metabolism/D-Amino acid metabolism | Tyr Meta/D-AA Meta |
| Tyrosine metabolism/Starch and sucrose metabolism | Tyr Meta/Star,Sucr Meta |
| Tyrosine metabolism/Arachidonic acid metabolism | Tyr Meta/Arach-acid Meta |
| Tyrosine metabolism/alpha-Linolenic acid metabolism | Tyr Meta/α-LA Meta |
| Tyrosine metabolism/Butanoate metabolism | Tyr Meta/Buta Meta |
| Tyrosine metabolism/Nitrogen metabolism | Tyr Meta/Nitr Meta |
| Tyrosine metabolism/Drug metabolism - other enzymes | Tyr Meta/Drug-other enzy |
| Phenylalanine metabolism/Tryptophan metabolism | Phe Meta/Trp Meta |
| Phenylalanine metabolism/Mucin type O-glycan biosynthesis | Phe Meta/Muc type O-glycan Bio |
| Phenylalanine metabolism/Mannose type O-glycan biosynthesis | Phe Meta/Mann type O-glycan Bio |
| Phenylalanine metabolism/Glycosaminoglycan biosynthesis - heparan sulfate/heparin | Phe Meta/hepa sulf/heparin Bio |
| Phenylalanine metabolism/Glycerolipid metabolism | Phe Meta/GL Meta |
| Phenylalanine metabolism/Ether lipid metabolism | Phe Meta/EL Meta |
| Phenylalanine metabolism/Glycosphingolipid biosynthesis - lacto and neolacto series | Phe Meta/lacto,neol Bio |
| Phenylalanine metabolism/Glycosphingolipid biosynthesis - globo and isoglobo series | Phe Meta/Globo,Isog Bio |
| Phenylalanine metabolism/One carbon pool by folate | Phe Meta/One C pool by folate |
| Phenylalanine metabolism/Nicotinate and nicotinamide metabolism | Phe Meta/Nico,NA Meta |
| Phenylalanine metabolism/Lipoic acid metabolism | Phe Meta/LipoicA Meta |
| Phenylalanine metabolism/Folate biosynthesis | Phe Meta/Folate Bio |
| Tryptophan metabolism/beta-Alanine metabolism | Trp Meta/β-Ala Meta |
| Tryptophan metabolism/Mucin type O-glycan biosynthesis | Trp Meta/Muc type O-glycan Bio |
| Tryptophan metabolism/Other types of O-glycan biosynthesis | Trp Meta/Other O-glycan Bio |
| Tryptophan metabolism/Mannose type O-glycan biosynthesis | Trp Meta/Mann type O-glycan Bio |
| Tryptophan metabolism/Neomycin, kanamycin and gentamicin biosynthesis | Trp Meta/Neo,Kana,gentamicin Bio |
| Tryptophan metabolism/Glycerophospholipid metabolism | Trp Meta/Glycero-PL Meta |
| Tryptophan metabolism/Glycosphingolipid biosynthesis - globo and isoglobo series | Trp Meta/Globo,Isog Bio |
| Tryptophan metabolism/One carbon pool by folate | Trp Meta/One C pool by folate |
| Tryptophan metabolism/Vitamin B6 metabolism | Trp Meta/VB6 Meta |
| Tryptophan metabolism/Nicotinate and nicotinamide metabolism | Trp Meta/Nico,NA Meta |
| Tryptophan metabolism/Pantothenate and CoA biosynthesis | Trp Meta/Pant and CoA Bio |
| Phenylalanine, tyrosine and tryptophan biosynthesis/Taurine and hypotaurine metabolism | Phe,Tyr,Trp Bio/Taur,hypo Meta |
| Phenylalanine, tyrosine and tryptophan biosynthesis/D-Amino acid metabolism | Phe,Tyr,Trp Bio/D-AA Meta |
| Phenylalanine, tyrosine and tryptophan biosynthesis/Starch and sucrose metabolism | Phe,Tyr,Trp Bio/Star,Sucr Meta |
| Phenylalanine, tyrosine and tryptophan biosynthesis/Arachidonic acid metabolism | Phe,Tyr,Trp Bio/Arach-acid Meta |
| Phenylalanine, tyrosine and tryptophan biosynthesis/alpha-Linolenic acid metabolism | Phe,Tyr,Trp Bio/α-LA Meta |
| Phenylalanine, tyrosine and tryptophan biosynthesis/Butanoate metabolism | Phe,Tyr,Trp Bio/Buta Meta |
| Phenylalanine, tyrosine and tryptophan biosynthesis/Porphyrin metabolism | Phe,Tyr,Trp Bio/Porphyrin metabolism |
| Phenylalanine, tyrosine and tryptophan biosynthesis/Nitrogen metabolism | Phe,Tyr,Trp Bio/Nitr Meta |
| Phenylalanine, tyrosine and tryptophan biosynthesis/Drug metabolism - other enzymes | Phe,Tyr,Trp Bio/Drug-other enzy |
| beta-Alanine metabolism/Mucin type O-glycan biosynthesis | β-Ala Meta/Muc type O-glycan Bio |
| beta-Alanine metabolism/Mannose type O-glycan biosynthesis | β-Ala Meta/Mann type O-glycan Bio |
| beta-Alanine metabolism/Neomycin, kanamycin and gentamicin biosynthesis | β-Ala Meta/Neo,Kana,gentamicin Bio |
| beta-Alanine metabolism/Glycerophospholipid metabolism | β-Ala Meta/Glycero-PL Meta |
| beta-Alanine metabolism/Glycosphingolipid biosynthesis - globo and isoglobo series | β-Ala Meta/Globo,Isog Bio |
| beta-Alanine metabolism/One carbon pool by folate | β-Ala Meta/One C pool by folate |
| beta-Alanine metabolism/Vitamin B6 metabolism | β-Ala Meta/VB6 Meta |
| beta-Alanine metabolism/Nicotinate and nicotinamide metabolism | β-Ala Meta/Nico,NA Meta |
| beta-Alanine metabolism/Pantothenate and CoA biosynthesis | β-Ala Meta/Pant and CoA Bio |
| Taurine and hypotaurine metabolism/D-Amino acid metabolism | Taur,hypo Meta/D-AA Meta |
| Taurine and hypotaurine metabolism/Starch and sucrose metabolism | Taur,hypo Meta/Star,Sucr Meta |
| Taurine and hypotaurine metabolism/Glycosaminoglycan biosynthesis - heparan sulfate/heparin | Taur,hypo Meta/hepa sulf/heparin Bio |
| Taurine and hypotaurine metabolism/Glycerolipid metabolism | Taur,hypo Meta/GL Meta |
| Taurine and hypotaurine metabolism/Arachidonic acid metabolism | Taur,hypo Meta/Arach-acid Meta |
| Taurine and hypotaurine metabolism/alpha-Linolenic acid metabolism | Taur,hypo Meta/α-LA Meta |
| Taurine and hypotaurine metabolism/Glycosphingolipid biosynthesis - lacto and neolacto series | Taur,hypo Meta/lacto,neol Bio |
| Taurine and hypotaurine metabolism/Butanoate metabolism | Taur,hypo Meta/Buta Meta |
| Taurine and hypotaurine metabolism/Thiamine metabolism | Taur,hypo Meta/Thia Meta |
| Taurine and hypotaurine metabolism/Lipoic acid metabolism | Taur,hypo Meta/LipoicA Meta |
| Taurine and hypotaurine metabolism/Folate biosynthesis | Taur,hypo Meta/Folate Bio |
| Taurine and hypotaurine metabolism/Drug metabolism - other enzymes | Taur,hypo Meta/Drug-other enzy |
| Phosphonate and phosphinate metabolism/Other types of O-glycan biosynthesis | Phos, Phos Meta/Other O-glycan Bio |
| Phosphonate and phosphinate metabolism/Mannose type O-glycan biosynthesis | Phos, Phos Meta/Mann type O-glycan Bio |
| Phosphonate and phosphinate metabolism/Neomycin, kanamycin and gentamicin biosynthesis | Phos, Phos Meta/Neo,Kana,gentamicin Bio |
| Phosphonate and phosphinate metabolism/Glycosaminoglycan degradation | Phos, Phos Meta/GAG Deg |
| Phosphonate and phosphinate metabolism/Inositol phosphate metabolism | Phos, Phos Meta/IP Meta |
| Phosphonate and phosphinate metabolism/Glycerophospholipid metabolism | Phos, Phos Meta/Glycero-PL Meta |
| Phosphonate and phosphinate metabolism/Sphingolipid metabolism | Phos, Phos Meta/Sphin Meta |
| Phosphonate and phosphinate metabolism/Glycosphingolipid biosynthesis - ganglio series | Phos, Phos Meta/Ganglio Bio |
| Phosphonate and phosphinate metabolism/Pyruvate metabolism | Phos, Phos Meta/Pyru Meta |
| Phosphonate and phosphinate metabolism/Glyoxylate and dicarboxylate metabolism | Phos, Phos Meta/Glyo,Dicar Meta |
| Phosphonate and phosphinate metabolism/One carbon pool by folate | Phos, Phos Meta/One C pool by folate |
| Phosphonate and phosphinate metabolism/Vitamin B6 metabolism | Phos, Phos Meta/VB6 Meta |
| Phosphonate and phosphinate metabolism/Pantothenate and CoA biosynthesis | Phos, Phos Meta/Pant and CoA Bio |
| Phosphonate and phosphinate metabolism/Biotin metabolism | Phos, Phos Meta/Biotin Meta |
| Selenocompound metabolism/Glutathione metabolism | Se-com Meta/GSH Meta |
| Selenocompound metabolism/Various types of N-glycan biosynthesis | Se-com Meta/Vari N-glycan Bio |
| Selenocompound metabolism/Glycosaminoglycan biosynthesis - chondroitin sulfate/dermatan sulfate | Se-com Meta/Chon sulf/Derm sulf Bio |
| Selenocompound metabolism/Glycosaminoglycan biosynthesis - keratan sulfate | Se-com Meta/Kera sulf Bio |
| Selenocompound metabolism/Glycosylphosphatidylinositol (GPI)-anchor biosynthesis | Se-com Meta/Anchor Bio |
| Selenocompound metabolism/Propanoate metabolism | Se-com Meta/Prop Meta |
| Selenocompound metabolism/Biotin metabolism | Se-com Meta/Biotin Meta |
| Selenocompound metabolism/Aminoacyl-tRNA biosynthesis | Se-com Meta/Amino-tRNA Bio |
| D-Amino acid metabolism/Starch and sucrose metabolism | D-AA Meta/Star,Sucr Meta |
| D-Amino acid metabolism/Arachidonic acid metabolism | D-AA Meta/Arach-acid Meta |
| D-Amino acid metabolism/alpha-Linolenic acid metabolism | D-AA Meta/α-LA Meta |
| D-Amino acid metabolism/Butanoate metabolism | D-AA Meta/Buta Meta |
| D-Amino acid metabolism/Thiamine metabolism | D-AA Meta/Thia Meta |
| D-Amino acid metabolism/Lipoic acid metabolism | D-AA Meta/LipoicA Meta |
| D-Amino acid metabolism/Nitrogen metabolism | D-AA Meta/Nitr Meta |
| D-Amino acid metabolism/Drug metabolism - other enzymes | D-AA Meta/Drug-other enzy |
| Glutathione metabolism/Various types of N-glycan biosynthesis | GSH Meta/Vari N-glycan Bio |
| Glutathione metabolism/Glycosaminoglycan biosynthesis - chondroitin sulfate/dermatan sulfate | GSH Meta/Chon sulf/Derm sulf Bio |
| Glutathione metabolism/Glycosaminoglycan biosynthesis - keratan sulfate | GSH Meta/Kera sulf Bio |
| Glutathione metabolism/Inositol phosphate metabolism | GSH Meta/IP Meta |
| Glutathione metabolism/Glycosylphosphatidylinositol (GPI)-anchor biosynthesis | GSH Meta/Anchor Bio |
| Glutathione metabolism/Glyoxylate and dicarboxylate metabolism | GSH Meta/Glyo,Dicar Meta |
| Glutathione metabolism/Propanoate metabolism | GSH Meta/Prop Meta |
| Glutathione metabolism/Biotin metabolism | GSH Meta/Biotin Meta |
| Glutathione metabolism/Aminoacyl-tRNA biosynthesis | GSH Meta/Amino-tRNA Bio |
| Starch and sucrose metabolism/Arachidonic acid metabolism | Star,Sucr Meta/Arach-acid Meta |
| Starch and sucrose metabolism/alpha-Linolenic acid metabolism | Star,Sucr Meta/α-LA Meta |
| Starch and sucrose metabolism/Butanoate metabolism | Star,Sucr Meta/Buta Meta |
| Starch and sucrose metabolism/Thiamine metabolism | Star,Sucr Meta/Thia Meta |
| Starch and sucrose metabolism/Lipoic acid metabolism | Star,Sucr Meta/LipoicA Meta |
| Starch and sucrose metabolism/Drug metabolism - other enzymes | Star,Sucr Meta/Drug-other enzy |
| N-Glycan biosynthesis/Other glycan degradation | N-Glycan Bio/Other glycan Deg |
| N-Glycan biosynthesis/Amino sugar and nucleotide sugar metabolism | N-Glycan Bio/AS,NS Meta |
| N-Glycan biosynthesis/Terpenoid backbone biosynthesis | N-Glycan Bio/TB Bio |
| Other glycan degradation/Amino sugar and nucleotide sugar metabolism | Other glycan Deg/AS,NS Meta |
| Other glycan degradation/Glycosaminoglycan biosynthesis - keratan sulfate | Other glycan Deg/Kera sulf Bio |
| Other glycan degradation/Terpenoid backbone biosynthesis | Other glycan Deg/TB Bio |
| Other glycan degradation/Aminoacyl-tRNA biosynthesis | Other glycan Deg/Amino-tRNA Bio |
| Mucin type O-glycan biosynthesis/Glycosaminoglycan biosynthesis - heparan sulfate/heparin | Muc type O-glycan Bio/hepa sulf/heparin Bio |
| Mucin type O-glycan biosynthesis/Glycerolipid metabolism | Muc type O-glycan Bio/GL Meta |
| Mucin type O-glycan biosynthesis/Ether lipid metabolism | Muc type O-glycan Bio/EL Meta |
| Mucin type O-glycan biosynthesis/Glycosphingolipid biosynthesis - globo and isoglobo series | Muc type O-glycan Bio/Globo,Isog Bio |
| Mucin type O-glycan biosynthesis/Nicotinate and nicotinamide metabolism | Muc type O-glycan Bio/Nico,NA Meta |
| Mucin type O-glycan biosynthesis/Lipoic acid metabolism | Muc type O-glycan Bio/LipoicA Meta |
| Mucin type O-glycan biosynthesis/Folate biosynthesis | Muc type O-glycan Bio/Folate Bio |
| Various types of N-glycan biosynthesis/Glycosaminoglycan biosynthesis - chondroitin sulfate/dermatan sulfate | Vari N-glycan Bio/Chon sulf/Derm sulf Bio |
| Various types of N-glycan biosynthesis/Glycosaminoglycan biosynthesis - keratan sulfate | Vari N-glycan Bio/Kera sulf Bio |
| Various types of N-glycan biosynthesis/Glycosylphosphatidylinositol (GPI)-anchor biosynthesis | Vari N-glycan Bio/Anchor Bio |
| Various types of N-glycan biosynthesis/Propanoate metabolism | Vari N-glycan Bio/Prop Meta |
| Various types of N-glycan biosynthesis/Biotin metabolism | Vari N-glycan Bio/Biotin Meta |
| Various types of N-glycan biosynthesis/Aminoacyl-tRNA biosynthesis | Vari N-glycan Bio/Amino-tRNA Bio |
| Other types of O-glycan biosynthesis/Mannose type O-glycan biosynthesis | Other O-glycan Bio/Mann type O-glycan Bio |
| Other types of O-glycan biosynthesis/Neomycin, kanamycin and gentamicin biosynthesis | Other O-glycan Bio/Neo,Kana,gentamicin Bio |
| Other types of O-glycan biosynthesis/Glycerophospholipid metabolism | Other O-glycan Bio/Glycero-PL Meta |
| Other types of O-glycan biosynthesis/Glycosphingolipid biosynthesis - ganglio series | Other O-glycan Bio/Ganglio Bio |
| Other types of O-glycan biosynthesis/Pyruvate metabolism | Other O-glycan Bio/Pyru Meta |
| Other types of O-glycan biosynthesis/One carbon pool by folate | Other O-glycan Bio/One C pool by folate |
| Other types of O-glycan biosynthesis/Vitamin B6 metabolism | Other O-glycan Bio/VB6 Meta |
| Other types of O-glycan biosynthesis/Pantothenate and CoA biosynthesis | Other O-glycan Bio/Pant and CoA Bio |
| Mannose type O-glycan biosynthesis/Neomycin, kanamycin and gentamicin biosynthesis | Mann type O-glycan Bio /Neo,Kana,gentamicin Bio |
| Mannose type O-glycan biosynthesis/Glycerophospholipid metabolism | Mann type O-glycan Bio /Glycero-PL Meta |
| Mannose type O-glycan biosynthesis/Ether lipid metabolism | Mann type O-glycan Bio /EL Meta |
| Mannose type O-glycan biosynthesis/Glycosphingolipid biosynthesis - globo and isoglobo series | Mann type O-glycan Bio /Globo,Isog Bio |
| Mannose type O-glycan biosynthesis/Glycosphingolipid biosynthesis - ganglio series | Mann type O-glycan Bio /Ganglio Bio |
| Mannose type O-glycan biosynthesis/Pyruvate metabolism | Mann type O-glycan Bio /Pyru Meta |
| Mannose type O-glycan biosynthesis/One carbon pool by folate | Mann type O-glycan Bio /One C pool by folate |
| Mannose type O-glycan biosynthesis/Vitamin B6 metabolism | Mann type O-glycan Bio /VB6 Meta |
| Mannose type O-glycan biosynthesis/Nicotinate and nicotinamide metabolism | Mann type O-glycan Bio /Nico,NA Meta |
| Mannose type O-glycan biosynthesis/Pantothenate and CoA biosynthesis | Mann type O-glycan Bio /Pant and CoA Bio |
| Amino sugar and nucleotide sugar metabolism/Glycosaminoglycan biosynthesis - keratan sulfate | AS,NS Meta/Kera sulf Bio |
| Amino sugar and nucleotide sugar metabolism/Terpenoid backbone biosynthesis | AS,NS Meta/TB Bio |
| Amino sugar and nucleotide sugar metabolism/Aminoacyl-tRNA biosynthesis | AS,NS Meta/Amino-tRNA Bio |
| Neomycin, kanamycin and gentamicin biosynthesis/Glycosaminoglycan degradation | Neo,Kana,gentamicin Bio/GAG Deg |
| Neomycin, kanamycin and gentamicin biosynthesis/Inositol phosphate metabolism | Neo,Kana,gentamicin Bio/IP Meta |
| Neomycin, kanamycin and gentamicin biosynthesis/Glycerophospholipid metabolism | Neo,Kana,gentamicin Bio/Glycero-PL Meta |
| Neomycin, kanamycin and gentamicin biosynthesis/Sphingolipid metabolism | Neo,Kana,gentamicin Bio/Sphin Meta |
| Neomycin, kanamycin and gentamicin biosynthesis/Glycosphingolipid biosynthesis - ganglio series | Neo,Kana,gentamicin Bio/Ganglio Bio |
| Neomycin, kanamycin and gentamicin biosynthesis/Pyruvate metabolism | Neo,Kana,gentamicin Bio/Pyru Meta |
| Neomycin, kanamycin and gentamicin biosynthesis/Glyoxylate and dicarboxylate metabolism | Neo,Kana,gentamicin Bio/Glyo,Dicar Meta |
| Neomycin, kanamycin and gentamicin biosynthesis/Propanoate metabolism | Neo,Kana,gentamicin Bio/Prop Meta |
| Neomycin, kanamycin and gentamicin biosynthesis/One carbon pool by folate | Neo,Kana,gentamicin Bio/One C pool by folate |
| Neomycin, kanamycin and gentamicin biosynthesis/Vitamin B6 metabolism | Neo,Kana,gentamicin Bio/VB6 Meta |
| Neomycin, kanamycin and gentamicin biosynthesis/Pantothenate and CoA biosynthesis | Neo,Kana,gentamicin Bio/Pant and CoA Bio |
| Neomycin, kanamycin and gentamicin biosynthesis/Biotin metabolism | Neo,Kana,gentamicin Bio/Biotin Meta |
| Glycosaminoglycan degradation/Inositol phosphate metabolism | GAG Deg/IP Meta |
| Glycosaminoglycan degradation/Sphingolipid metabolism | GAG Deg/Sphin Meta |
| Glycosaminoglycan degradation/Glycosphingolipid biosynthesis - ganglio series | GAG Deg/Ganglio Bio |
| Glycosaminoglycan degradation/Glyoxylate and dicarboxylate metabolism | GAG Deg/Glyo,Dicar Meta |
| Glycosaminoglycan degradation/Propanoate metabolism | GAG Deg/Prop Meta |
| Glycosaminoglycan degradation/Vitamin B6 metabolism | GAG Deg/VB6 Meta |
| Glycosaminoglycan degradation/Biotin metabolism | GAG Deg/Biotin Meta |
| Glycosaminoglycan biosynthesis - chondroitin sulfate/dermatan sulfate/Glycosaminoglycan biosynthesis - keratan sulfate | Chon sulf/Derm sulf Bio/Kera sulf Bio |
| Glycosaminoglycan biosynthesis - chondroitin sulfate/dermatan sulfate/Inositol phosphate metabolism | Chon sulf/Derm sulf Bio/IP Meta |
| Glycosaminoglycan biosynthesis - chondroitin sulfate/dermatan sulfate/Glycosylphosphatidylinositol (GPI)-anchor biosynthesis | Chon sulf/Derm sulf Bio/Anchor Bio |
| Glycosaminoglycan biosynthesis - chondroitin sulfate/dermatan sulfate/Glyoxylate and dicarboxylate metabolism | Chon sulf/Derm sulf Bio/Glyo,Dicar Meta |
| Glycosaminoglycan biosynthesis - chondroitin sulfate/dermatan sulfate/Propanoate metabolism | Chon sulf/Derm sulf Bio/Prop Meta |
| Glycosaminoglycan biosynthesis - chondroitin sulfate/dermatan sulfate/Biotin metabolism | Chon sulf/Derm sulf Bio/Biotin Meta |
| Glycosaminoglycan biosynthesis - chondroitin sulfate/dermatan sulfate/Aminoacyl-tRNA biosynthesis | Chon sulf/Derm sulf Bio/Amino-tRNA Bio |
| Glycosaminoglycan biosynthesis - keratan sulfate/Glycosylphosphatidylinositol (GPI)-anchor biosynthesis | Kera sulf Bio/Anchor Bio |
| Glycosaminoglycan biosynthesis - keratan sulfate/Propanoate metabolism | Kera sulf Bio/Prop Meta |
| Glycosaminoglycan biosynthesis - keratan sulfate/Biotin metabolism | Kera sulf Bio/Biotin Meta |
| Glycosaminoglycan biosynthesis - keratan sulfate/Terpenoid backbone biosynthesis | Kera sulf Bio/TB Bio |
| Glycosaminoglycan biosynthesis - keratan sulfate/Aminoacyl-tRNA biosynthesis | Kera sulf Bio/Amino-tRNA Bio |
| Glycosaminoglycan biosynthesis - heparan sulfate/heparin/Glycerolipid metabolism | hepa sulf/heparin Bio/GL Meta |
| Glycosaminoglycan biosynthesis - heparan sulfate/heparin/Ether lipid metabolism | hepa sulf/heparin Bio/EL Meta |
| Glycosaminoglycan biosynthesis - heparan sulfate/heparin/Glycosphingolipid biosynthesis - lacto and neolacto series | hepa sulf/heparin Bio/lacto,neol Bio |
| Glycosaminoglycan biosynthesis - heparan sulfate/heparin/Thiamine metabolism | hepa sulf/heparin Bio/Thia Meta |
| Glycosaminoglycan biosynthesis - heparan sulfate/heparin/Lipoic acid metabolism | hepa sulf/heparin Bio/LipoicA Meta |
| Glycosaminoglycan biosynthesis - heparan sulfate/heparin/Folate biosynthesis | hepa sulf/heparin Bio/Folate Bio |
| Glycerolipid metabolism/Ether lipid metabolism | GL Meta/EL Meta |
| Glycerolipid metabolism/Glycosphingolipid biosynthesis - lacto and neolacto series | GL Meta/lacto,neol Bio |
| Glycerolipid metabolism/Thiamine metabolism | GL Meta/Thia Meta |
| Glycerolipid metabolism/Lipoic acid metabolism | GL Meta/LipoicA Meta |
| Glycerolipid metabolism/Folate biosynthesis | GL Meta/Folate Bio |
| Inositol phosphate metabolism/Sphingolipid metabolism | IP Meta/Sphin Meta |
| Inositol phosphate metabolism/Glyoxylate and dicarboxylate metabolism | IP Meta/Glyo,Dicar Meta |
| Inositol phosphate metabolism/Propanoate metabolism | IP Meta/Prop Meta |
| Inositol phosphate metabolism/Vitamin B6 metabolism | IP Meta/VB6 Meta |
| Inositol phosphate metabolism/Biotin metabolism | IP Meta/Biotin Meta |
| Glycosylphosphatidylinositol (GPI)-anchor biosynthesis/Propanoate metabolism | Anchor Bio/Prop Meta |
| Glycosylphosphatidylinositol (GPI)-anchor biosynthesis/Aminoacyl-tRNA biosynthesis | Anchor Bio/Amino-tRNA Bio |
| Glycerophospholipid metabolism/Glycosphingolipid biosynthesis - globo and isoglobo series | Glycero-PL Meta/Globo,Isog Bio |
| Glycerophospholipid metabolism/Pyruvate metabolism | Glycero-PL Meta/Pyru Meta |
| Glycerophospholipid metabolism/One carbon pool by folate | Glycero-PL Meta/One C pool by folate |
| Glycerophospholipid metabolism/Vitamin B6 metabolism | Glycero-PL Meta/VB6 Meta |
| Glycerophospholipid metabolism/Pantothenate and CoA biosynthesis | Glycero-PL Meta/Pant and CoA Bio |
| Ether lipid metabolism/Glycosphingolipid biosynthesis - lacto and neolacto series | EL Meta/lacto,neol Bio |
| Ether lipid metabolism/Glycosphingolipid biosynthesis - globo and isoglobo series | EL Meta/Globo,Isog Bio |
| Ether lipid metabolism/Nicotinate and nicotinamide metabolism | EL Meta/Nico,NA Meta |
| Ether lipid metabolism/Folate biosynthesis | EL Meta/Folate Bio |
| Arachidonic acid metabolism/alpha-Linolenic acid metabolism | Arach-acid Meta/α-LA Meta |
| Arachidonic acid metabolism/Butanoate metabolism | Arach-acid Meta/Buta Meta |
| Arachidonic acid metabolism/Nitrogen metabolism | Arach-acid Meta/Nitr Meta |
| Arachidonic acid metabolism/Drug metabolism - other enzymes | Arach-acid Meta/Drug-other enzy |
| Linoleic acid metabolism/Metabolism of xenobiotics by cytochrome P450 | LA Meta/Xeno Meta by CYP450 |
| Linoleic acid metabolism/Drug metabolism - cytochrome P450 | LA Meta/Drug-CYP450 |
| alpha-Linolenic acid metabolism/Butanoate metabolism | α-LA Meta/Buta Meta |
| alpha-Linolenic acid metabolism/Nitrogen metabolism | α-LA Meta/Nitr Meta |
| alpha-Linolenic acid metabolism/Drug metabolism - other enzymes | α-LA Meta/Drug-other enzy |
| Sphingolipid metabolism/Glycosphingolipid biosynthesis - ganglio series | Sphin Meta/Ganglio Bio |
| Sphingolipid metabolism/Glyoxylate and dicarboxylate metabolism | Sphin Meta/Glyo,Dicar Meta |
| Sphingolipid metabolism/Vitamin B6 metabolism | Sphin Meta/VB6 Meta |
| Sphingolipid metabolism/Biotin metabolism | Sphin Meta/Biotin Meta |
| Glycosphingolipid biosynthesis - lacto and neolacto series/Thiamine metabolism | lacto,neol Bio/Thia Meta |
| Glycosphingolipid biosynthesis - lacto and neolacto series/Lipoic acid metabolism | lacto,neol Bio/LipoicA Meta |
| Glycosphingolipid biosynthesis - lacto and neolacto series/Folate biosynthesis | lacto,neol Bio/Folate Bio |
| Glycosphingolipid biosynthesis - globo and isoglobo series/One carbon pool by folate | Globo,Isog Bio/One C pool by folate |
| Glycosphingolipid biosynthesis - globo and isoglobo series/Nicotinate and nicotinamide metabolism | Globo,Isog Bio/Nico,NA Meta |
| Glycosphingolipid biosynthesis - globo and isoglobo series/Pantothenate and CoA biosynthesis | Globo,Isog Bio/Pant and CoA Bio |
| Glycosphingolipid biosynthesis - globo and isoglobo series/Folate biosynthesis | Globo,Isog Bio/Folate Bio |
| Glycosphingolipid biosynthesis - ganglio series/Pyruvate metabolism | Ganglio Bios/Pyru Meta |
| Glycosphingolipid biosynthesis - ganglio series/Glyoxylate and dicarboxylate metabolism | Ganglio Bios/Glyo,Dicar Meta |
| Glycosphingolipid biosynthesis - ganglio series/One carbon pool by folate | Ganglio Bios/One C pool by folate |
| Glycosphingolipid biosynthesis - ganglio series/Vitamin B6 metabolism | Ganglio Bios/VB6 Meta |
| Glycosphingolipid biosynthesis - ganglio series/Biotin metabolism | Ganglio Bios/Biotin Meta |
| Pyruvate metabolism/One carbon pool by folate | Pyru Meta/One C pool by folate |
| Pyruvate metabolism/Vitamin B6 metabolism | Pyru Meta/VB6 Meta |
| Pyruvate metabolism/Pantothenate and CoA biosynthesis | Pyru Meta/Pant and CoA Bio |
| Glyoxylate and dicarboxylate metabolism/Propanoate metabolism | Glyo,Dicar Meta/Prop Meta |
| Glyoxylate and dicarboxylate metabolism/Vitamin B6 metabolism | Glyo,Dicar Meta/VB6 Meta |
| Glyoxylate and dicarboxylate metabolism/Biotin metabolism | Glyo,Dicar Meta/Biotin Meta |
| Propanoate metabolism/Biotin metabolism | Prop Meta/Biotin Meta |
| Butanoate metabolism/Thiamine metabolism | Buta Meta/Thia Meta |
| Butanoate metabolism/Lipoic acid metabolism | Buta Meta/LipoicA Meta |
| Butanoate metabolism/Drug metabolism - other enzymes | Buta Meta/Drug-other enzy |
| One carbon pool by folate/Vitamin B6 metabolism | One C pool by folate/VB6 Meta |
| One carbon pool by folate/Pantothenate and CoA biosynthesis | One C pool by folate/Pant and CoA Bio |
| Thiamine metabolism/Lipoic acid metabolism | Thia Meta/LipoicA Meta |
| Vitamin B6 metabolism/Pantothenate and CoA biosynthesis | VB6 Meta/Pant and CoA Bio |
| Vitamin B6 metabolism/Biotin metabolism | VB6 Meta/Biotin Meta |
| Nicotinate and nicotinamide metabolism/Folate biosynthesis | Nico,NA Meta/Folate Bio |
| Lipoic acid metabolism/Folate biosynthesis | LipoicA Meta/Folate Bio |
| Terpenoid backbone biosynthesis/Aminoacyl-tRNA biosynthesis | TB Bio/Amino-tRNA Bio |
| Nitrogen metabolism/Drug metabolism - other enzymes | Nitr Meta /Drug-other enzy |
