# Supplementary material for: Prediction of prognosis and immunotherapy efficacy based on metabolic landscape in lung adenocarcinoma by bulk, single-cell RNA sequencing and Mendelian randomization analyses
Source: Aging (Albany NY). 2024 May 20;16(10):8772–809. doi: 10.18632/aging.205838 (PMC11164486; doi:10.18632/aging.205838)
Supplement: Supplementary Table 3 [file aging-16-205838-s004.pdf]

Supplementary Table 3. The list of primers used in this study.

Gene	Forward primer (5'-3')	Reverse primer (5'-3')
C1QTNF6	CACCATCCTGAAGGGTGACA	AGACCCTTTCGAAGAGCAGC
ECT2	ACCCCTAACAGCAATCGCAA	CAAGACTTTGGGGTGTCTCCA
SLC2A1	TGGCATCAACGCTGTCTTCT	CTAGCGCGATGGTCATGAGT
ANLN	CGCCTCAGACTCCTGGTTTT	GCTCCAGCAGTTTCTCCGTA
GAPDH	GGGAGCCAAAAGGGTCATCA	GCATGGACTGTGGTCATGAGT
LDHA	GCCGTCTTAATTTGGTCCAGC	ACTCCATACAGGCACACTGG
KRT8	ATCAACAACCTTAGGCGGCA	AGCTCCCGGATCTCCTCTTC
CD80	ATCACCATCCAAGTGTCCATACCTC	AGAAACATTGTGACCACAGGACAG
CD86	TGGCCTAGGGTACAGGCAACA	GCCCAGATAGAAGTGGCTCCAG
CD163	AAAAAGCCACAACAGGTCGC	CTTAAAGGCTGAACTCACTGGG
CD206	GGGGAAAGGTTACCCTGGTG	TCAAGGAAGGGTCGGATCGT
CD274	TCCTTGGCGATTATTTCCATGTC	GCCGACTACAAGCGAATTAC
18S(rRNA)	AAACGGCTACCACATCCAAG	CCTCCAATGGATCCTCGTTA
